# Tunable Electrochemical Entropy through Solvent Ordering by a Supramolecular Host

**DOI:** 10.1021/jacs.3c10145

**Published:** 2023-11-13

**Authors:** Kay T. Xia, Aravindh Rajan, Yogesh Surendranath, Robert G. Bergman, Kenneth N. Raymond, F. Dean Toste

**Affiliations:** † Chemical Sciences Division, Lawrence Berkeley National Laboratory, Berkeley, California 94720, United States; ‡ Department of Chemistry, University of California, Berkeley, California 94720, United States; § Palo Alto Research Center, 3333 Coyote Hill Road, Palo Alto, California 94304, United States; ∥ Department of Chemistry, Massachusetts Institute of Technology, Cambridge, Massachusetts 02139, United States

## Abstract

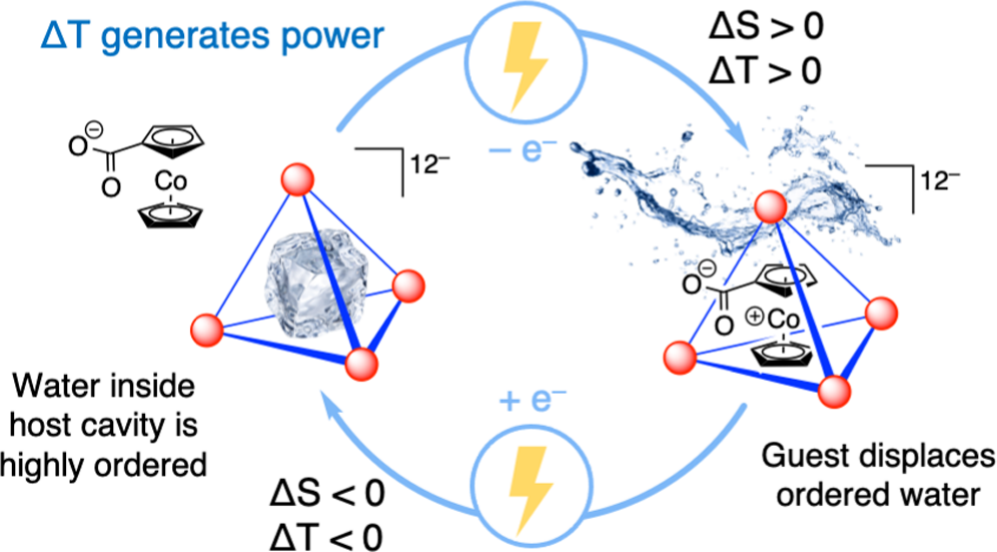

An aqueous electrochemically controlled host–guest encapsulation system demonstrates a large and synthetically tunable redox entropy change. Electrochemical entropy is the basis for thermally regenerative electrochemical cycles (TRECs), which utilize reversible electrochemical processes with large molar entropy changes for thermogalvanic waste-heat harvesting and electrochemical cooling, among other potential applications. A supramolecular host–guest system demonstrates a molar entropy change of 4 times that of the state-of-the-art aqueous TREC electrolyte potassium ferricyanide. Upon encapsulation of a guest, water molecules that structurally resemble amorphous ice are displaced from the host cavity, leveraging a change in the degrees of freedom and ordering of the solvent rather than the solvation of the redox-active species to increase entropy. The synthetic tunability of the host allows rational optimization of the system’s Δ*S*, showing a range of −51 to −101 cal mol^–1^ K^–1^ (−2.2 to −4.4 mV K^–1^) depending on ligand and metal vertex modifications, demonstrating the potential for rational design of high-entropy electrolytes and a new strategy to overcome theoretical limits on ion solvation reorganization entropy.

## Introduction

Liquid water demonstrates many unique properties due to hydrogen-bonding networks and strong directional interactions. Confinement of water within hydrophobic molecular cavities has been observed to disrupt the structure and preferential ordering of water molecules.^1−3^ Supramolecular hosts interact with encapsulated guests, including solvent molecules, through noncovalent electrostatic and steric effects, creating unique interior microenvironments divergent from bulk solution conditions.^4,5^ Encapsulation of small molecules within the [Ga_4_L_6_]^12–^ tetrahedral host^6^ has long been known to be entropically driven, due to displacement of ordered solvent molecules from the host cavity.^7−11^ A recent study using terahertz spectroscopy revealed that water molecules encapsulated in the host cavity are highly organized, with a structure resembling amorphous ice.^12^ The encapsulation of a cationic guest molecule within the tetrahedron displaces 8–10 water molecules and desolvates the charged guest, resulting in a large systemic increase in entropy. The magnitude of this entropic gain is sensitive to the solvent conditions and the structure of the guest molecule. The modular nature of these metal–ligand coordination cage hosts enables systematic investigation of structure–activity relationships,^13−16^ with the potential to gain fundamental insights into water organization and solvation entropy.

**Figure 1:**
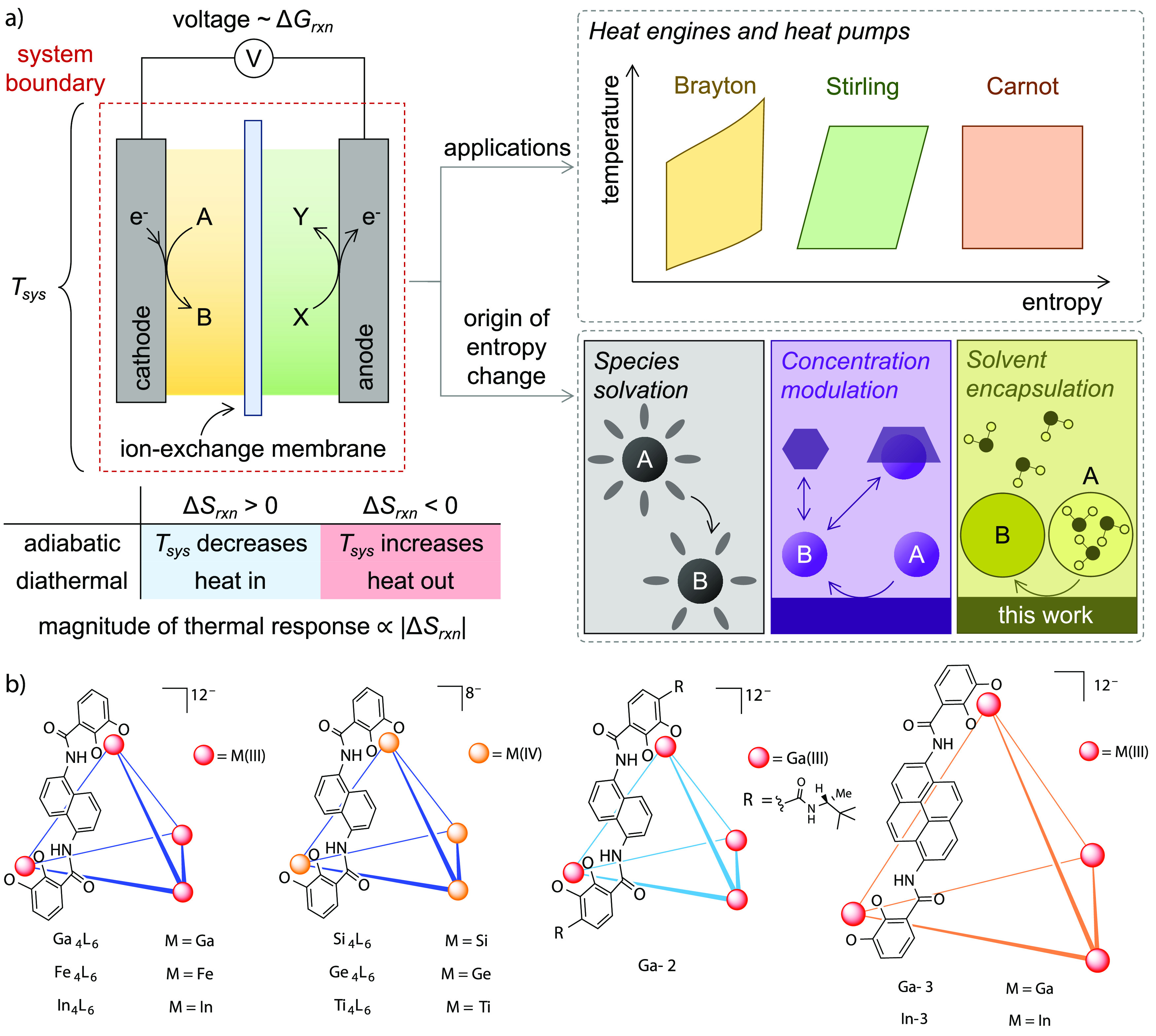
(a) Scheme representing the relationship between thermodynamic parameters and heat-work conversion. (b) Structural diversity of the M_4_L_6_ tetrahedral supramolecular host assembly. The metal centers form the vertices of the tetrahedron, and the ligands bind along the edges. For clarity, only one ligand is shown along the edge of each tetrahedron. Counterions for all hosts are K^+^.

There are few existing ways to rationally tune the reaction entropy despite this property’s great utility. Because of the relationship Δ*G* = Δ*H* – *T*Δ*S*, nonzero reaction entropy forms the basis for conversion between heat (*T*) and free energy (Δ*G*). Numerous applications in energy efficiency and green technologies can be derived from systems with large and reversible reaction entropy. Phase change materials are one example of a field where a rational molecular design approach is taken to enhancing entropy, with uses in energy storage, heat harvesting, and cooling.^17,18^ Thermally regenerative electrochemical cycles (TRECs) utilize a reversible redox entropy change to convert between heat and electricity, presenting a potential application of a solution-state high-entropy supramolecular electrolyte system. Engineers have employed TRECs for electrochemical heat engines and refrigerators.^19,20^ The equilibrium potential of an electrochemical system has a dependence on temperature, provided that the entropy of the system is not zero (the thermogalvanic effect) (Figure 1a). In its simplest application, this relationship between the temperature and electrochemical potential enables temperature sensing. Thermogalvanic engines can also convert low-grade heat into electric current, and conversely, thermogalvanic refrigerators convert electric power to heat absorption, leading to cooling. The efficiency of these conversions is dependent on the Seebeck temperature coefficient (α), which is proportional to the molar entropy (Δ*S*) of the system: a larger Δ*S* of the redox couple leads to a larger temperature change per mole of electrons transferred. These electrochemical heat engines can be used to harvest waste heat from thermal energy generation,^21−23^ and electrochemical refrigerators provide a cooling method that can avoid the use of volatile coolants,^24−26^ which can contribute to atmospheric pollution (hydrofluorocarbons, currently the most common coolant in vapor compression systems, have a global warming potential of up to 2000 times that of CO_2_ when leaked).^27,28^ To improve the efficiency of TRECs, an electrolyte with a large and reversible entropic change is required.^29^ So far, most TRECs have used simple salts as electrolytes, relying on electrochemical changes in solvation entropy, with ferri/ferrocyanide (FCN^4–/3–^) being the most promising choice. Optimization experiments have been conducted,^30,31^ usually involving changes in the counterion, solvent, or concentration of the electrolyte, but these salts are not amenable to synthetic design and modification. Solvation entropy is also bounded by inherent limitations, as described by the Born expression for reaction entropy,^32^ leading to a fundamental bottleneck in this technology.^33^

We hypothesized that an electrochemically controlled guest encapsulation process using the M_4_L_6_ host would result in a system with a large and redox-controlled entropic change, which would be synthetically tunable through the structure of the host and guest. This host–guest system would represent the first of its kind in the TREC literature. The entropic change in the M_4_L_6_ supramolecular system reported herein relies not only on solvent reorganization but also more closely resembles a solvent phase change, resembling materials that undergo phase changes.^17,18,34−38^ The entropy change arises from the large gain in degrees of freedom of the solvent molecules when released from molecular confinement rather than solely from a change in solvent organization around the redox-active species. Deriving the entropic change from solvent ordering through confinement bypasses solvation entropy limitations of nonsupramolecular electrolytes, and we hypothesize that the entropic change of this system would be limited instead by the entropy of the phase transition of the solvent, which is much greater.^39^ Furthermore, the M_4_L_6_ host has proven to be amenable to structural modifications at the metal vertices and of the ligand at both its linker and its end groups (Figure 1b). We envisioned that this synthetic diversification should allow for an unprecedented mechanistic exploration of encapsulation entropy and enable rational synthetic design to optimize the entropy of the system.

## Results and Discussion

**Figure 2:**
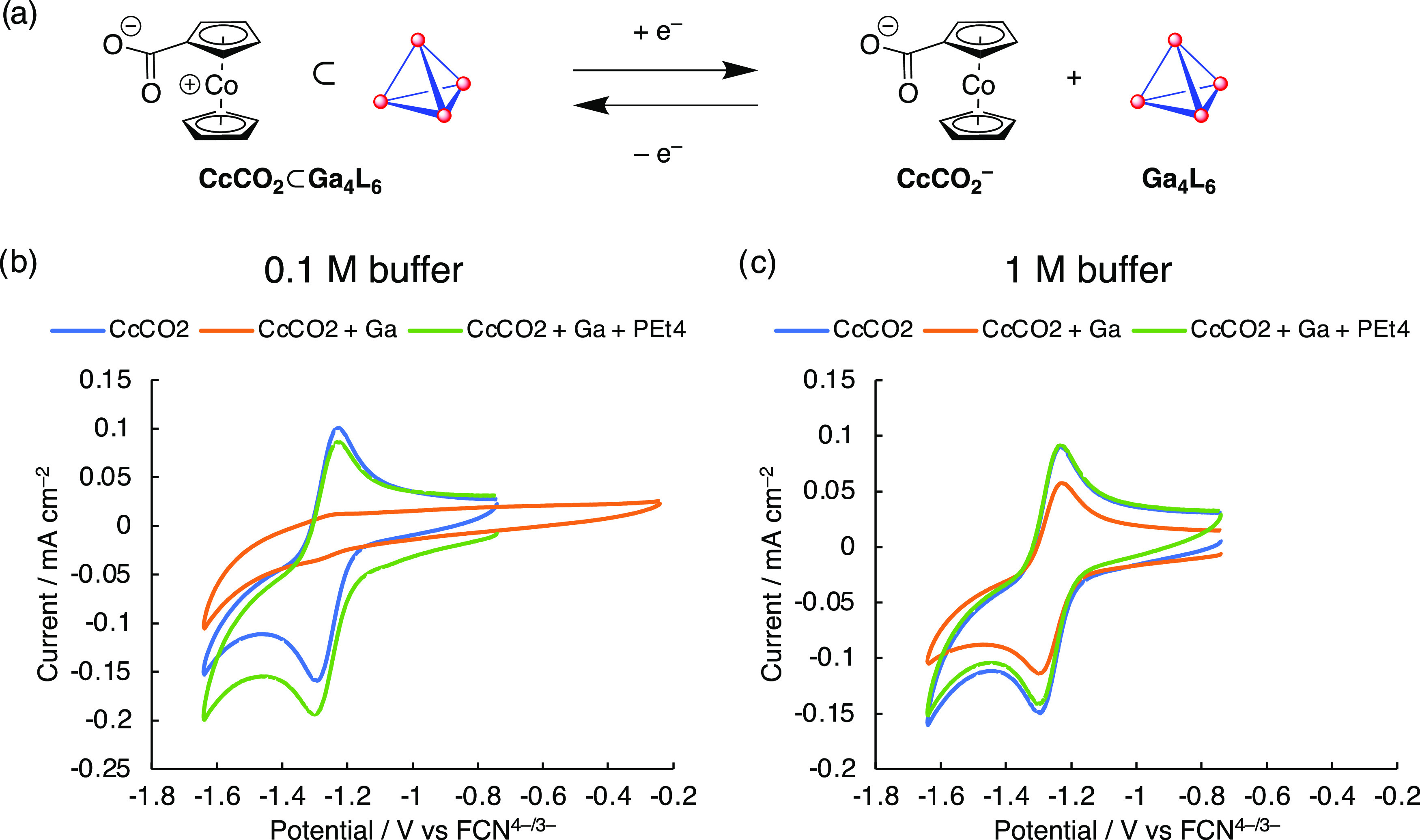
(a) Scheme for the electrochemical behavior of CcCO_2_ in the presence of [Ga_4_L_6_]^12–^. (b) Cyclic voltammogram (CV) of 2 mM CcCO_2_ (blue), 2 mM CcCO_2_ with 2 mM [Ga_4_L_6_]^12–^ (orange), and 2 mM CcCO_2_ with 2 mM [Ga_4_L_6_]^12–^ and 2 mM PEt_4_^+^ (green) in 100 mM pH 12 potassium phosphate buffer in water, 100 mV s^–1^ scan rate, scanning reductively from −0.741 V vs FCN^4–/3–^ for the blue and green traces, and −0.241 V vs FCN^4–/3–^ for the orange trace, 1 cycle. (c) CV of 2 mM CcCO_2_ (blue), 2 mM CcCO_2_ with 2 mM [Ga_4_L_6_]^12–^ (orange) and 2 mM CcCO_2_ with 2 mM [Ga_4_L_6_]^12–^ and 2 mM PEt_4_^+^ (green) in 1 M pH 12 potassium phosphate buffer in water, 100 mV/s scan rate, scanning reductively from −0.741 V vs FCN^4–/3–^, 1 cycle.

Our initial approach drew inspiration from Fujita and co-workers’ electrochemical encapsulation of ferrocene in a metal–organic cationic host.^40^ Previous studies have shown [Ga_4_L_6_]^12–^ to encapsulate cobaltocenium and decamethylcobaltocenium.^41^ Due to the proximity of ferrocene’s redox couple to the oxidation potential of the host’s catecholate ligands,^42^ we switched to cobaltocenium (Cc^+^) to allow more synthetic flexibility. The redox potential of Cc/Cc^+^ is over 1 V more negative than that of the catecholate ligands; therefore, structural modifications do not risk shifting the guest oxidation potential to overlap with that of the host, and the system can operate without nearing the oxidation potential of the host. Hydrocarboxycobaltocenium hexafluorophosphate (CcCO_2_HPF_6_) was selected, due to its improved solubility and slightly less negative reduction potential compared to the parent cobaltocenium (Figure 2a).^43^

In 100 mM potassium phosphate buffer in water at pH = 12, charge-neutral CcCO_2_ displays a reversible redox couple (*E*_1/2_ = −1.262 V vs FCN^4–/3–^) (Figure 2b). When equimolar amounts of CcCO_2_ and [Ga_4_L_6_]^12–^ are combined, however, the redox behavior of CcCO_2_ is seen to be significantly silenced, implying encapsulation. CcCO_2_ (Co(III)) is overall neutrally charged and encapsulates due to hydrophobic effects. Upon addition of an excess of strongly binding guest, tetraethylphosphonium (PEt_4_^+^), which effectively displaces CcCO_2_ from the host cavity (as assayed by ^1^H NMR, Figure S43), the redox behavior of CcCO_2_ returns. These data suggest the redox inhibition of CcCO_2_ by the host.

The Frumkin effect was investigated as an explanation for this behavior. This effect, whereby the charge repulsion between the redox species and the ordering of ions in the double layer hinders electron transfer, has been observed for highly charged species, such as persulfate salts.^44^ Increasing the driving force also increases the repulsion; therefore, the effect cannot be overcome by changing the electrode potential. Modifying conditions that affect the organization of the double layer, such as temperature, electrode material, electrolyte concentration, and solvent, can allow the redox species to reside within the electron transfer distance of the electrode. No change was observed when switching from a glassy carbon working electrode to a platinum working electrode (Figure S9). Increasing the buffer concentration to 1 M, however, the redox behavior of CcCO_2_ can be observed in the presence of the host, although the peak current is smaller (Figure 2c). The diffusion coefficient extracted from the cathodic peak current at varying scan rates of CcCO_2_ in the presence of one equivalent of [Ga_4_L_6_]^12–^ at 1 M buffer concentration is 3.96(3) × 10^–7^ cm^2^ s^–1^, compared to 5.71(4) × 10^–7^ cm^2^ s^–1^ in the absence of the host, implying association of CcCO_2_ with the host, whether internally or externally, and thus moving together as a larger unit.^45^ With the addition of 1.2 equiv of PEt_4_^+^, the diffusion coefficient is 5.09(5) × 10^–7^ cm^2^ s^–1^, implying that the CcCO_2_ is now more freely diffusing, displaced by PEt_4_^+^ from its association with the host.

To determine whether the observed redox peaks in 1 M buffer corresponded to CcCO_2_ inside or outside the host cavity, the experiment was conducted with a guest with an arbitrarily slow exchange rate from the host cavity. Decamethylcobaltocenium (Cp*_2_Co^+^) is too large to exchange from the host cavity (the guest exchange rate is on the order of days),^46^ and [Cp*_2_Co ⊂ Ga_4_L_6_]^11–^ must be synthesized by templation around the Cp*_2_Co^+^ guest. We can thus assume that Cp*_2_Co^+^ resides exclusively within the [Ga_4_L_6_]^12–^ cavity. No redox behavior was observed for [Cp*_2_Co ⊂ Ga_4_L_6_]^11–^ (Figure S10). In contrast, the titanium-based host [Ti_4_L_6_]^8–^ has an accessible Ti(III/IV) redox couple, and its reversible redox behavior has been measured (Figure S11). The four vertices of Ti_4_L_6_ are essentially electronically independent; therefore, four electrons are transferred simultaneously, and the charge of the host changes from −8 to −12. We reasoned that the host itself can access the electrode and undergo electron transfer in the double layer but only prevents electron transfer from the electrode directly to the guest, possibly due to a prohibitively high reorganization energy in the constricted cavity.

Taking these observations together, the observed redox behavior of CcCO_2_ in the presence of [Ga_4_L_6_]^12–^ at 1 M buffer is likely that of CcCO_2_ associated through ion-pairing interactions to the exterior of the host. The overall neutral CcCO_2_ binds to [Ga_4_L_6_]^12–^ through hydrophobic effects in the naphthalene-walled cavity. It is a weakly binding guest and is observed both inside and outside the host cavity, as assessed by ^1^H NMR spectroscopy (Figure S44). In solution, there is an equilibrium between interiorly and exteriorly associated CcCO_2_ to [Ga_4_L_6_]^12–^, and the concentration of CcCO_2_ completely unassociated with the host is relatively low. At a lower ionic strength, the negative charge excess in the electrode is more poorly screened than at the higher 1 M buffer concentration, inhibiting electron transfer to the host–guest complex. As the CcCO_2_ is associated with the host, the redox behavior observed by CV reflects a low concentration of CcCO_2_ within electron transfer distance (Figure 2b). When the buffer concentration is raised to 1 M, the structure of the double layer now allows a higher concentration of the host to reside within electron transfer distance. By association, a higher concentration of CcCO_2_, both interiorly and exteriorly bound to the host, is now also within electron transfer distance to the electrode, as is observed in the increased peak current by CV (Figure 2c).

We then measured the entropic change of this redox-encapsulation process. The Δ*S* of the encapsulation of unsubstituted cobaltocenium was found to be 62 cal mol^–1^ K^–1^ measured using ^1^H NMR (Figure S18). The broadened signals in the NMR of CcCO_2_ preclude measurement of its entropy of encapsulation by NMR, as these signals could not be reliably integrated. Using an electrochemical van’t Hoff technique (see the Supporting Information for Method) enabled measurement of the entropy of the CcCO_2_ Co(II/III) redox couple, as well as the entropy of the host–guest system, including both the encapsulation and redox processes (the scheme shown in Figure 2a). Two vials containing the redox solution were connected via a salt bridge, and a temperature difference was created between them by heating one side while holding the other side at a constant temperature. The difference in open-circuit potential between the solutions at different temperatures was measured and plotted against Δ*T*, and the slope of these points gave the Seebeck temperature coefficient in millivolts per Kelvin. This number could then be converted to Δ*S* in calories per mole per Kelvin through a unit conversion from millivolts to calories per mole.

**Figure 3:**
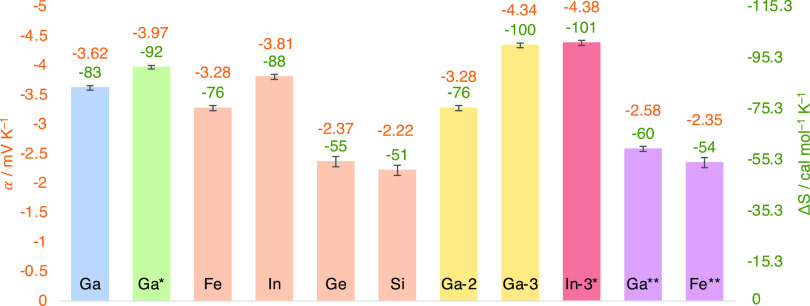
Redox entropy of CcCO_2_ in the presence of various M_4_L_6_ hosts. Orange numbers above the bars correspond to units in mV K^–1^, the axis on the left, and green numbers above the bars correspond to units in cal mol^–1^ K^–1^, the axis on the right. The color of the bars is coded as follows: original system (blue), change in counterion (green), change in metal vertices of host (orange), change in ligand of the host (yellow), change in metal vertices and ligand of the host (red), at saturation concentration (purple). Unless otherwise indicated, measurements were taken with 2 mM CcCO_2_, 2 mM M_4_L_6_, and 1 M pH 12 potassium phosphate buffer. * = measured with sodium phosphate buffer in place of potassium phosphate buffer. ** = measured at saturation concentration of the host: 0.1 M Ga_4_L_6_ or 0.1 M Fe_4_L_6_, respectively, with 0.1 M CcCO_2_. Data for this graph are also provided in Table S22.

The Seebeck temperature coefficient (α) of this system (2 mM [Ga_4_L_6_]^12–^, 2 mM CcCO_2_, 1 M pH 12 potassium phosphate buffer) was found to be −3.62(4) mV K^–1^, which corresponds to a reaction entropy (Δ*S*) of 83.5(9) cal mol^–1^ K^–1^ (in comparison, α is −0.92(1) for potassium ferri/ferrocyanide). Control experiments support that encapsulation in the host is the reason for the increased Δ*S*, as CcCO_2_ alone and CcCO_2_ with the host cavity blocked by a strongly binding guest (PEt_4_^+^) all show significantly lower Δ*S* (−1.87(9) mV K^–1^ or −43(2) cal mol^–1^ K^–1^ and −2.00(4) mV K^–1^ or −46.0(9) cal mol^–1^ K^–1^, respectively). Despite the increase in Δ*S* of the system in the presence of the host, a shift in *E*_1/2_ (proportional to Δ*G*) is not observed in the CV (Figure 2c), although one on the order of ∼200 mV would be expected for a strongly binding guest. Enthalpy–entropy compensation effects have been observed in these host–guest systems.^11^ The absence of a shift in the voltametric peak in Figure 2c upon introduction of [Ga_4_L_6_]^12–^ implies weak host–guest binding (*K*_a_ ∼ 1) and would be consistent with guest ion pairing to the outside of the host cavity. The large entropic value is most likely attributed to displacement of the solvent from within the host cavity, so the guest is likely to reside both inside and outside of the host cavity in equilibrium. This entropic value represents a 4-fold increase versus the state-of-the-art TREC electrolyte, potassium ferricyanide.^29^ Furthermore, synthetic modifications of the host system demonstrated large variations in the entropy (Figure 3). The ligand with the greatest Δ*S* was Ga-**3** (Figure 1), presumably due to the larger cavity size,^47^ which can accommodate more water molecules that are then released upon encapsulation of the guest. The hosts with 8^–^ charge ([Si_4_L_6_]^8–^ and [Ge_4_L_6_]^8–^) showed significantly lower Δ*S*. Switching from potassium phosphate buffer to sodium phosphate buffer also raised Δ*S*, possibly due to the mixture of cations, which has been shown to have an effect on solvent organization.^31^ Taking the conditions and host structural features that gave the highest Δ*S*, we synthesized and measured the Δ*S* of In-**3** in sodium phosphate buffer to be −4.38(6) mV K^–1^ or −101(1) cal mol^–1^ K^–1^.

A few electrolyte systems that bear superficial similarities to the M_4_L_6_ host–guest system have been reported. The iodide/triiodide redox couple has been used as an electrolyte that leverages a change in the degrees of freedom of the redox species (I_3_^–^ ⇋ 3 I^–^), but it does not significantly outperform ferricyanide.^48−50^ A supramolecular system that uses α-cyclodextrin and iodide/triiodide has also been reported,^51^ achieving α = 2.6 mV K^–1^. Another system has been reported in which the ferri/ferrocyanide electrolyte switches between crystalline precipitate and solution state,^52^ achieving up to α = 3.73 mV K^–1^. Though these systems contain supramolecular host–guest chemistry and a phase change, respectively, both of these systems modulate the local concentration of the redox species at the hot and cold electrodes (either through supramolecular encapsulation or precipitation out of solution) and thereby increase the Seebeck coefficient through the relationship between potential and molar quotient described by the Nernst equation. These systems cannot generate power continuously, however, as the electrolyte must be regenerated batchwise, and generating a precipitate also risks passivating the electrode. The M_4_L_6_ system instead derives entropy primarily from the ordering of solvent in confinement. The M_4_L_6_ host–guest system represents the first system to use solvent encapsulation and release to generate an electrochemical entropic change, with the advantage of being entirely solution-state and compatible with continuously run thermogalvanic devices.

**Figure 4:**
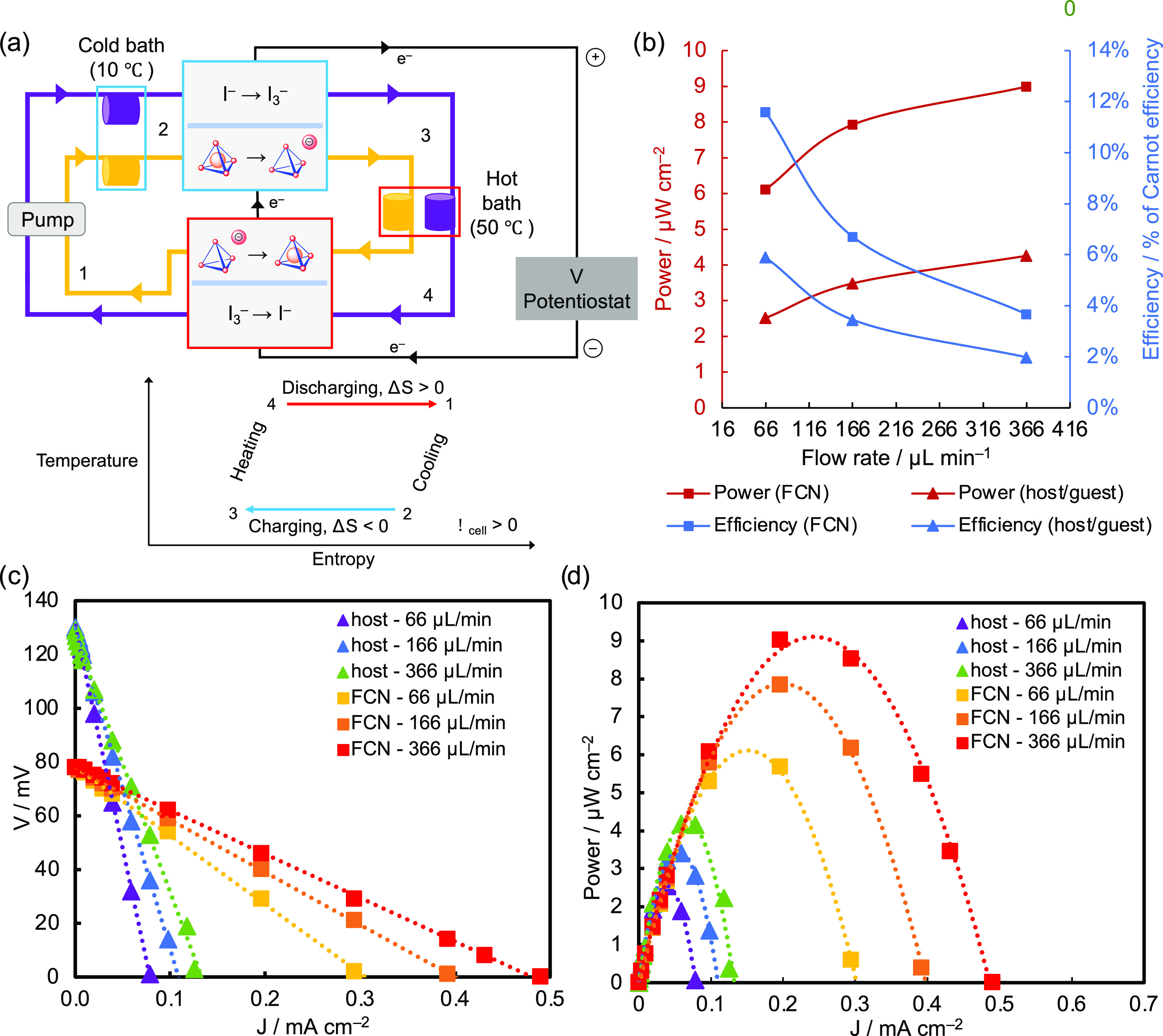
(a) Scheme depicting the construction of the thermogalvanic heat engine. For simplicity, the host–guest redox process is depicted with the guest encapsulated within the host, but reduction of the guest likely occurs outside the host cavity. A temperature vs entropy diagram for the cycle is shown below, with corresponding states 1–4 labeled on the device scheme. (b) Power and efficiency of the thermogalvanic heat engine at different flow rates. Data for this graph are also provided in Table S30. (c) Electrochemical potential at varying current density for the thermogalvanic heat engine at different flow rates. (d) Power at varying current density for the thermogalvanic heat engine at different flow rates.

A simple thermogalvanic heat harvesting engine was constructed and tested with the host–guest system and potassium ferri/ferrocyanide, respectively, both paired with iodide/triiodide, which has a positive Seebeck coefficient (α = 0.5 mV K^–1^) (Figure 4a).^23,24^ Measurements for the host–guest system were performed at pH 12 because the hosts tend to have higher solubility at higher pH. While the solubility of most M_4_L_6_ hosts has been found to be less than 50 mM, the solubility of [Ga_4_L_6_]^12–^ and [Fe_4_L_6_]^12–^ were found to be higher. Because [Fe_4_L_6_]^12–^ is stable to oxygen and higher temperatures, we chose to scale up the [Fe_4_L_6_]^12–^ host–guest system for a direct comparison with the potassium ferri/ferrocyanide system at their respective saturation concentrations (for the host–guest system, measurements were performed with an aqueous solution of 0.1 M [Fe_4_L_6_]^12–^, 0.1 M CcCO_2_HPF_6_, and 1 M pH 12 potassium phosphate buffer; for the ferri/ferrocyanide system, measurements were performed with an aqueous solution of 0.3 M K_3_Fe(CN)_6_ and 0.3 M K_4_Fe(CN)_6_). At saturation, the host–guest system demonstrated a Seebeck temperature coefficient of α = −2.35(8) mV K^–1^ (Δ*S* = −54(2) cal mol^–1^ K^–1^), compared to α = −0.92(1) mV K^–1^ for potassium ferri/ferrocyanide.

The peak power for the potassium ferri/ferrocyanide system is 9 μW cm^–2^ at 4% of Carnot efficiency, matching the reported results.^23^ The host–guest system achieves a higher efficiency value of 6% of Carnot efficiency at a low flow rate, though with a lower power density of 2.5 μW cm^–2^ (Figure 4b). At a higher flow rate, the peak power density rises to 4.3 μW cm^–2^, and the efficiency drops to 2% of Carnot efficiency. This performance demonstrates the feasibility of applying a fundamental property of a supramolecular system (encapsulation entropy) to a functional device. The power density and efficiency of the host–guest system can be improved through further synthetic modifications to improve the solubility of the host. Assuming that current density scales linearly with concentration, the host–guest system can be expected to outperform potassium ferri/ferrocyanide when it achieves a concentration of over approximately 0.2 M, double its present solubility (see the Supporting Information for calculation). In future work and scale-ups of this system, synthetic design can be employed to improve other aspects as well, by replacing cobalt, gallium, and indium, for example, as the mining of these metals is known to be ethically concerning,^53−55^ or by making operating conditions more benign by designing for a system closer to neutral pH to reduce corrosivity. The structural tunability of the supramolecular system engenders the possibility of engineering improvements to its properties through chemical synthesis and demonstrates the potential contribution of an approach to such projects through physical organic chemistry.

## Conclusions

While the M_4_L_6_ tetrahedron has long been known to induce a large entropic increase upon encapsulation of a guest, this feature was until now understood as an interesting property of a supramolecular system. With this work, however, it has been shown that fundamental properties can still find new practical applications. While chemists frequently consider the energy and enthalpy of systems, we less often use molecular design to address entropic properties, and we have fewer strategies to do so. Supramolecular chemists have long performed studies to understand the fundamental properties and rules underlying noncovalent interactions, which contribute to systemic entropy changes. Supramolecular assemblies have the advantage of being adaptable to homogeneous solution-state systems, and the systems reported in this paper leverage the molecular confinement of water to demonstrate some of the largest Seebeck coefficients observed. Therefore, we identify this as a topic of interest in which synthetic chemists, materials scientists, and engineers may find fruitful collaborations. Many aspects of thermogalvanic device development cannot be addressed through chemistry research alone (for example, designing for ease of recycling or degradation at the end of the device’s lifetime or considering the cost of production and modularity of the system, which could preclude adoption of these devices in low-income communities), so we urge collaboration and critical evaluation from adjacent fields, including but not limited to engineering, environmental policy and health, and energy and resources management.

## Supporting Information Available

General methods; synthetic methods (synthesis of all compounds, NMR van’t Hoff method); electrochemical methods (experimental protocols for all electrochemical measurements, including diagrams and photographs of setups); cyclic voltammetry; diffusion coefficients; van’t Hoff plots; device testing data; ^1^H NMR spectroscopy; UV–vis spectroscopy (PDF)
