# Supplementary material for: Tunable Electrochemical Entropy through Solvent Ordering by a Supramolecular Host
Source: J Am Chem Soc. 2023 Nov 13;145(46):25463–70. doi: 10.1021/jacs.3c10145 (PMC10683002; doi:10.1021/jacs.3c10145)
Supplement: Supplementary file 1 — ja3c10145_si_001.pdf [file ja3c10145_si_001.pdf]

Supporting Information

Tunable electrochemical entropy through solvent ordering by a supramolecular host

Kay T. Xia,[†‡] Aravindh Rajan,[+] Yogesh Surendranath,[❖] Robert G. Bergman,[†‡*] Kenneth N. Raymond,[†‡*] F. Dean Toste[†‡*]

Author Affiliations:
[†]Chemical Sciences Division, Lawrence Berkeley National Laboratory, Berkeley, California 94720, United States
[‡]Department of Chemistry, University of California, Berkeley, California 94720, United States
[+]Palo Alto Research Center, 3333 Coyote Hill Road, Palo Alto, California 94304, United States
[❖]Department of Chemistry, Massachusetts Institute of Technology, Cambridge, Massachusetts 02139, United States

Contents

Synthetic Methods 7
Synthesis of Ligand H_4L 7
Synthesis of $K_{12}Ga_4L_6$ 9
Synthesis of $K_{12}In_4L_6$ 10
Synthesis of Fe_4L_6 11
Synthesis of $K_{11}[Cp^*_2Co \subset Ga_4L_6]$ 12
*Synthesis of Ga-**3*** 13
*Synthesis of In-**3*** 14
Synthesis of Ti_4L_6 with Tetraethylammonium 15
Synthesis of Ti_4L_6 with Cobaltocenium 16
Method for Obtaining van't Hoff Plots by NMR 17
Electrochemical Methods 18
Method for Obtaining Diffusion Coefficients 19
Method for Bulk Electrolysis 20
Method for Obtaining van't Hoff Plots Electrochemically 21
Validation of Electrochemical van't Hoff Method 22
Construction and Testing of a Thermogalvanic Heat Engine 23
Other Characterization Methods 35
Method for Determining Solubility 35

Method for Determining Density 35
Cyclic Voltammograms 36
Variable Scan Rate Cyclic Voltammograms 41
Diffusion Coefficients.. 44
van't Hoff Plots 47
Encapsulation of Cobaltocenium in Ga_4L_6 47
Electrochemical van't Hoffs for Redox Reactions 48
Electrochemical van't Hoffs for Redox Encapsulation Reactions 52
Validation of Electrochemical van't Hoff Method.. 64
Device Testing Data 66
Calculation for Power and Efficiency with Increased Solubility..................................... 71
Other Characterization Data 72
Solubility 72
Density 72
Heat Capacity 72
^{1}H NMR Spectra 73
UV-vis Spectra.. 79
References.. 80

List of Figures

Figure S1: Diagram of electrochemical van't Hoff experimental setup 21
Figure S2: Diagram of device assembly 30
Figure S3: Diagram of electrochemical flow cell assembly 31
Figure S4: Diagrams for dimensions of electrolyte diffusers and graphite flow fields 32
Figure S5: Diagrams for cutting ePTFA gaskets 33
Figure S6: Picture of the thermogalvanic device. Insulation removed for clarity. 34
Figure S7: Electrochemical behavior of ferrocene in the presence of Ga_4L_6 36
Figure S8: CV of 2 mM Ferrocene with 2 mM Ga_4L_6 from –1.235 V to 0.765 V vs $FCN^{4-/3-}$ ... 37
Figure S9: CV of 2 mM ferrocene and 2 mM Ga_4L_6 with Pt working electrode 38
Figure S10: CV of 2 mM $K_{11}[Cp^*_2Co\subset Ga_4L_6]$ 39
Figure S11: CV of 2 mM $NEt_4^+\subset Ti_4L_6$ 40
Figure S12: CVs of 2 mM $CcCO_2$ with varying scan rates 41
Figure S13: CVs of 2 mM $CcCO_2$ + 2 mM Ga_4L_6 with varying scan rates 42
Figure S14: CVs of 2 mM $CcCO_2$ + 2 mM Ga_4L_6 + 2.4 mM PEt_4I with varying scan rates 43
Figure S15: Cathodic and anodic peak current densities versus square root of scan rate for oxidation and reduction of $CcCO_2$ 44
Figure S16: Cathodic and anodic peak current densities versus square root of scan rate for oxidation and reduction of $CcCO_2$ with Ga_4L_6 45
Figure S17: Cathodic and anodic peak current densities versus square root of scan rate for oxidation and reduction of $CcCO_2$ with Ga_4L_6 and PEt_4^+ 46
Figure S18: van't Hoff plot of 10 mM cobaltocenium hexafluorophosphate + 10 mM Ga_4L_6 ... 47
Figure S19: van't Hoff plot of 2 mM $CcCO_2$ 48
Figure S20: van't Hoff plot of 2 mM Ga_4L_6 49
Figure S21: van't Hoff plot of 2 mM $CcCO_2$ + 2 mM Ga_4L_6 + 2 mM PEt_4I 50
Figure S22: van't Hoff plot of pH 12 1 mM potassium phosphate buffer (control) 51
Figure S23: van't Hoff plot of 2 mM Ga_4L_6 + 2 mM $CcCO_2$ 52
Figure S24: van't Hoff plot of 2 mM Ga_4L_6 + 2 mM $CcCO_2$ in 1 mM pH 12 sodium phosphate buffer 53
Figure S25: van't Hoff plot of 2 mM Fe_4L_6 + 2 mM $CcCO_2$ 54
Figure S26: van't Hoff plot of 2 mM In_4L_6 + 2 mM $CcCO_2$ 55
Figure S27: van't Hoff plot of 2 mM Ge_4L_6 + 2 mM $CcCO_2$ 56
Figure S28: van't Hoff plot of 2 mM Si_4L_6 + 2 mM $CcCO_2$ 57
Figure S29: van't Hoff plot of 2 mM Ga-**2** + 2 mM $CcCO_2$ 58
Figure S30: van't Hoff plot of 2 mM Ga-**3** + 2 mM $CcCO_2$ 59
Figure S31: van't Hoff plot of 2 mM In-**3** + 2 mM $CcCO_2$ in 1 M pH 12 sodium phosphate buffer 60
Figure S32: van't Hoff plot of 0.1 M Fe_4L_6 + 0.1 M $CcCO_2$ 61
Figure S33: van't Hoff plot of 0.1 M Ga_4L_6 + 0.1 M $CcCO_2$ 62
Figure S34: Open circuit potential versus log Q for 2 mM $CcCO_2$ 64
Figure S35: Open circuit potential versus log Q for 2 mM $CcCO_2$ + 2 mM Ga_4L_6 65
Figure S36: Potential vs Time for Host-Guest System at 366 µL min^{-1} flow rate 66
Figure S37: Potential vs Time for Host-Guest System at 166 µL min^{-1} flow rate 67
Figure S38: Potential vs Time for Host-Guest System at 66 µL min^{-1} flow rate 68
Figure S39: ^{1}H NMR spectrum of Ga-**3** 73

Figure S40: ^{1}H NMR spectrum of In-**3** 74
Figure S41: ^{1}H NMR spectrum of $NEt_4^+ \subset Ti_4L_6$ 75
Figure S42: ^{1}H NMR spectrum of $Cc^+ \subset Ti_4L_6$ 76
Figure S43: ^{1}H NMR spectrum of Ga_4L_6 and $CcCO_2HPF_6$ and PEt_4I 77
Figure S44: ^{1}H NMR spectrum of Ga_4L_6 and $CcCO_2HPF_6$ 78
Figure S45: UV-vis spectrum of Fe_4L_6 79

List of Tables

Table S1: Incremental addition volumes 22
Table S2: Bill of materials used for the thermogalvanic device 25
Table S3: Diffusion coefficient of $CcCO_2$ 44
Table S4: Diffusion coefficient of $CcCO_2$ with Ga_4L_6 45
Table S5: Diffusion coefficient of $CcCO_2$ with Ga_4L_6 and PEt_4^+ 46
Table S6: Data for van't Hoff plot of 10 mM cobaltocenium hexafluorophosphate + 10 mM Ga_4L_6 47
Table S7: Data for van't Hoff plot of 2 mM $CcCO_2$ 48
Table S8: Data for van't Hoff plot of 2 mM Ga_4L_6 49
Table S9: Data for van't Hoff plot of 2 mM $CcCO_2$ + 2 mM Ga_4L_6 + 2 mM PEt_4I 50
Table S10: Data for van't Hoff plot of pH 12 1 mM potassium phosphate buffer (control) 51
Table S11: Data for van't Hoff plot of 2 mM Ga_4L_6 + 2 mM $CcCO_2$ 52
Table S12: Data for van't Hoff plot of 2 mM Ga_4L_6 + 2 mM $CcCO_2$ in 1 mM pH 12 sodium phosphate buffer 53
Table S13: Data for van't Hoff plot of 2 mM Fe_4L_6 + 2 mM $CcCO_2$ 54
Table S14: Data for van't Hoff plot of 2 mM In_4L_6 + 2 mM $CcCO_2$ 55
Table S15: Data for van't Hoff plot of 2 mM Ge_4L_6 + 2 mM $CcCO_2$ 56
Table S16: Data for van't Hoff plot of 2 mM Si_4L_6 + 2 mM $CcCO_2$ 57
Table S17: Data for van't Hoff plot of 2 mM Ga-**2** + 2 mM $CcCO_2$ 58
Table S18: Data for van't Hoff plot of 2 mM Ga-**3** + 2 mM $CcCO_2$ 59
Table S19: Data for van't Hoff plot of 2 mM In-**3** + 2 mM $CcCO_2$ in 1 M pH 12 sodium phosphate buffer 60
Table S20: Data for van't Hoff plot of 0.1 M Fe_4L_6 + 0.1 M $CcCO_2$ 61
Table S21: Data for van't Hoff plot of 0.1 M Ga_4L_6 + 0.1 M $CcCO_2$ 62
Table S22: Summary of Electrochemical van't Hoff Data 63
Table S23: Data for Figure S28 64
Table S24: Data for Figure S28 65
Table S25: Current steps for experiment in Figure S31 66
Table S26: Current steps for experiment in Figure S32 67
Table S27: Current steps for experiment in Figure S33 68
Table S28: Summary of Data for Host-Guest System 69
Table S29: Summary of Data for Ferri/ferrocyanide System 69
Table S30: Comparison of Power and Efficiency 70
Table S31: Comparison of Figures of Merit 70
Table S32: Solubility of various compounds 72
Table S33: Density of electrolyte solutions 72
Table S34: Heat Capacity of electrolyte solutions 72

General Methods

Unless otherwise noted, all reactions were carried out in oven-dried glassware sealed with rubber septa under a nitrogen atmosphere with Teflon-coated magnetic stir bars. Deuterated solvents were purchased from Cambridge Isotope Laboratories. Reagents were purchased from commercial sources and used without further purification unless otherwise noted. Dimethylformamide (DMF), acetonitrile (CH_3CN), and tetrahydrofuran (THF) were dried and purified using a solvent purification system (JC Meyer) under argon. Thin-layer chromatography (TLC) analysis of reaction mixtures were performed using Merck silica gel 60 F254 TLC plates and visualized using $KMnO_4$ stain or UV. Column chromatography and preparative TLC was carried out using Merck Silica Gel 60 Å, 230 X 400 mesh or Merck silica gel 60 F254 TLC plates, respectively. Proton nuclear magnetic resonance (^1H NMR) spectra were taken with AV-300, AVB-400, AVQ-400, AV-500, or AV-600 Bruker spectrometers operating at 300MHz, 400MHz, 500 MHz, or 600 MHz. Chemical shifts are reported in parts per million (ppm) with reference to the appropriate residual solvent signal. ^1H NMR: $CDCl_3$ (δ: 7.26 ppm), DMSO-d_6 (δ: 2.50 ppm), MeOD (δ: 3.31 ppm), D_2O (δ: 4.79 ppm).[1] ^1H NMR multiplicities are reported as follows: s (singlet), d (doublet), t (triplet), q (quartet), sept (septet), m (multiplet).

Synthetic Methods

Ga_4L_6, In_4L_6, Si_4L_6, Ge_4L_6, were synthesized according to a previously reported procedure.[2] Fe_4L_6 was synthesized according to a previously reported procedure.[3] $K_9(Cp^*{}_2Co)_2[Cp^*{}_2Co \subset Ga_4L_6]$ was synthesized according to a previously reported procedure.[4] Ga-**2** was synthesized according to a previously reported procedure.[5] Cobaltocenium carboxylic acid ($CcCO_2HPF_6$) was synthesized according to a reported procedure.[6] Ligand H_4**3** was synthesized according to a reported procedure.[7] Procedures for which the scale was modified are reported below.

Synthesis of Ligand H_4L

2,3-Dimethoxybenzoic acid (20 g, 110 mmol) and 20 mL dichloromethane were added to a 100-mL round bottom flask equipped with a stir bar and stirred under N_2. Thionyl chloride (16 mL, 220 mmol) was added by syringe. The yellow solution was stirred for 10 minutes after which three drops of dimethylformamide were added by syringe. The solution was stirred for three hours after which remaining thionyl chloride was removed via rotary evaporation. Potassium hydroxide pellets were added to the solvent collection flask of the rotary evaporator, and after solvent removal, the solids in the flask were redissolved in dichloromethane and solvent removal by rotary evaporation was repeated three times to remove residual thionyl chloride. 2,3-Dimethoxybenzoyl chloride was obtained as an off white solid and was used in the next step without further purification.

2,3-Dimethoxybenzoyl chloride was dissolved in dry dichloromethane (40 mL) and transferred to a 250-mL round bottom flask equipped with a stir bar. 1,5 diaminonaphthalene (2.10 g, 13.2 mmol) and dry dichloromethane (60 mL) were added to the flask. The resulting solution was placed in an ice bath and stirred under N_2. Dry triethylamine (15.3 mL, 110 mmol) was added to the solution via syringe, and the reaction was stirred overnight. The solution was washed twice with 1 M HCl (200 mL) followed by two washes with 1 M NaOH (200 mL). The organic layer was dried over $NaSO_4$ and concentrated *in vacuo* to yield a brown solid. This solid was recrystallized in dichloromethane to yield ligand **Me_4L** as white crystals. ^1H NMR (400 MHz, CDCl3) δ 10.74 (s, 2H), 8.57 (d, J = 7.6 Hz, 2H), 7.90 (ddd, J = 8.5, 7.9, 1.5 Hz, 4H), 7.60 (dd, J = 8.1, 7.9 Hz, 2H), 7.26 (dd, J = 8.0, 6.5 Hz, 2H), 7.16 (dd, J = 8.1, 1.5 Hz, 2H), 4.14 (s, 6H), 4.03 (s, 6H).

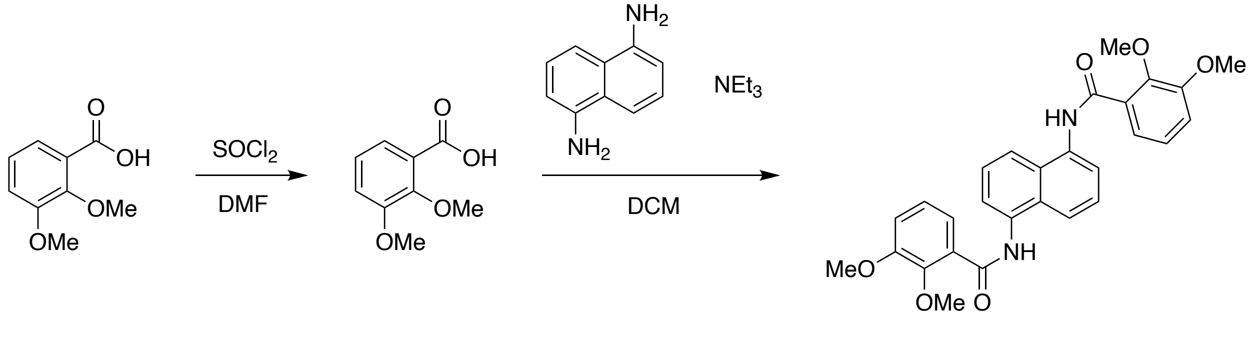

Ligand **Me_4L** (12 g, 25 mmol) was added to a 500-mL round bottom flask equipped with a stir bar and dissolved in dichloromethane (300 mL). The solution was cooled to -78 °C and neat BBr_3 (51g, 20 mL, 206 mmol) was added *via* syringe under an atmosphere of N_2. The yellow mixture was allowed to warm to room temperature and was stirred overnight. The solution was

then carefully poured into a beaker of ice, and the solution was stirred while it warmed to room temperature. The white-grey solid was then filtered and washed with water. The solid was transferred to a 500-mL round bottom flask and suspended in 300 mL of water and refluxed for 12 hours. The resulting white solid was collected by filtration, and dried *in vacuo* to yield ligand **H_4L** as a white powder. ^{1}H NMR (400 MHz, DMSO-*d6*) δ 11.94 (s, 2H), 10.92 (s, 2H), 9.53 (s, 2H), 7.90 (m, 4H), 7.63 (m, 4H), 7.03 (d, 2H), 6.84 (m, 2H).

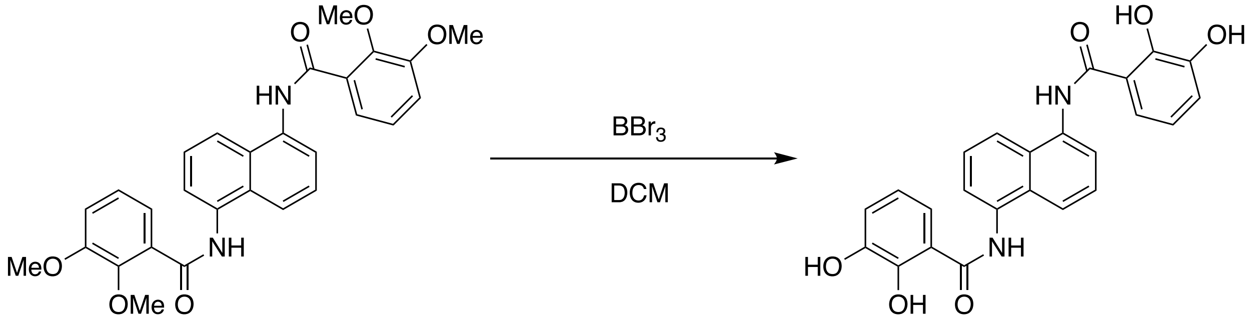

Synthesis of $K_{12}Ga_4L_6$

In a 500 mL three-neck round bottom flask equipped with a 500 mL addition funnel, ligand **H_4L** (5 g, 11.6 mmol, 6 equiv.) and $Ga(acac)_3$ (2.84 g, 7.7 mmol, 4 equiv.) were combined in degassed MeOH (200 mL). This suspension was further sparged with N_2 for 20 minutes. In the meantime, ether (200 mL) was added to the addition funnel and was sparged with N_2 for 30 minutes. KOH (1.95 g, 34.8 mmol, 18 equiv.) was added dropwise as a 1M solution in degassed MeOH. The milky white solution became homogeneous upon addition of base, and the resulting yellow solution was stirred under N_2 for thirty minutes. Ether was then added dropwise via the addition funnel. Upon the first signs of precipitation, the addition was halted, and the solution was stirred for an additional two hours to allow the slow precipitation of $K_{12}Ga_4L_6$. $K_{12}Ga_4L_6$ was isolated by filtration as a pale-yellow solid, dried briefly under vacuum, and immediately transferred to an air free glovebox. ^{1}H NMR (500 MHz, Methanol-*d*4) δ 8.05 (d, *J* = 7.8 Hz, 12H), 7.77 (d, *J* = 8.6 Hz, 12H), 7.25 (d, *J* = 8.2 Hz, 12H), 6.96 (t, *J* = 8.2 Hz, 12H), 6.66 (dd, *J* = 7.3, 1.6 Hz, 12H), 6.37 (t, *J* = 7.8 Hz, 12H).

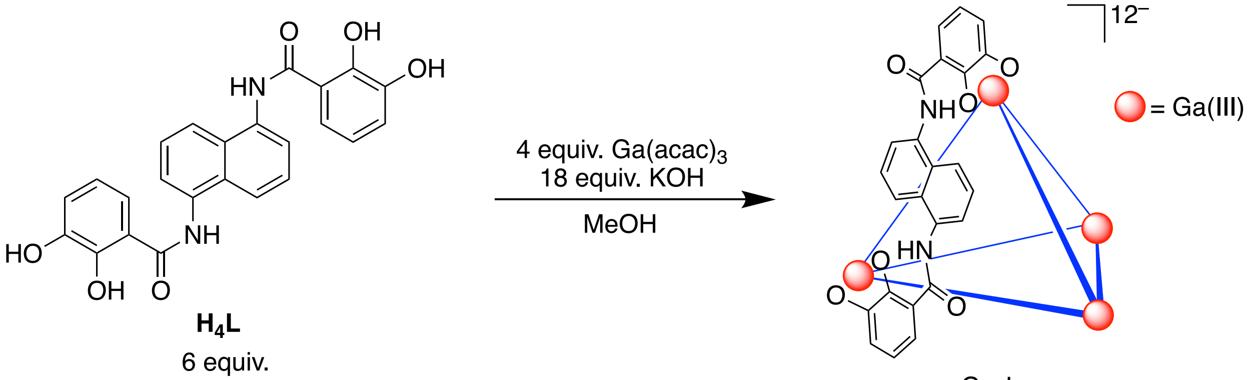

H_4L

6 equiv.

Ga_4L_6

Synthesis of $K_{12}In_4L_6$

In an air-free glove box, ligand **H_4L** (300 mg, 0.70 mmol, 6 equiv.), NMe_4Br (215 mg, 1.40 mmol, 12 equiv.), and KOH (120 mg, 2.14 mmol, 18 equiv.) were added to a 100 mL round bottom flask and then stirred at 720 rpm in MeOH (50 mL) until homogenous. To this solution, $In(acac)_3$ (195 mg, 0.47 mmol, 4 equiv.) was added, and the mixture was stirred at room temperature overnight, upon which a precipitate formed. The host-guest complex $NMe_4^+ \subset In_4L_6$ was isolated by filtration as a pale-yellow solid.

In an air-free glovebox, $NMe_4^+ \subset In_4L_6$ was added as a suspension in a saturated solution of KPF_6 in acetone (200 mL) and MeOH (5 mL) in a 250 mL round bottom flask. This mixture was stirred overnight at 720 rpm at room temperature. The solids were collected by filtration and washed with acetone (20 mL). The guest exchange procedure was repeated three times to generate $K_{12}In_4L_6$ with <5% encapsulated NMe_4^+. ^1H NMR (400 MHz, DMSO-d_6) δ 13.56 (s, 1H), 8.04 (d, J = 7.7 Hz, 1H), 7.60 (d, J = 8.7 Hz, 1H), 7.06 (d, J = 7.9 Hz, 1H), 6.91 (t, J = 8.0 Hz, 1H), 6.37 (d, J = 6.6 Hz, 1H), 6.18 (t, J = 7.7 Hz, 1H).

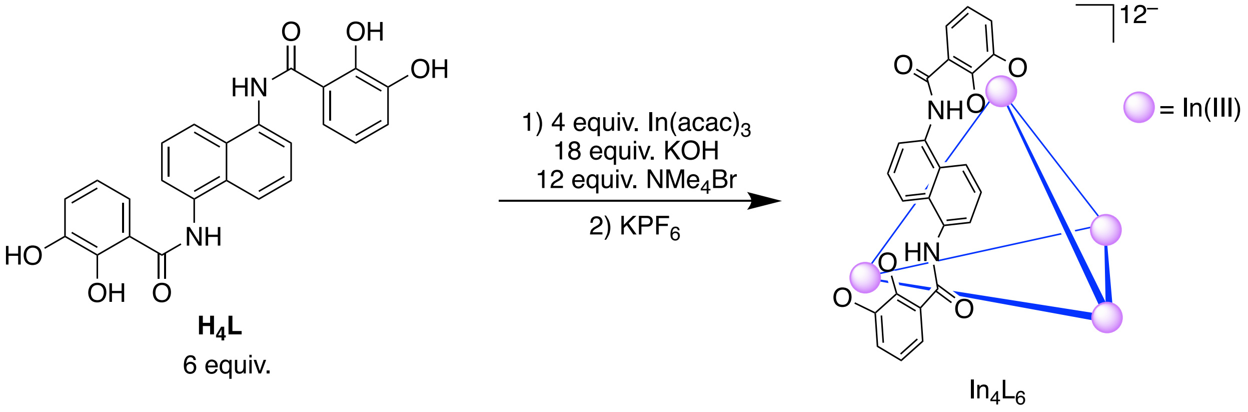

Synthesis of Fe_4L_6

In a 500 mL three-neck round bottom flask equipped with a 500 mL addition funnel, ligand **H_4L** (3 g, 7.0 mmol, 6 equiv.) and $Fe(acac)_3$ (1.64 g, 4.6 mmol, 4 equiv.) were combined in degassed MeOH (120 mL). This suspension was further sparged with N_2 for 20 minutes. In the meantime, ether (150 mL) was added to the addition funnel and was sparged with N_2 for 30 minutes. KOH (1.17 g, 20.9 mmol, 18 equiv.) was added dropwise as a 1M solution in degassed MeOH. The solution became homogeneous upon addition of base, and the resulting dark reddish-purple solution was stirred under N_2 for thirty minutes. Ether was then added dropwise via the addition funnel. Upon the first signs of precipitation, the addition was halted, and the solution was stirred for an additional two hours to allow the slow precipitation of $K_{12}Fe_4L_6$. $K_{12}Fe_4L_6$ was isolated by filtration as a dark red solid and dried under vacuum. Characterization by UV-visible spectroscopy is shown in **Figure S45**.

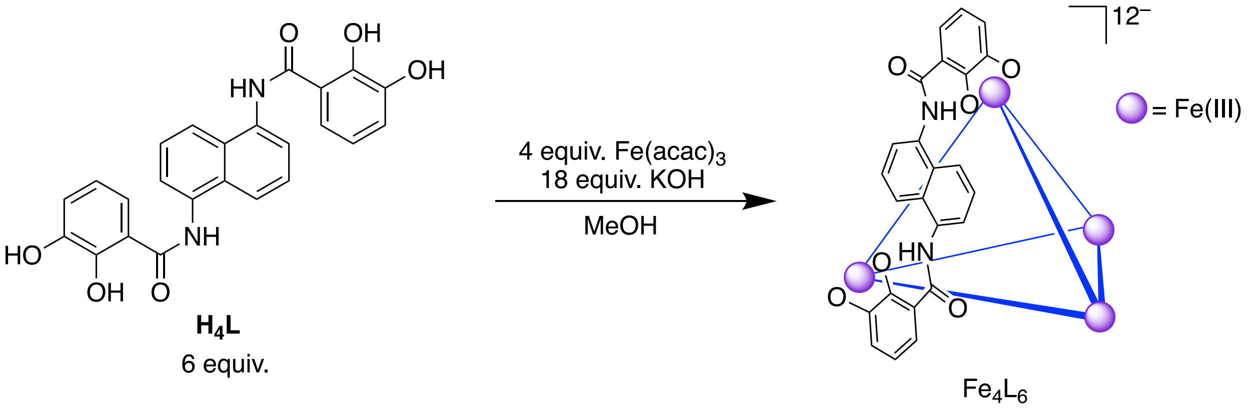

Synthesis of $K_{11}[Cp^*_2Co\subset Ga_4L_6]$

$K_9(Cp^*_2Co)_2[Cp^*_2Co\subset Ga_4L_6]$ was synthesized according to a reported procedure.[4] To remove the two exterior $Cp^*_2Co^+$ compounds, a guest exchange procedure was performed. In an air-free glovebox, 50 mg $K_9(Cp^*_2Co)_2[Cp^*_2Co\subset Ga_4L_6]$ was added as a suspension in a saturated solution of KPF_6 in acetone (20 mL) with a few drops of MeOH in a 20 mL scintillation vial. This mixture was stirred overnight at room temperature to exchange the NMe_4^+ guest for K^+. The solids were collected by filtration and washed with acetone (5 mL). This procedure was repeated four times to generate $K_{11}[Cp^*_2Co\subset Ga_4L_6]$ with <10% exterior $Cp^*_2Co^+$. ^1H NMR (500 MHz, DMSO-d_6): δ 13.32 (s, 4H, NH), 12.85 (s, 4H, NH), 12.83 (s, 4H, NH), 8.28 (overlapping m, 8H, *aryl*), 7.81 (d, J = 7.5 Hz, 4H, *aryl*), 7.40 (d, J = 7.5 Hz, 4H, *aryl*), 7.30 (d, J = 7.5 Hz, 4H, *aryl*), 7.19 (d, J = 7.5 Hz, 4H, *aryl*), 7.12 (overlapping d, J = 8.0 Hz, 8H, *aryl*), 6.76 (d, J = 7.5 Hz, 4H, *aryl*), 6.66 (overlapping m, 8H, *aryl*), 6.29 (d, J = 7.0 Hz, 4H, *aryl*), 6.21 (overlapping m, 4H, *aryl*), 6.18 (t, J = 7.0 Hz, 4H, *aryl*), 1.58 (s, <3H, exterior $Cp^*_2Co^+$), −0.72 (s, 30H, encapsulated $Cp^*_2Co^+$).

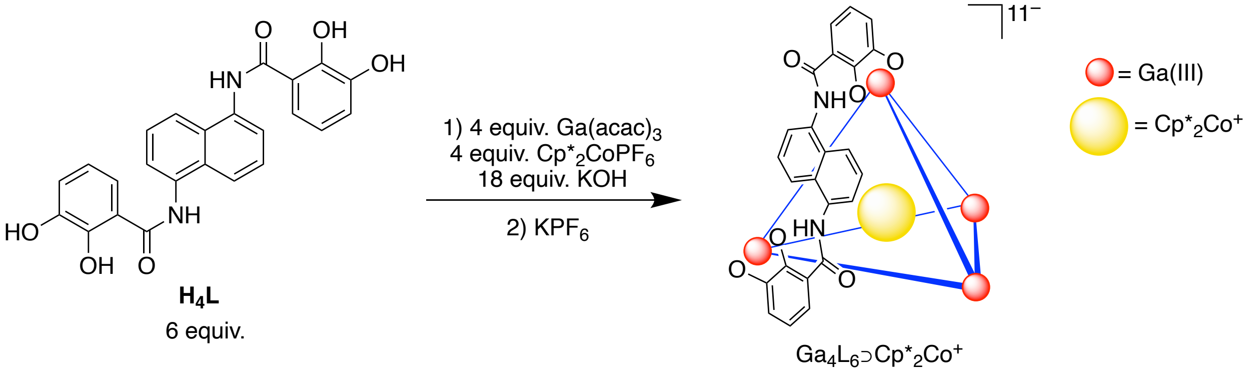

<u>*Synthesis of Ga-3*</u>

Assembly Ga-**3** ($K_{12}Ga_4\mathbf{3}_6$) was synthesized according to a modified procedure.[7] In a 20 mL scintillation vial, ligand H_4**3** (100 mg, 0.197 mmol, 6 equiv.) and $Ga(acac)_3$ (48 mg, 0.132 mmol, 4 equiv.) were combined in degassed MeOH (5 mL). KOH (33 mg, 0.592 mmol, 18 equiv.) was added dropwise as a 1M solution in degassed MeOH. The solution became homogeneous upon addition of base, and the dark yellow solution was stirred under N_2 for three days. The solution was then concentrated under reduced pressure to 1 mL. A minimal amount of acetone was added until a yellow precipitate formed. The precipitate was isolated by centrifugation, and then redissolved in a minimal amount of methanol. Addition of acetone to form precipitate, followed by isolation of the solid through centrifugation was repeated 3 more times to purify the product. Ga-**3** was isolated as a bright yellow powder. ^{1}H NMR (500 MHz, MeOD) δ 8.99 (d, J = 8.5 Hz, 1H), 8.44 (d, J = 9.3 Hz, 1H), 7.74 (d, J = 9.4 Hz, 1H), 7.37 (dd, J = 8.2, 1.7 Hz, 1H), 6.72 (dd, J = 7.3, 1.7 Hz, 1H), 6.41 (t, J = 7.8 Hz, 1H).

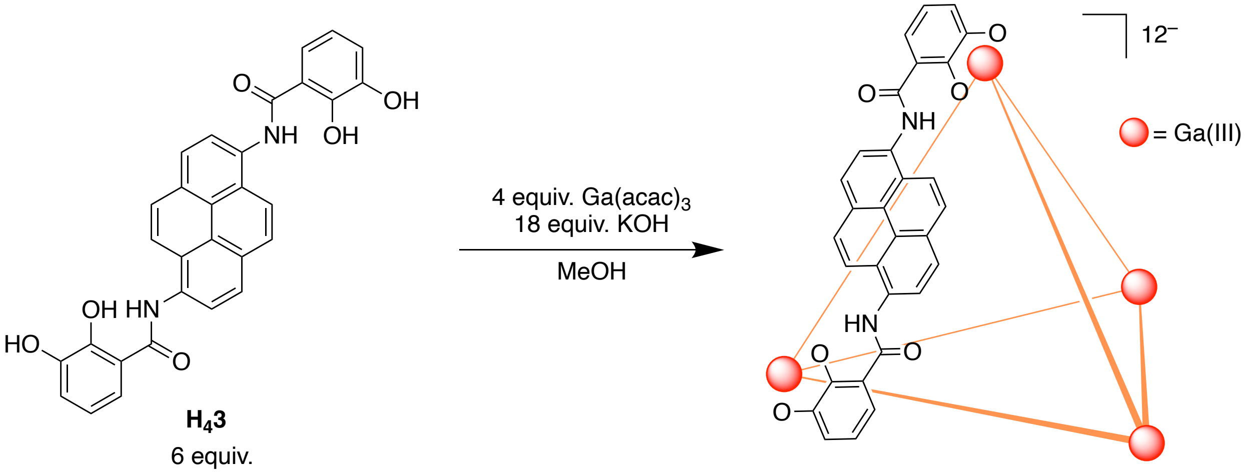

$H_4$3
6 equiv.

Ga-**3**

Synthesis of In-3

Assembly In-**3** ($K_{12}In_4\mathbf{3}_6$) was synthesized according to a modified procedure.[7] In an air-free glove box, ligand $H_4\mathbf{3}$ (72 mg, 0.14 mmol, 6 equiv.), NMe_4Br (43 mg, 0.28 mmol, 12 equiv.), and KOH (24 mg, 0.43 mmol, 18 equiv.) were added to a 20 mL scintillation vial and dissolved in MeOH (10 mL). When homogenous, this solution was added to another 20 mL scintillation vial containing $In(acac)_3$ (39 mg, 0.095 mmol, 4 equiv.), and the mixture was stirred at room temperature for three days. Acetone was added until a precipitate formed. The host-guest complex $NMe_4^+ \subset$In-**3** was isolated by filtration as a dark orange solid. In an air-free glovebox, $NMe_4^+ \subset$In-**3** was added as a suspension in a saturated solution of KPF_6 in acetone (80 mL) with a few drops of MeOH in a 20 mL scintillation vial. This mixture was stirred overnight at room temperature to exchange the NMe_4^+ guest for K^+. The solids were collected by filtration and washed with acetone (5 mL). This procedure was repeated twice to generate In-**3** with <10% encapsulated NMe_4^+. ^1H NMR (400 MHz, DMSO) δ 8.99 (d, J = 8.5 Hz, 12H), 8.38 (d, J = 9.2 Hz, 12H), 7.79 (s, 12H), 7.41 (d, J = 8.6 Hz, 12H), 7.11 (d, J = 8.1 Hz, 12H), 6.42 (d, J = 7.0 Hz, 12H), 6.22 (t, J = 7.8 Hz, 12H).

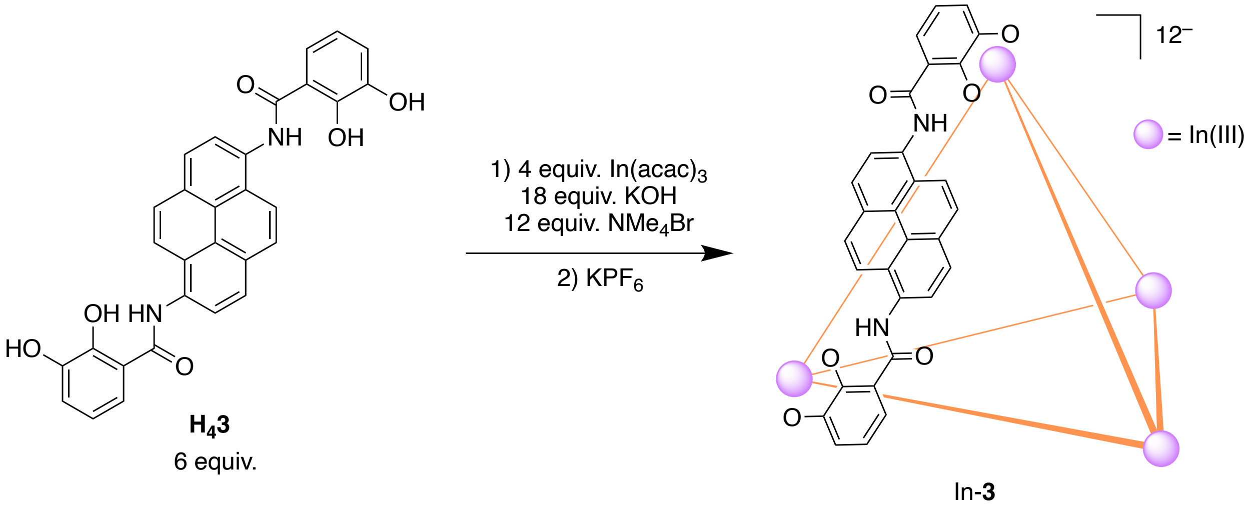

Synthesis of Ti_4L_6 with Tetraethylammonium

Assembly $NEt_4^+ \subset Ti_4L_6$ ($K_{11}Ti_4L_6NEt_4^+$) was synthesized according to a modified procedure. To a 100 mL three-necked round bottom flask equipped with a reflux condenser was added ligand **H_4L** (500 mg, 1.16 mmol, 6 equiv.), NEt_4Cl (96 mg, 0.58 mmol, 3 equiv.), and anhydrous DMF (24 mL) under N_2. Titanium(IV) isopropoxide (230 µL, 0.774 mmol, 4 equiv.) was added to the reaction mixture. The resulting homogeneous solution was stirred at 130 °C for 18 h, then allowed to cool to ambient temperature and treated with excess potassium bicarbonate (300 mg) as an aqueous solution (1 mL). After stirring the resulting mixture for 15 minutes, the resulting precipitate was removed by filtration. The filtrate was treated with diethyl ether (150 mL) and THF (20 mL) to generate an orange precipitate ($NEt_4^+ \subset Ti_4L_6$), which was collected by filtration. To a 250 mL round bottom flask was added $NEt_4^+ \subset Ti_4L_6$ as a suspension in a saturated solution of KPF_6 in acetone (200 mL) to remove excess NEt_4^+. This mixture was stirred for 16 h at room temperature, after which the orange solid was collected by filtration. ^1H NMR (400 MHz, DMSO) δ 11.84 (s, 1H), 8.20 (d, J = 7.9 Hz, 1H), 7.71 (d, J = 8.5 Hz, 1H), 7.42 (d, J = 7.9 Hz, 1H), 7.17 (d, J = 8.2 Hz, 1H), 6.60 (t, J = 7.9 Hz, 1H), 6.38 (d, J = 7.5 Hz, 1H), 3.40 (q, J = 7.0 Hz, 8H, exterior NEt_4^+), 1.11 (t, J = 7.0 Hz, 12H, exterior NEt_4^+), –1.00 (tt, J = 8.1, 3.8 Hz, 8H, interior NEt_4^+), –1.86 (tt, J = 7.4 Hz, 1.8 Hz, 12H, interior NEt_4^+).

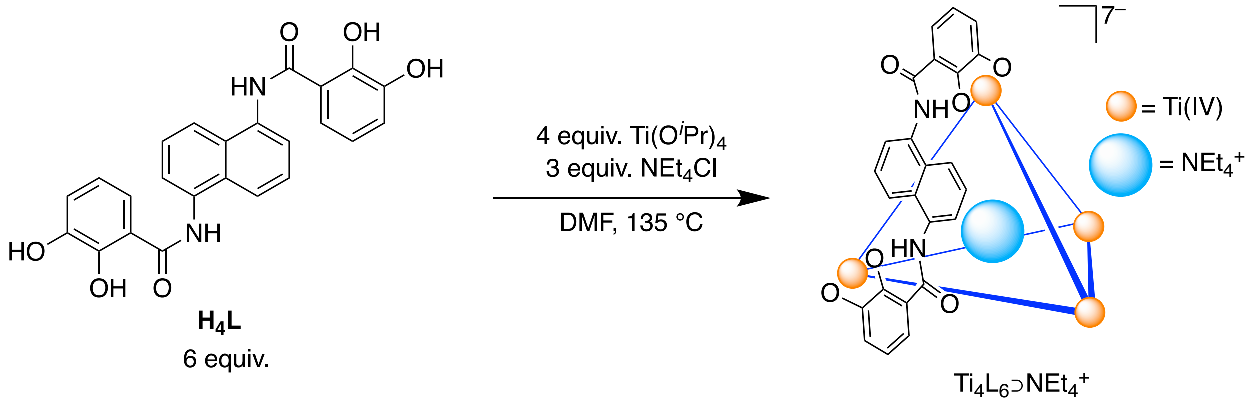

Synthesis of Ti_4L_6 with Cobaltocenium

Assembly $Cc^+ \subset Ti_4L_6$ [$K_8Ti_4L_6(Cc^+)_4$] was synthesized according to a modified procedure. To a 100 mL three-necked round bottom flask equipped with a reflux condenser was added ligand **H_4L** (250 mg, 0.58 mmol, 6 equiv.), cobaltocenium hexafluorophosphate ($CcPF_6$) (508 mg, 1.45 mmol, 15 equiv.), and anhydrous DMF (12 mL) under N_2. Titanium(IV) isopropoxide (115 µL, 0.774 mmol, 4 equiv.) was added to the reaction mixture. The resulting homogeneous solution was stirred at 130 °C for 4 days, then allowed to cool to ambient temperature and treated with excess potassium bicarbonate (300 mg) as an aqueous solution (1 mL). After stirring the resulting mixture for 15 minutes, the resulting precipitate was removed by filtration. The filtrate was treated with diethyl ether (150 mL) and THF (20 mL) to generate an orange precipitate ($Cc^+ \subset Ti_4L_6$), which was collected by filtration.

To a 250 mL round bottom flask was added $Cc^+ \subset Ti_4L_6$ as a suspension in a saturated solution of KPF_6 in acetone (200 mL) to remove excess Cc^+. This mixture was stirred for 16 h at room temperature, after which the solid was collected by filtration. ^1H NMR (400 MHz, DMSO) δ 11.82 (s, 12H), 8.18 (d, J = 7.9 Hz, 12H), 7.69 (d, J = 8.5 Hz, 12H), 7.40 (d, J = 7.9 Hz, 12H), 7.15 (d, J = 8.2 Hz, 12H), 6.58 (t, J = 7.9 Hz, 12H), 6.36 (d, J = 7.5 Hz, 12H), 5.59 (s, 10H, external Cc^+), 1.95 (s, 10H, internal Cc^+).

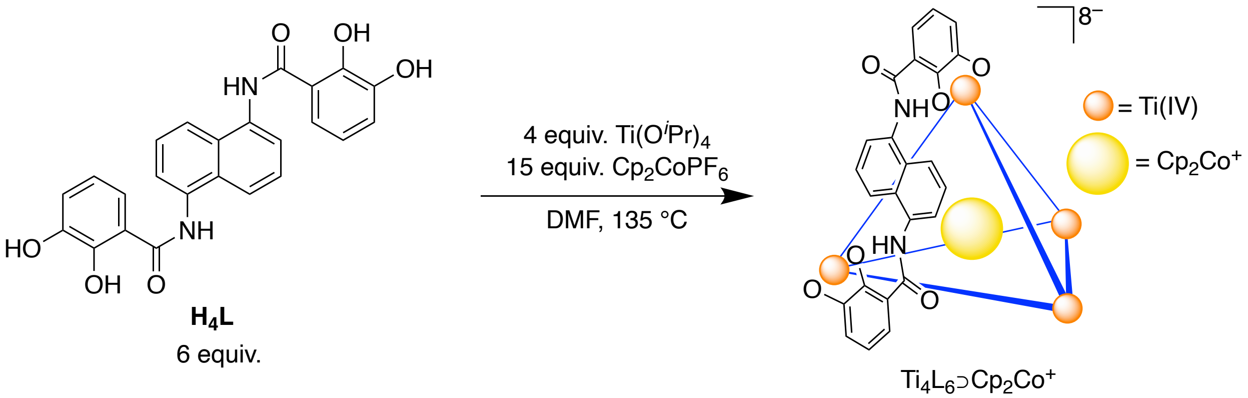

The Ti_4L_6 host cannot be synthesized in the absence of a strongly binding template. The guest exchange procedure from these strongly binding guests is significantly more challenging than the procedure for replacing NMe_4^+ with K^+. As a result, Ti_4L_6 was not included in the series of van't Hoff measurements.

Method for Obtaining van't Hoff Plots by NMR

Stock solutions of the appropriate encapsulated species (10 mM cobaltocenium and 10 mM Ga_4L_6) were prepared in D_2O buffered with 100 mM potassium phosphate at pD = 8.0) at room temperature. The pD of the solution was not corrected at different temperatures. Dioxane (2 μL) was added as an internal standard. The reaction was monitored immediately by 1H NMR at a temperature of 25 °C. The concentration of the encapsulated guest was monitored using 8 scans with a delay time of 4 seconds and a 90° pulse of 13.5 μsec for each time point. Data points were taken at increasing temperature intervals of 10 °C, allowing the sample to equilibrate for 15 min at each temperature until no further change in guest concentration was observed. The thermodynamic data were plotted on a van't Hoff graph with the observed equilibria constants vs. 1/T and the data was fit using a linear least squares regression.

Electrochemical Methods

Electrochemical experiments were conducted using a CHI 760E potentiostat, a leakless Ag/Ag^+ reference electrode (eDAQ ET069, $FCN^{4-/3-}$ at 0.241 V vs Ag/Ag^+ in 100 mM potassium phosphate buffer at pH = 12, and $FCN^{4-/3-}$ at 0.235 V vs Ag/Ag^+ in 100 mM KPF_6 in DMF), a high surface area Pt-mesh counter electrode (Alfa Aesar, 99.997 %), and a glassy carbon working electrode (BASi, 3 mm diameter), unless otherwise noted. Glassy carbon electrodes were polished (alumina slurry with Millipore Type 1, 18.2 MΩ, water) and dried prior to experiments. The Ag/Ag^+ electrode was stored in 1 M NaCl in water between measurements and was periodically checked relative to $FCN^{4-/3-}$ to ensure against potential drift. All experiments were performed at 20±1 °C, unless otherwise noted. Electrode potentials were converted to the $FCN^{4-/3-}$ standard using the conversion, $E_{FCN4-/3-} = E_{Ag/Ag+} - 0.241$ V for aqueous experiments and $E_{FCN4-/3-} = E_{Ag/Ag+} - 0.235$ V for experiments in DMF. Aqueous experiments were conducted using Millipore Type 1, 18.2 MΩ, water. For each experiment, an initial starting point for the uncompensated Ohmic loss was measured using the R_u test function in the CHI Electrochemical Workstation Version 18.05. The resistance was corrected to $R_{corr} = 0.85\ R_u$. This R_{corr} was inputted manually into the iR compensation menu in the CHI Electrochemical Workstation Version 18.05. Current density values are reported relative to the geometric surface area of the glassy carbon working electrode. Experiments were conducted in 20mL scintillation vials with septa sealing to the openings, unless otherwise noted. A razorblade was used to cut slits into the septa to accommodate insertion of the electrodes. Prior to data collection, the working compartment and solutions were purged with N_2 for > 10 min to ensure the complete removal of residual O_2. During data collection, the headspace of the working compartment was subject to a constant positive pressure of N_2.

Method for Obtaining Diffusion Coefficients

Diffusion coefficients were obtained by measuring CVs at varying scan rates using an automated program.[8] The diffusion coefficient was then calculated from the slopes of peak current densities (A) plotted against the square root of the scan rate (V s^{-1}) and the Randles- Ševčík equation, shown below. The values obtained for the cathodic and anodic peaks were averaged to obtain a single diffusion coefficient.

Randles- Ševčík Equation:

$$i_p = 0.4463nFAC\left(\frac{nFvD}{RT}\right)^{\frac{1}{2}}$$

i_p = peak current (A)
n = number of electrons transferred
F = Faraday's constant (C mol^{-1})
A = area of the electrode (cm^2)
C = concentration (mol cm^{-3})
v = scan rate (V s^{-1})
D = diffusion coefficient (cm^2 s^{-1})
R = ideal gas constant (J K^{-1} mol^{-1})
T = temperature (K)

Method for Bulk Electrolysis

Bulk electrolysis to generate the reduced $CcCO_2^{-}$ was performed using an H-cell with a glass frit separator. The working and counter electrodes were carbon (Duocel® RVC Foam, 100 PPI, 3% relative density), and a pseudo-Ag/Ag^{+} reference electrode (described in general Electrochemical Methods) was used in the working chamber. A solution of $CcCO_2HPF_6$ in 1 M pH 12 potassium phosphate buffer was added to the working chamber of the H-cell and a solution of 1 M pH 12 potassium phosphate buffer was added to the counter chamber. Both sides were equipped with stir bars. Using the chronopotentiometry method on the CHI 760E potentiostat, current was passed until all of the $CcCO_2$ was reduced to $CcCO_2^{-}$. The solution in the working chamber would turn from pale yellow to red in color. The $CcCO_2^{-}$ solution could then be used for subsequent experiments.

For the van't Hoff experiments, a 2 mM solution of $CcCO_2^{-}$ was generated using bulk electrolysis with 5 mA current. Equal volumes of 2 mM $CcCO_2$ and 2 mM $CcCO_2^{-}$ were then combined to create a solution of 2 mM $CcCO_2$ at 50% state of charge. M_4L_6 host was then dissolved in this solution for the experiments.

Method for Obtaining van't Hoff Plots Electrochemically

Stock solutions of the appropriate species (typically, 2 mM $CcCO_2$, and 2 mM M_4L_6) were prepared in 1 M pH 12 buffer (potassium phosphate, unless otherwise indicated). The pH of the solution was not corrected at different temperatures. All measurements were conducted at 50% state of charge. A diagram of the setup for electrochemical van't Hoff experiments is shown below (**Figure S10**). Two 20 mL scintillation vials equipped with magnetic stir bars and capped with rubber septa are connected via a length of PTFE tubing (approximately 8 inches in length, 1/16th inch diameter). A razor blade was used to cut small slits in the rubber septa beforehand to accommodate the tubing and electrodes. One vial (left) contains the working electrode and is placed in an oil bath on an IKA RCT basic stir plate, with the thermocouple (IKA ECT-D5, error of ±0.2 K) inserted into the oil bath. The other vial (right) contains the counter electrode and reference electrode and is placed in a water bath on another IKA RCT basic stir plate with a thermocouple (IKA ECT-D5, error of ±0.2 K) inserted into the water bath. 6 mL of the stock solution was transferred to the vial containing the working electrode *via* syringe. Then, the connecting tube was lowered into the solution. The solution was transferred by positive pressure into the other vial containing the reference electrode and counter electrode until the solution levels were approximately even on both sides. Both sides were stirred at 400 rpm throughout the experiment. The oil bath side of the setup was heated in increments of 5 °C from the ambient temperature (20 °C) to 55 °C. At each temperature, the solution was allowed to equilibrate for approximately 5 minutes, until the open circuit potential stabilized. The temperature of the water bath was monitored using a thermometer and the thermocouple attached to the stir plate and stayed constant throughout the experiment. The open circuit potential at each temperature was plotted against the temperature difference between the two baths and the data was fit using a linear least squares regression. The slope gave the Seebeck temperature coefficient α in mV K^{-1}, which could be converted to cal mol^{-1} K^{-1} to obtain ΔS. Data was obtained in triplicate.

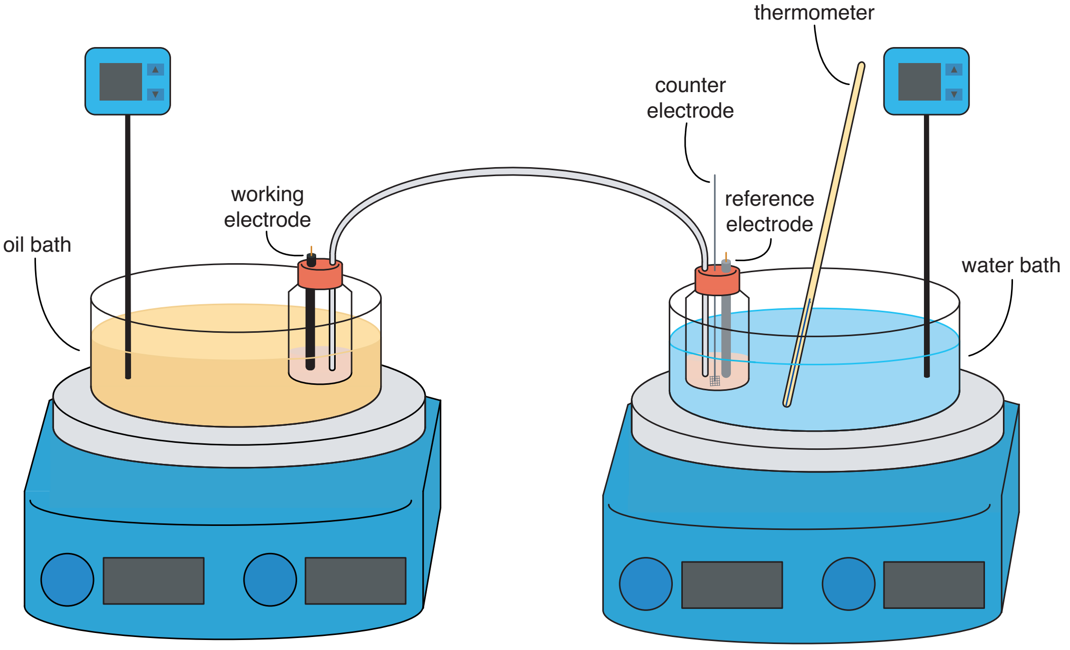

Figure S1: Diagram of electrochemical van't Hoff experimental setup

Validation of Electrochemical van't Hoff Method

The electrochemical van't Hoff method was validated using the Nernst Equation, shown below:

At room temperature (298 K): $\ln(Q) = \frac{zFE_{cell}}{RT} = \frac{E_{cell}}{0.05916\ V}$

Where Q is the molar ratio between oxidized and reduced species, z is the number of electrons transferred, R is the ideal gas constant, and E_{cell} is the potential.

The open circuit potential was measured at different ratios of oxidized to reduced species of $CcCO_2$ in the presence and absence of Ga_4L_6 to determine whether the electrochemical behavior obeyed the Nernst Equation.

A 2 mM solution of reduced $CcCO_2^-$ was prepared by bulk electrolysis. A scintillation vial containing mg Ga_4L_6 was prepared with a septa fitted to the opening and reference, working, and counter electrodes inserted. 1.8 mL of the $CcCO_2^-$ solution was then transferred to the scintillation vial, dissolving the Ga_4L_6. Incremental amounts of a 8 mM solution of $CcCO_2$ in its Co(III) oxidized state were then added to the vial (see **Table S1** below), i.e. first 0.05 mL of Co(III) solution was added, then an additional 0.1 mL, then an additional 0.15 mL and so on. Between additions, 10-15 minutes of equilibration time was allowed and the open circuit potential was recorded at the different molar ratios of oxidized and reduced species.

Table S1: Incremental addition volumes

	Volume of Co(III) solution added (mL)	Molarity of Co(II) (mM)	Molarity of Co(III) (mM)	Total volume (mL)	Q
1	0.05	1.95	0.22	1.85	9
2	0.1	1.85	0.62	1.95	3
3	0.15	1.71	1.14	2.1	1.5
4	0.15	1.60	1.60	2.25	1
5	0.225	1.45	2.18	2.475	0.66666667
6	0.675	1.14	3.43	3.15	0.33333333
7	2.7	0.62	5.54	5.85	0.11111111

Construction and Testing of a Thermogalvanic Heat Engine

A simplified thermogalvanic heat engine was constructed based on designs reported by Arun Majumdar and Gang Chen and coworkers (**Figure 3b**).[9,10] Two electrochemical flow cells are connected with two temperature baths in between. The solution in one flow cell is held at low temperature and the solution in the other flow cell is held at high temperature, creating a temperature differential. The difference in electrochemical potential is measured across the two flow cells at varying current densities.

The flow cell assemblies used are previously described by Fikile Brushett and Melanie Sanford and coworkers.[11,12] The electrolyte diffusers were machined from PTFE. Serpentine graphite flow fields were used. Each electrode consisted of two sheets of carbon felt (Sigracet® 29AA) with approximately 20% compression and an active cross-sectional area of 2.55 cm^2 (1.5 cm by 1.7 cm) and they were held in place with ePTFA gaskets. The solution inlet and outlet channels were sealed using base-resistant EPDM o-rings. The electrodes were each burned for 30 seconds with a butane torch prior to use in order to oxidize the surface and make the material more hydrophilic. A Nafion N211 cation exchange membrane was used to separate the two sides of the flow cell. A Cole-Parmer Masterflex® peristaltic pump (L/S Digital Drive, 100 RPM, 115/230 VAC 07522-30) featuring a two-channel rotor (L/S two-channel Easy-Load II pump head, SS rotor) was used along with a combination of Masterflex® compressible pump tubing and PFA flexible tubing. Detailed instructions for flow cell assembly are described below, along with a bill of materials (**Table S2**) and diagrams.

After assembling the device, the solutions were cycled for 30 minutes to equilibrate before measurements were performed. The open circuit potential was measured. Then, short chronopotentiometry experiments were performed to determine the current density at which the potential approached 0 V (the maximum current density). Then, a multi-step current experiment was set up on the CHI 760E potentiostat, with incremental current steps between 0 and the maximum current density. The system was allowed to equilibrate at each current step for 30 to 60 seconds while the electrochemical potential was recorded. The data from the last 5 seconds of the potential recorded at each step were averaged to obtain a potential value for that current density. The experiment was performed in triplicate and the potential values were then averaged between the three runs. From these data, the power output could be plotted against current density, and the maximum power output could be calculated by fitting the data to a second order polynomial.

The efficiency (η) of the system was calculated according to the equation shown below, derived from Arun Majumdar and coworkers' work:[9]

$$\eta = \frac{Power\ output}{Power\ input} = \frac{I \times V}{I \times T_{hot}(\alpha_1 - \alpha_2) + \left(Q_1 C_{p1} \rho_1 + Q_2 C_{p2} \rho_2\right) \times (T_{hot} - T_{cold})}$$

Where:
I = Current density (A)
V = Electrochemical potential (V)
T_{hot}, T_{cold} = Hot side and cold side temperatures, respectively (K)
α = Seebeck temperature coefficient (V K^{-1})
Q = Flow rate (kg s^{-1})
C_p = Heat Capacity (J K^{-1} kg^{-1})
ρ = Density of the solution (kg m^{-3})

The subscripts 1 and 2 denote each electrolyte side of the device respectively.

The efficiency is then reported as a percentage of the Carnot efficiency (η_c), which is calculated by the following equation:

$$\eta_c = 1 - \frac{T_{cold}}{T_{hot}}$$

Table S2: Bill of materials used for the thermogalvanic device

Part	Details	Supplier	Catalog number	Quantity
Electrolyte diffuser	PTFE, see below for diagram	Machined in house		4
Serpentine flow field	Tokai G347B Graphite, 1/8” thick sheet, see below for diagram	Machined in house		4
Bolts (Pkg. of 25)	18-8 Stainless Steel Socket Head Cap Screw, 1/4"-28 Thread, 2-1/4" Length, pkg 25	McMaster-Carr	92196A340	8
Nuts (Pkg. of 100)	Type 18-8 Stainless Steel Hex Nut, 1/4"-28 Thread Size, 7/16" Wide, 7/32" High	McMaster-Carr	91845A105	8
Teflon Tape	Teflon tape, high density 3M 48, 1/4" Wide	McMaster-Carr	4934A13	1
Banana Plugs	Pomona Electronics 3263 Test Plugs & Test Jacks 6/32 STUD UNINS PLUG BU-00241	Mouser Electronics	565-3263	4
Carbon Conductive Paste	MG Chemicals carbon conductive paste, 25 mL (1 oz, 27 g)	Mouser Electronics	590-847-25ML	1
Swagelok PFA Tube Fittngs	PFA Male Connector, 1/8" tube to 1/8" NPT	Swagelok	PFA-220-1-2	8
Swagelok PFA Notcher	PFA 1/8" Groove Notcher	Swagelok	MS-GC-2	1
Swagelok PFA Tubing	1/8" PFA Swagelok Tubing, 100ft	Swagelok	PFA-T2-030-100	1
Masterflex Pump Tubing	L/S® Precision Pump Tubing, C-Flex® ULTRA, L/S 16; tubing I.D. 3.10 mm (0.122”), hose barb size 0.125”, 25 ft	VWR	MFLX06434-16	1
Nafion Membrane	Nafion™ Membrane N211, 30cm x 30cm; cut to 2 1”x1” squares	Ion Power	N211-US-0.30x0.30	2
EPDM O-rings	Steam-Resistant EPDM O-Ring, 1/16 Fractional Width, Dash Number 014, pkg 100	McMaster-Carr	9464K19	8
Tubing Adapter	Barbed Fitting for Use with Chemicals Reducer, for 1/8" X 1/16" Tube ID, 150 Deg F Max Temp, Packs of 10	McMaster-Carr		4
Cable Tie	Polypropylene cable ties	McMaster-Carr	70215K61	4
Cable Tie Tensioning Tool	Light Duty Cable Tie Tensioning Tools, Narrow, Standard	McMaster-Carr	5401T11	1
Teflon sheet cutter	Silhouette Cameo 4	Amazon		1
Teflon gaskets for flow fields	Cut from Sterling Seal & Supply Expanded PTFE Sheet, .5 mm Thick, 12" x 6", White (1 sheet), see below for cutting pattern	Amazon		4

Teflon gaskets for O-rings	Cut from Sterling Seal & Supply Expanded PTFE Sheet, .5 mm Thick, 12" x 6", White (1 sheet), see below for cutting pattern	Amazon		4
Loctite super glue plastic bonding system	Clear Superglue for Plastic, Cyanoacrylate Adhesive Instant Glue, Quick Dry - 0.14 fl oz Activator, .07 fl oz Glue, Pack of 1	Amazon		1

Quantities are listed for assembly of 1 device. Some of the materials are discarded after each run of the experiment, as described in the procedure below, so for repeated experiments additional quantities should be ordered.

Below are detailed instructions for preparing the components and then assembling the device:

Preparing the tubing
There are 3 types of tubing that must be prepared in advance: (1) connecting the solution reservoirs to through the pump and the cold bath to the first flow cell, (2) connecting the first flow cell through the hot bath to the second flow cell, and (3) connecting the second flow cell to the solution reservoirs. **Figure S2** shows the tubing types labeled on the device diagram.

Tubing (1) consists of: a 15-inch length of PFA tubing connected to a 10-inch length of Masterflex pump tubing via a tubing adapter. On the other end of the Masterflex pump tubing, a 75-inch length of PFA tubing was connected via a tubing adapter. The 75-inch tubing was coiled to fit into the cold bath, and then the other end could be attached to a flow cell using a Swagelok PFA tube fitting. The ends of the PFA tubing that connected to the flow cell or the tubing adapters were cut perpendicular to the length of the tubing. The ends of the PFA tubing that are inserted into the solution reservoir were cut at an angle. The ends of the tubing that connect to the flow cell are notched with the Swagelok groove notcher.
Tubing was joined via a tubing adapter as follows: Loctite super glue was applied to the surfaces of the tubing adapter that interfaced with the PFA tubing before these two components were connected, and the glue was allowed to dry overnight. The joint was then encased in hot glue for structural support, using a hot glue gun. Then the other end of the tubing adapter was inserted into the Masterflex pump tubing and secured with a cable tie and tightened using the cable tie tightening tool.

Tubing (2) consists of: a 75-inch length of PFA tubing, coiled to fit into the hot bath. Both ends of this tubing were cut perpendicular to the length of the tubing, to connect to the two flow cells.

Tubing (3) consists of: a 15-inch length of PFA tubing, connecting the second flow cell to the solution reservoir. The end of the tubing connecting to the flow cell was cut perpendicular to the length of the tubing and notched with the Swagelok groove notcher, and the end connecting to the solution reservoir was cut at an angle.

Each device run requires 2 each of tubing types (1), (2), and (3), for each of the 2 electrolyte solutions. New tubing was used for each experiment.

Preparing the membrane and electrodes
Per flow cell, 4 sheets of graphite felt (cut to 1.5 cm by 1.7 cm) should be prepared by burning with a butane torch for 30 seconds. The graphite felt can be held using metal tweezers and passed through the flame of a butane torch until it glows red. This oxidizes the surface of the graphite to make it more hydrophilic and improve contact with the aqueous electrolyte solutions.

The Nafion N211 cation exchange membrane (cut to 2.5 cm by 2.5 cm) is prepared by heating in 2 M aqueous KOH solution at 60 °C overnight. Then the membrane was rinsed thoroughly with deionized water and stored in a 4 M aqueous KCl solution between experiments. Per flow cell, one membrane is required.

Assembling the flow cells
The threads of the banana plugs are coated with carbon conductive paste, and then screwed to the protruding handle of the flow field. Excess carbon conductive paste is wiped off with a Kimwipe tissue.

The threads of the Swagelok PFA tube fittings are wrapped 3 times with heavy duty Teflon Tape, and then screwed to the electrolyte diffusers, tightening with a wrench until there is a 1/16-inch to 1/8-inch gap between the diffuser and the nut. Overtightening of the fitting can result in leakage.

Figure S3 shows an exploded diagram of the components of the flow cell and how they are assembled. First, an electrolyte diffused is laid flat and the PTFE O-ring gaskets are inserted in the indentations, followed by the EPDM O-rings. The bolts are inserted through the diffuser, and 4-inch lengths of PFA tubing are used as alignment pins and inserted into the alignment pin holes. Then, the graphite flow field is laid on top of the electrolyte diffuser, using the alignment pins for positioning. The PTFE gasket is added next, again using the alignment pins for positioning. Two sheets of graphite felt are carefully stacked on top of one another, and positioned in the opening of the PTFE gasket, on top of the flow field, using metal tweezers. Then, the Nafion N211 membrane (2.5 cm by 2.5 cm) is placed on top of the graphite felt using tweezers. Next, a second PTFE gasket is placed on top, using the alignment pins for positioning. Another two sheets of graphite felt are stacked on top of one another and then placed in the opening of the gasket, using metal tweezers. Then the second flow field is placed on top, using the alignment pins for positioning. The nuts are hand-tightened gently to the bolts in a crosswise sequence. A torque wrench (McMaster-Carr, catalog number 5871A52) is used to tighten the bolts in a crosswise sequence in increments: first to 4 lb, then 7 lb, and finally 10 lb.

Assembling the device
Figure S2 shows the connectivity of all the device components. The flow cells and the tubing should first be connected in the correct order. Then, the tubing coils should be positioned into the respective cold and hot baths. The solution reservoirs were 40-mL centrifuge tubes capped with rubber septa. Small holes were poked through the septa using metal tweezers so that the angled end of the PFA tubing could be inserted through.

The temperature baths were calibrated as follows: The cooling bath was adapted from a photochemical reactor with 6 vial wells. The PFA tubing was coiled into each well for maximum contact with the cooling block. The heating bath was a continuously stirred water bath on a heating plate (IKA ECT-D5). The PFA tubing was weighted with a lead ring to ensure it remained submerged throughout the experiment. The temperature baths, tubing sections through which tubing flowed between the baths and flow cells, and the two flow cells were all insulated generously with glass wool jacketed in aluminum foil. Prior to conducting the experiment with the device, the temperature of the system was tested using 1 M pH 12 potassium buffer solution. The temperature bath was set to temperature and the buffer solution was flowed through at 150 $\mu L\ min^{-1}$ flow rate. The temperature of the solution flowing out of the subsequent flow cell was measured. The temperature of the bath was then adjusted accordingly until the solution flowing out of the flow cell reached the desired temperature. The cooling bath was set to 1 °C to achieve

a cold flow cell temperature of 10 °C and the heating bath was set to 65 °C to achieve a hot flow cell temperature of 50 °C.

A 0.1 M solution of $CcCO_2^-$ was generated using bulk electrolysis with 50 mA current. The H-cell chamber can accommodate 5 mL of solution. 4 mL solution of the 0.1 M $CcCO_2^-$ was then added to 16 mL of 0.1 M $CcCO_2$ to create a 20 mL solution of 0.1 M $CcCO_2$ at 20% state of charge. Fe_4L_6 host was then dissolved in this solution to create the host-guest electrolyte solution. A 20 mL solution containing 1.2 M KI and 0.01 M I_2 in 1 M K_3PO_4 was prepared for the other side of the device. These concentrations were chosen to balance the state of charge and concentration of potassium ions on either side. The solutions were stored in colution reservoirs of 40-mL centrifuge tubes sealed with rubber septa. After connecting the tubing for the device, the PFA tubing was inserted into the septa but kept above the solvent level in the centrifuge tubes. The solutions were degassed for 20 minutes with a purge needle through the septa. The purge needle was removed after 20 minutes, and the centrifuge tubes were then kept under flow of nitrogen at positive pressure for the duration for the experiment. The PFA tubing was then inserted to the bottom of the centrifuge tubes.

For recreating the reported performance of potassium ferri/ferrocyanide, the following electrolyte solutions were used. On the ferri/ferrocyanide side, the solution contained 0.3 M potassium ferricyanide and 0.3 M potassium ferrocyanide. On the iodide/triiodide side, the solution contained 0.15 M I_2 and 2.1 M KI.

Once the device is assembled and electrolyte solution is added to the solution reservoirs, the experiment is ready to be conducted. The working electrode clip was attached to the banana plug on the catholyte (iodide/triiodide) side of the cold cell. The reference electrode was connected to the counter electrode, which was then attached to the banana plug on the catholyte side of the hot cell. The banana plugs on the anolyte (Fe_4L_6 solution or ferri/ferrocyanide) sides of the hot and cold cells were connected to each other with a conductive wire.

After the experiment, deionized water is cycled through the tubing and flow cells the tubing to rinse out most of the electrolyte solutions. The device can then be dissembled. PTFE gaskets, O-rings, and graphite electrodes are all discarded. The remaining components are rinsed thoroughly with deionized water and allowed to air dry. The Nafion N211 membrane was stored in a 4 M aqueous KCl solution between experiments.

Figure S2: Diagram of device assembly

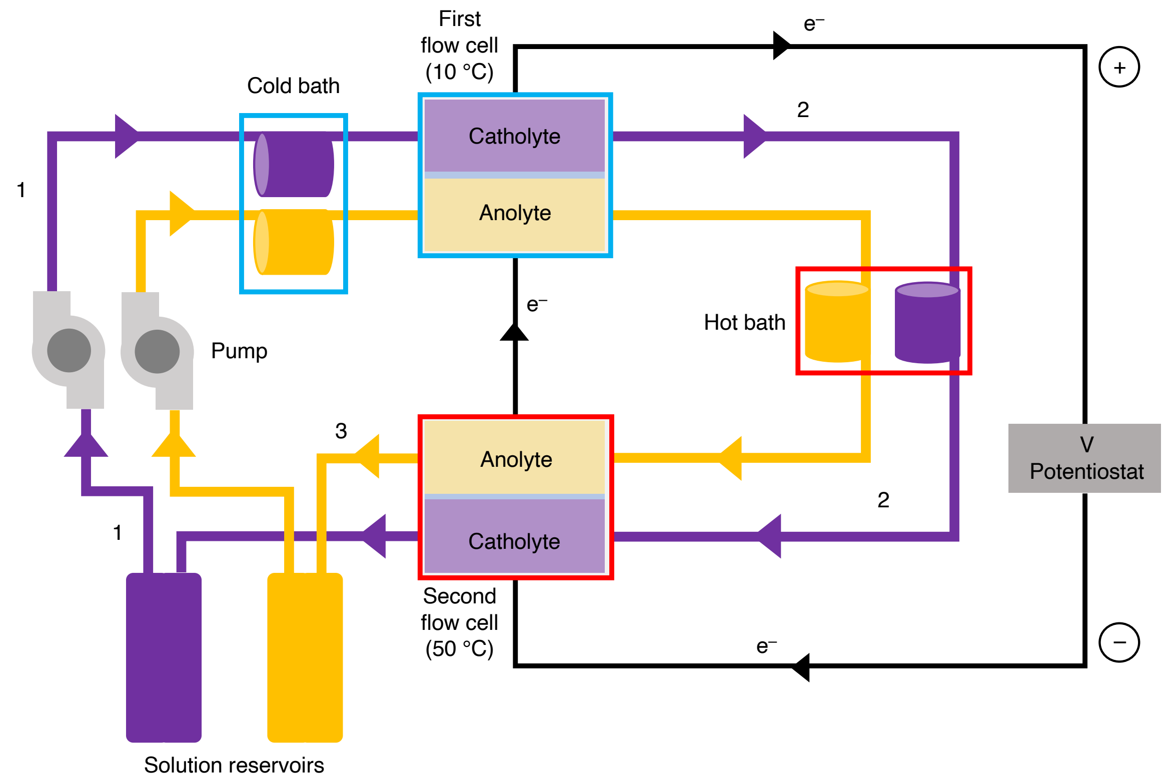

The tubing lengths are labeled as (1), (2), and (3). See instructions above for details on how to prepare each of these tubing lengths.

Figure S3: Diagram of electrochemical flow cell assembly

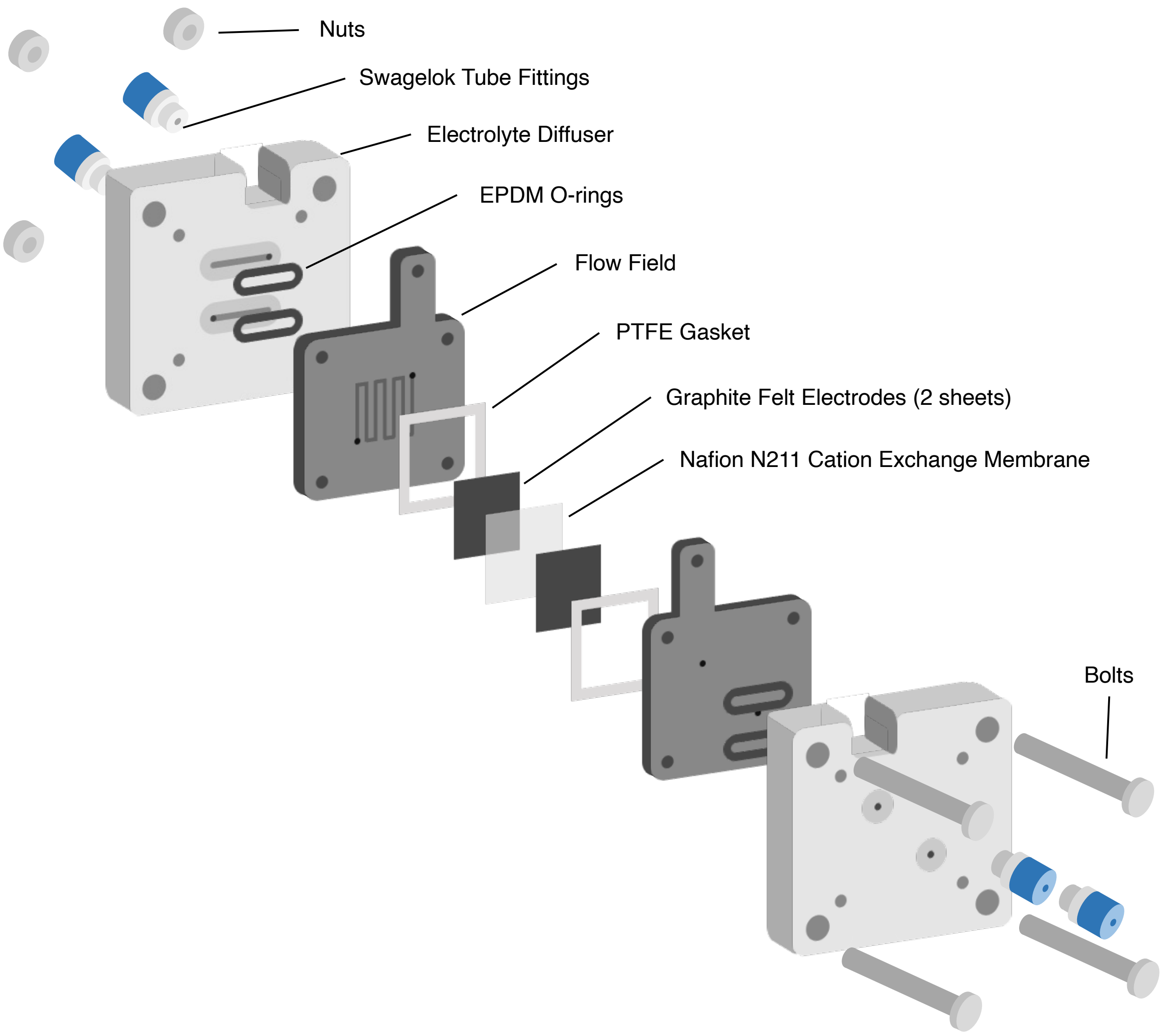

Figure S4: Diagrams for dimensions of electrolyte diffusers and graphite flow fields

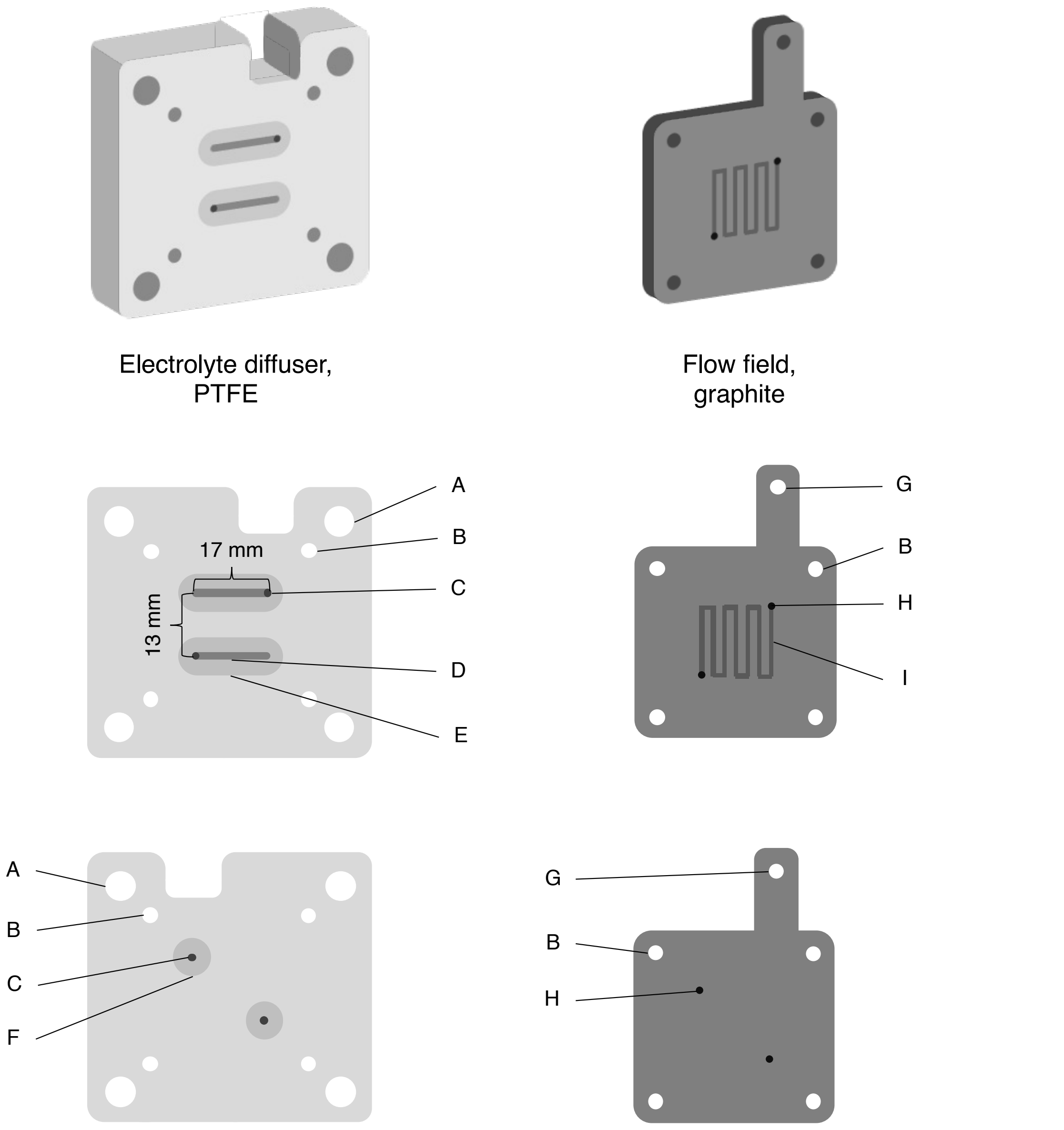

A: Holes for nuts and bolts
B: Holes for alignment pins for cell assembly
C: Solution flow channels through electrolyte diffuser block, 2 mm diameter
D: Solution flow well, 17 mm long, 2 mm wide, and 7 mm deep, spaced 13 mm apart so that the channels labeled C align with the channels labeled H
E: Indentation to fit O-rings to seal the solution channels
F: Female fitting to accommodate Swagelok tube fittings to connect PFA tubing to the cell
G: Hole for attaching banana plugs
H: solution flow channels through flow field, 1 mm diameter
I: Serpentine flow field, consisting of 7 parallel vertical wells, each 16 mm long, 1 mm wide, and 0.5 mm deep, with 1 mm wide spacing in between each well. The vertical wells are connected in a serpentine fashion with horizontal wells that are 1.5 mm wide.

Figure S5: Diagrams for cutting ePTFA gaskets

The gaskets for the flow cell were cut according to the dimensions shown below from a 0.5 mm-thick PTFE sheet using the Silhouette Cameo 4 cutter.

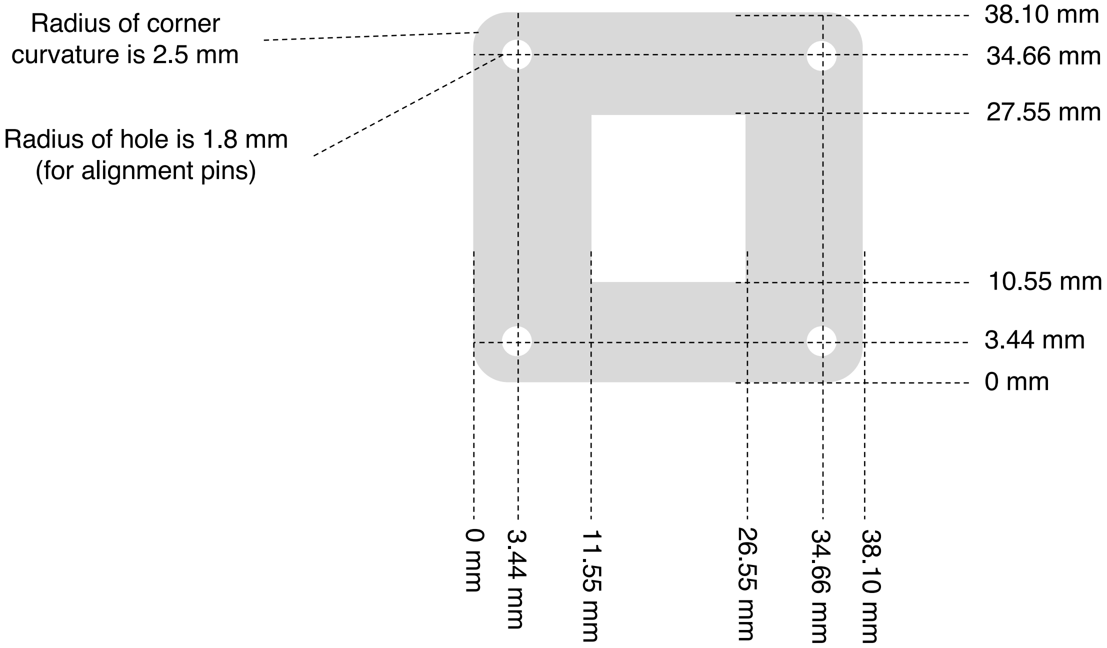

An O-ring gasket was employed to provide better sealing in the flow cell. This gasket was cut to the dimensions shown below from a 0.5 mm-thick PTFE sheet using the Silhouette Cameo 4 cutter. In the assembly of the flow cell, this gasket was placed in the O-ring indentation of the electrolyte diffuser before the EPDM O-ring.

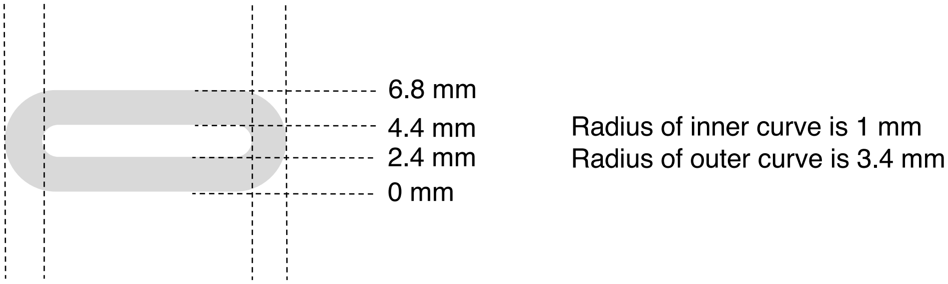

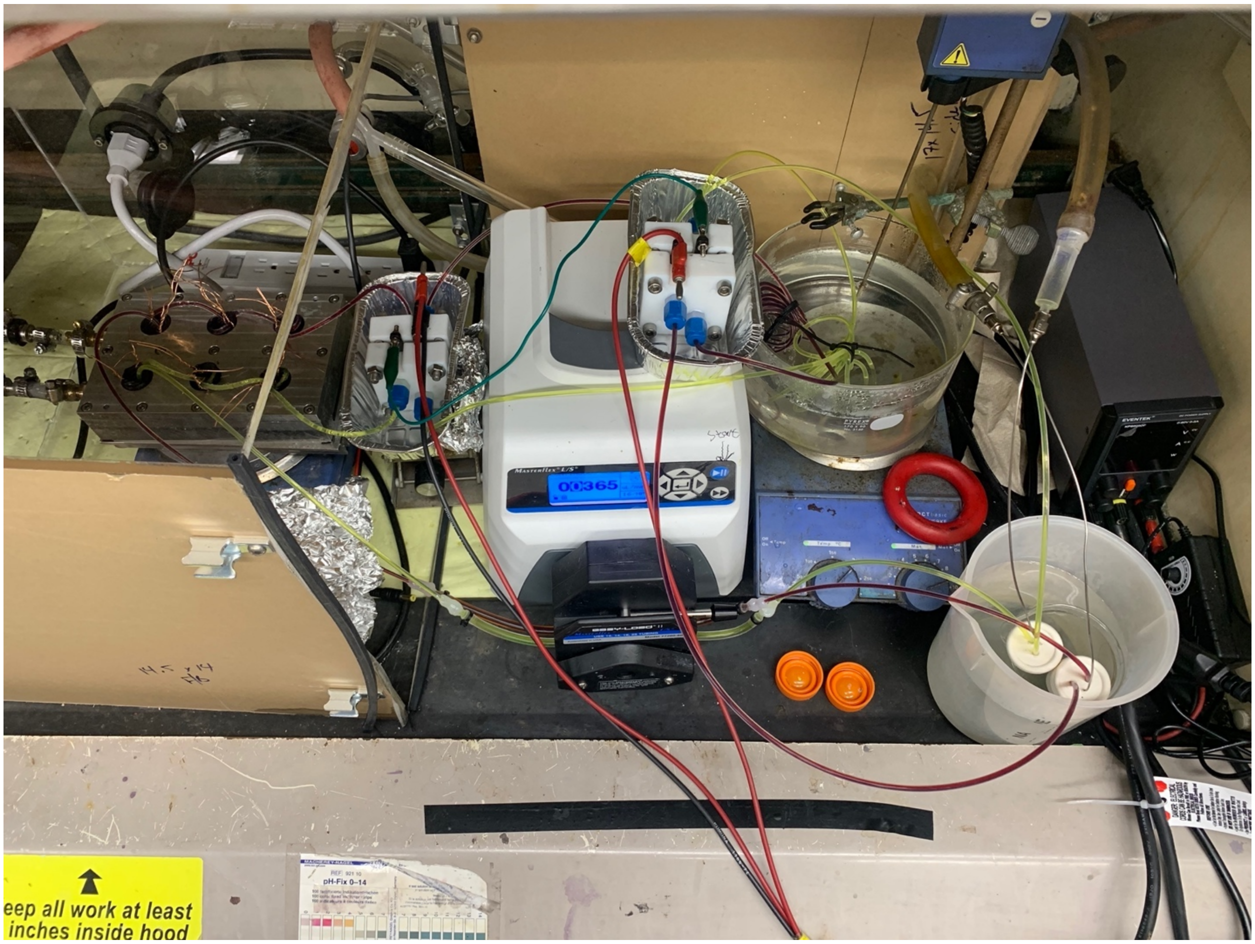

Figure S6: Picture of the thermogalvanic device. Insulation removed for clarity.

Other Characterization Methods

Method for Determining Solubility

A saturated solution was created by dissolving increasing amounts of the compound of interest until a visible amount of undissolved solid remained. The solution was then allowed to stir overnight. If the solution was homogenous the following day, more compound was added and the solution was stirred overnight again. If a visible amount of undissolved solid remained the following day, the solution was assumed to be saturated. The solution was filtered through a 20-micron syringe filter before proceeding.

The concentration of Ga_4L_6 in a saturated solution in 1 M pH 12 potassium phosphate buffer was assayed by ^{1}H NMR. The saturated solution was prepared using D_2O and was first diluted 10x by adding 50 µL of the saturated solution to 450 µL of a solution of 55 mM sodium tosylate, an internal standard. The concentration of the diluted solution could then be calculated from its NMR spectrum. This concentration was then multiplied by 10 to obtain the concentration of the saturated solution.

The concentration of Fe_4L_6 in a saturated solution was assayed by UV-visible spectroscopy. Fe(III) is paramagnetic, precluding our ability to characterize the solution using NMR. The absorption coefficient (ε) of Fe_4L_6 was determined to be 9960 M^{-1} cm^{-1} in 1 M pH 12 potassium phosphate buffer at 480 nm^{-1}. The saturated solution was diluted 2000x by adding 1 µL to 2 mL of 1 M pH 12 potassium phosphate buffer. The concentration of the diluted solution was measured by UV-vis spectroscopy, and then multiplied by 2000 to determine the concentration of the saturated solution.

Method for Determining Density

The density of the electrolyte solutions used for the thermogalvanic device were determined after cycling. A 20-mL scintillation vial was tared on a precision balance with accuracy to 0.1 mg. Using a micropipette, 1 mL of the electrolyte solution was added to the scintillation vial, and the weight was recorded. The balance was then tared, and the procedure was repeated a total of five times to obtain an average value.

Cyclic Voltammograms

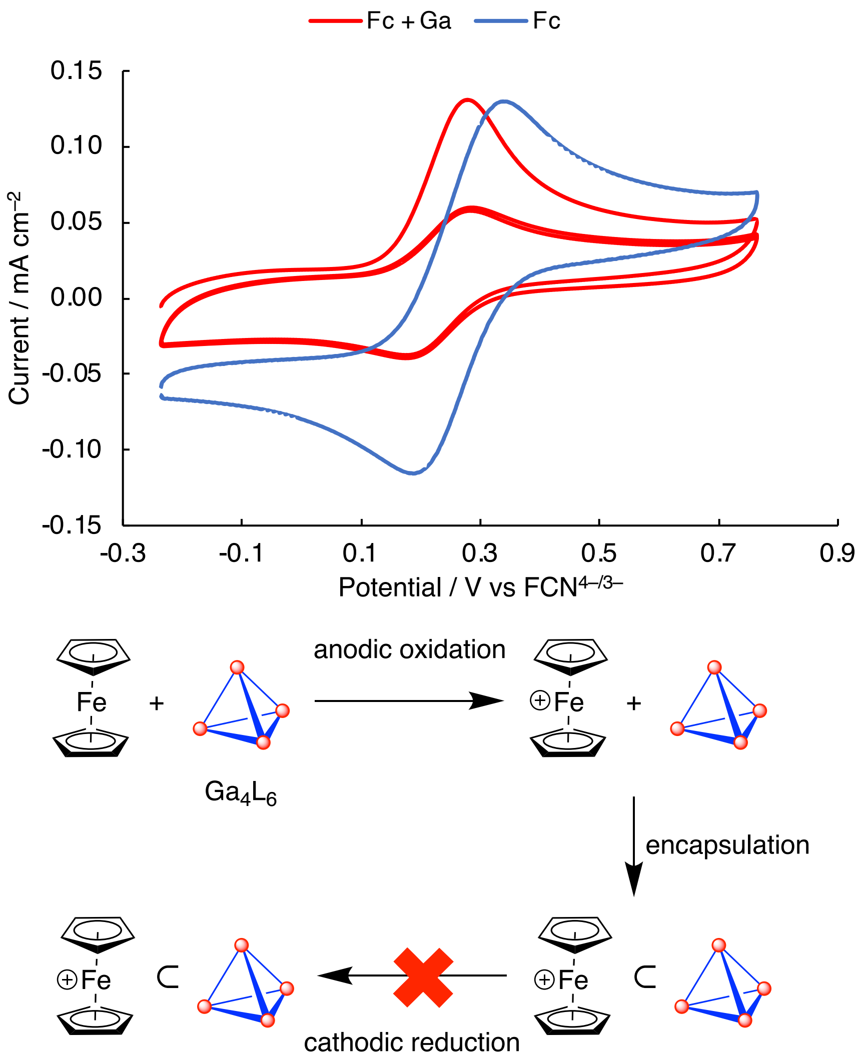

Figure S7: Electrochemical behavior of ferrocene in the presence of Ga_4L_6

Top: CV of 2 mM ferrocene (blue) in 100 mM KPF_6 in DMF, 100 mV/s scan rate, scanning oxidatively from –0.241 V vs FCN$^{4-/3-}$, 1 cycle; and CV of 2 mM ferrocene and 2 mM $[Ga_4L_6]^{12-}$ (red) in 100 mM KPF_6 in DMF, 100 mV/s scan rate, scanning oxidatively from 0 V vs FCN$^{4-/3-}$, 3 cycles.

Bottom: A scheme for the electrochemical behavior of ferrocene in the presence of $[Ga_4L_6]^{12-}$

$[Ga_4L_6]^{12-}$ was found to inhibit the redox activity of the encapsulated guest at the electrode. In the CV, a peak was observed during the first oxidative scan, followed by muted redox behavior on subsequent scans. Increasing the reductive driving force by scanning to lower potentials did not revive the redox behavior of the guest (**Figure S8**).

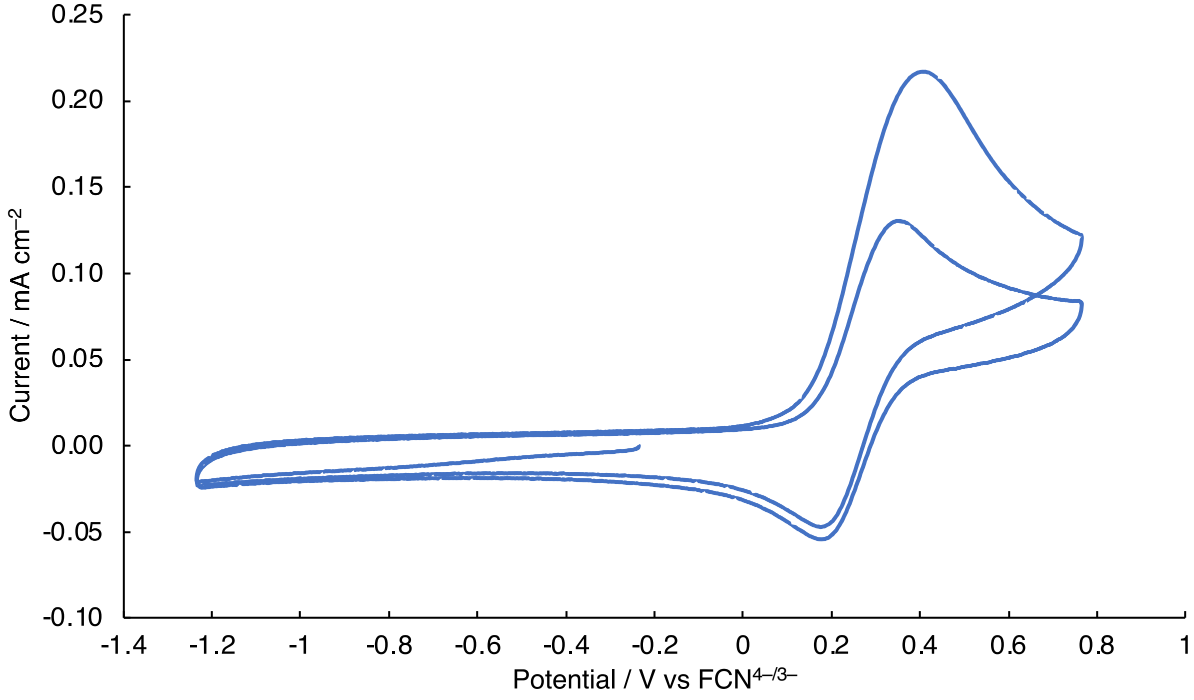

Figure S8: CV of 2 mM Ferrocene with 2 mM Ga_4L_6 from –1.235 V to 0.765 V vs FCN$^{4-/3-}$

100 mM KPF_6 in DMF
Scanning oxidatively from –0.235 V vs FCN$^{4-/3-}$
2 cycles

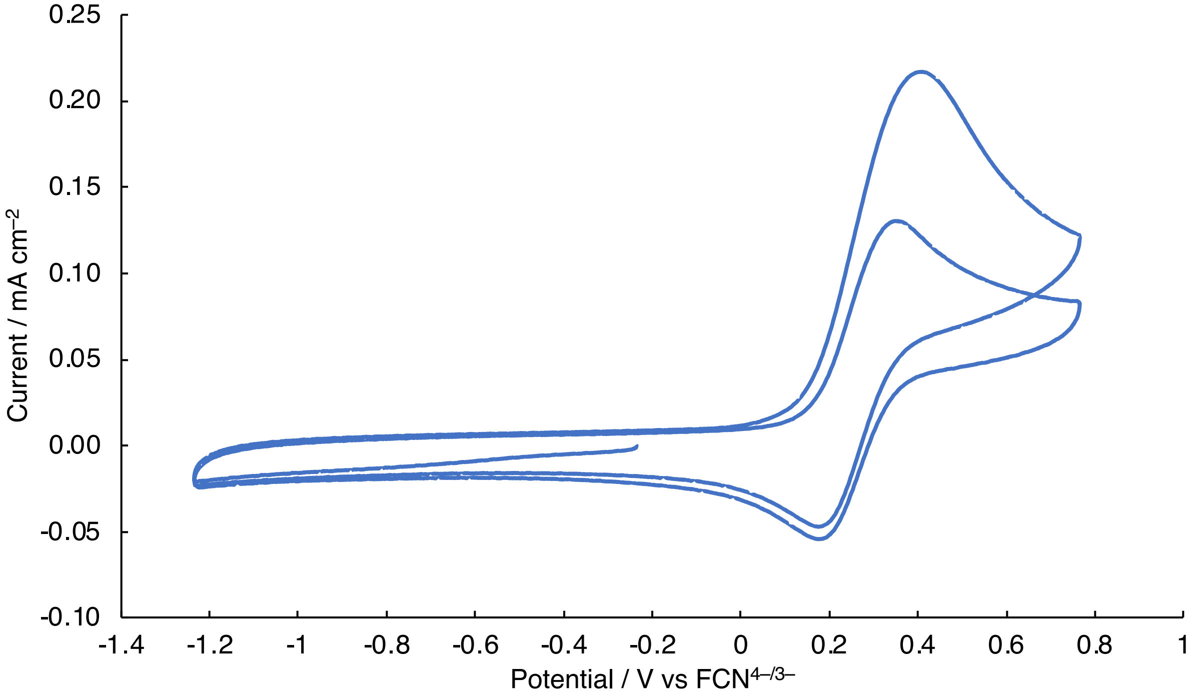

Figure S9: CV of 2 mM ferrocene and 2 mM Ga_4L_6 with Pt working electrode

100mM KPF_6 in DMF
Pt working electrode (BASi, 3 mm diameter)
Scanning oxidatively from –1.235 V vs FCN$^{4-/3-}$
2 cycles

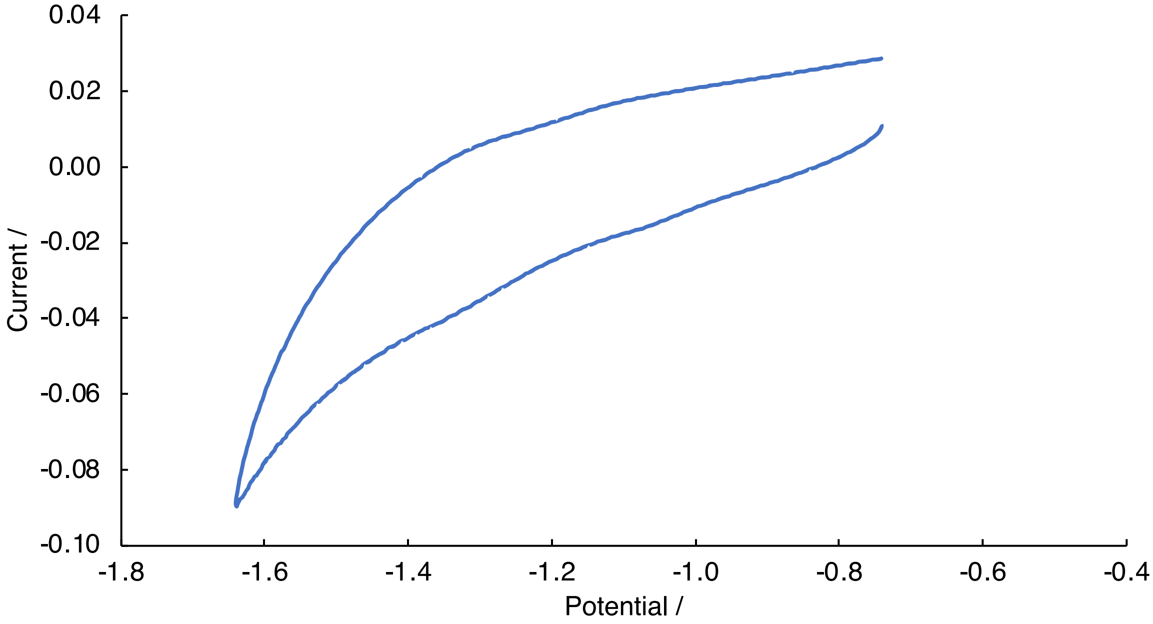

Figure S10: CV of 2 mM $K_{11}[Cp*_2Co\subset Ga_4L_6]$

1 M pH 12 potassium phosphate buffer in water
Scanning reductively from –0.741 V vs $FCN^{4-/3-}$
1 cycle

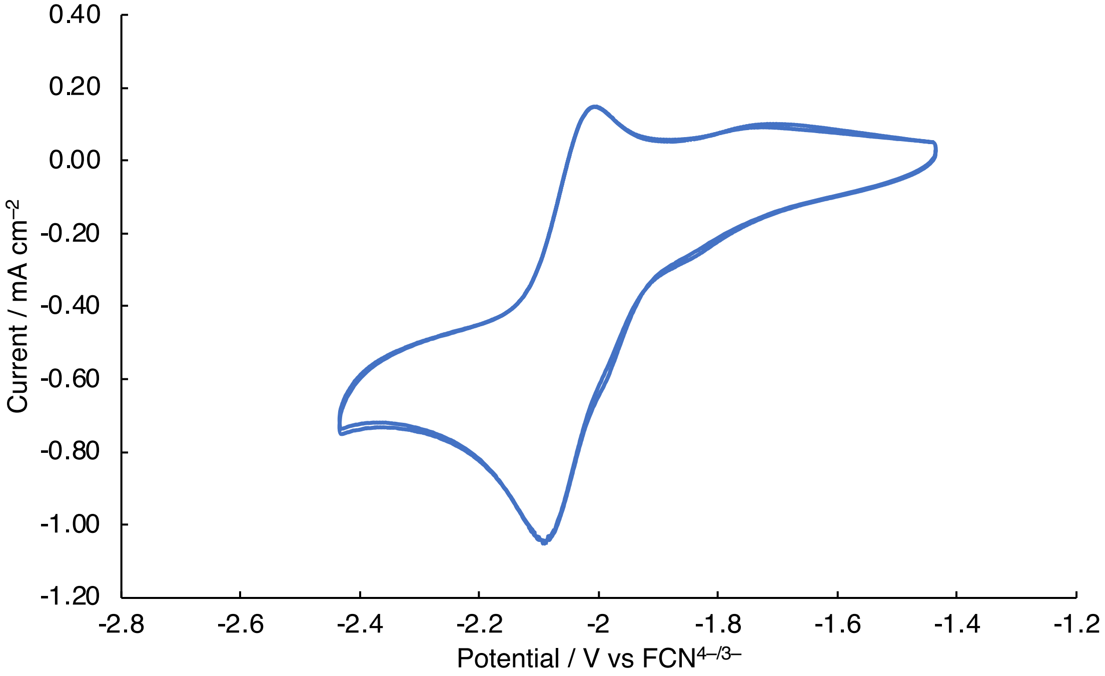

Figure S11: CV of 2 mM $NEt_4^+ \subset Ti_4L_6$

100 mM KPF_6 in DMF
Scanning reductively from –1.441 V vs $FCN^{4-/3-}$
3 cycles

Variable Scan Rate Cyclic Voltammograms

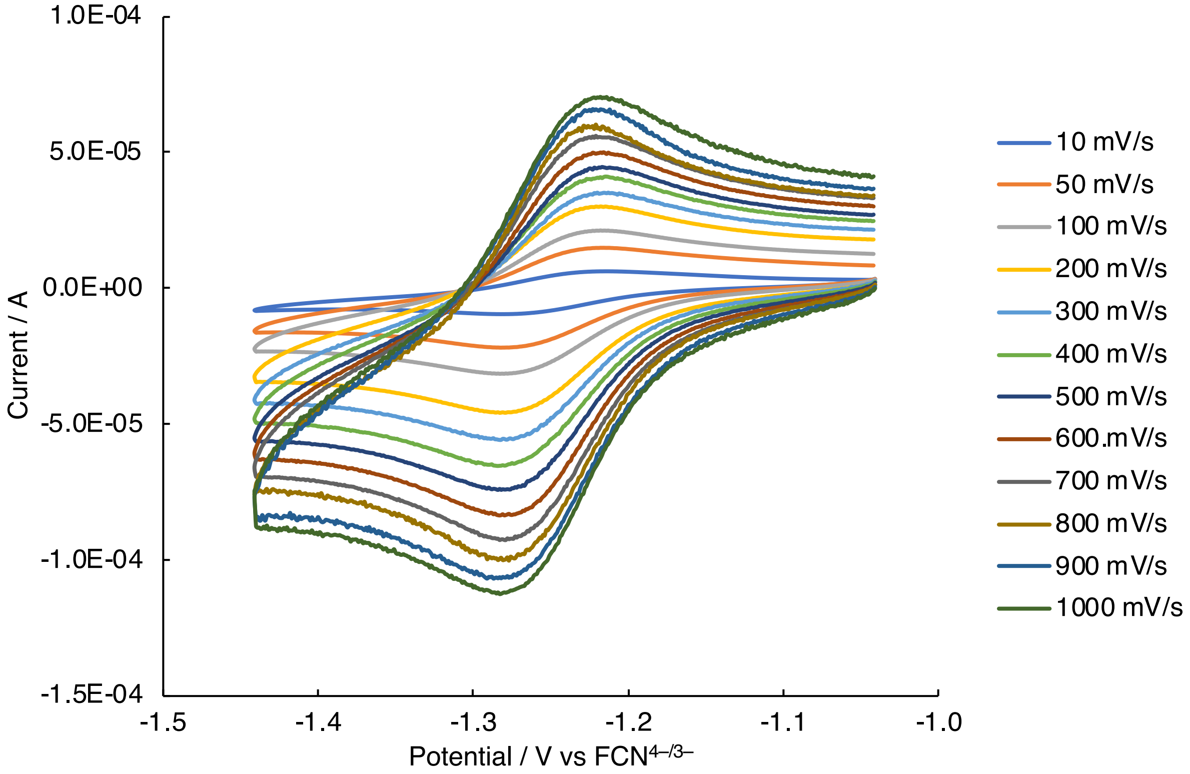

Figure S12: CVs of 2 mM $CcCO_2$ with varying scan rates

1 M pH 12 potassium phosphate buffer in water
Scanning reductively from –1.041 V vs $FCN^{4-/3-}$

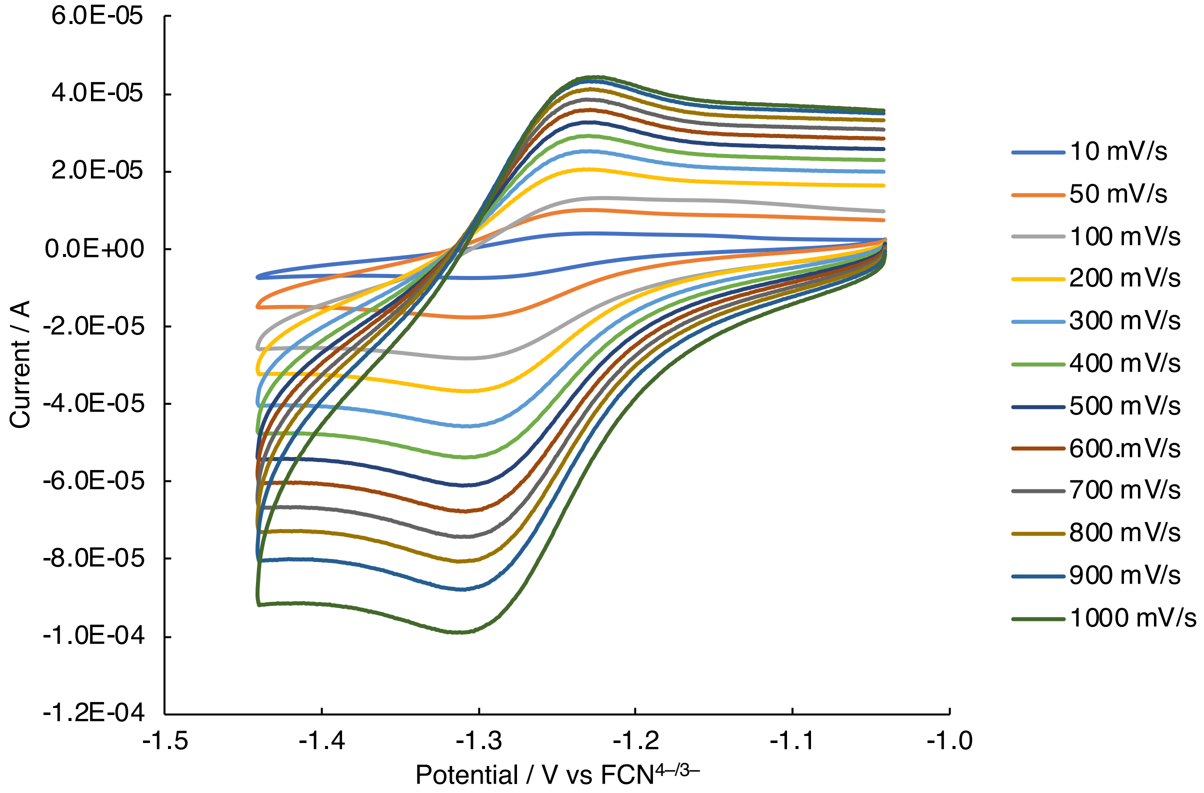

Figure S13: CVs of 2 mM $CcCO_2$ + 2 mM Ga_4L_6 with varying scan rates

1 M pH 12 potassium phosphate buffer in water
Scanning reductively from –1.041 V vs $FCN^{4-/3-}$

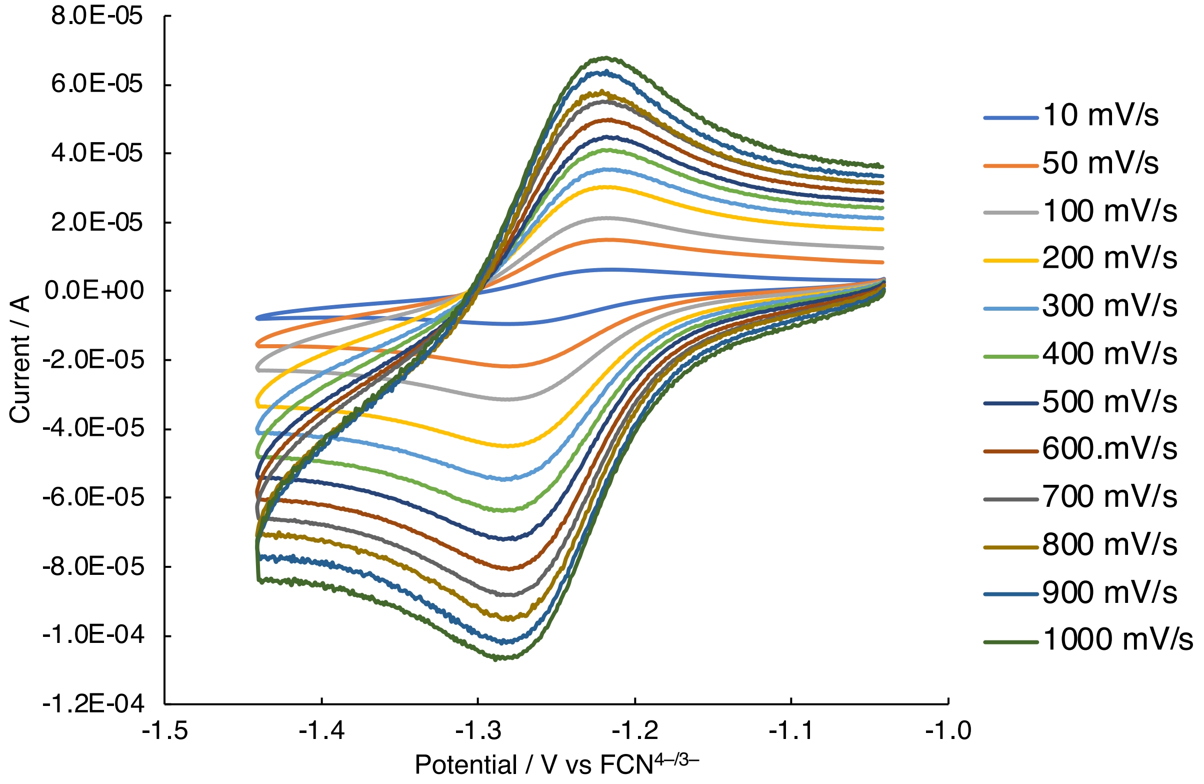

Figure S14: CVs of 2 mM $CcCO_2$ + 2 mM Ga_4L_6 + 2.4 mM PEt_4I with varying scan rates

1 M pH 12 potassium phosphate buffer in water
Scanning reductively from –1.041 V vs $FCN^{4–/3–}$

Diffusion Coefficients

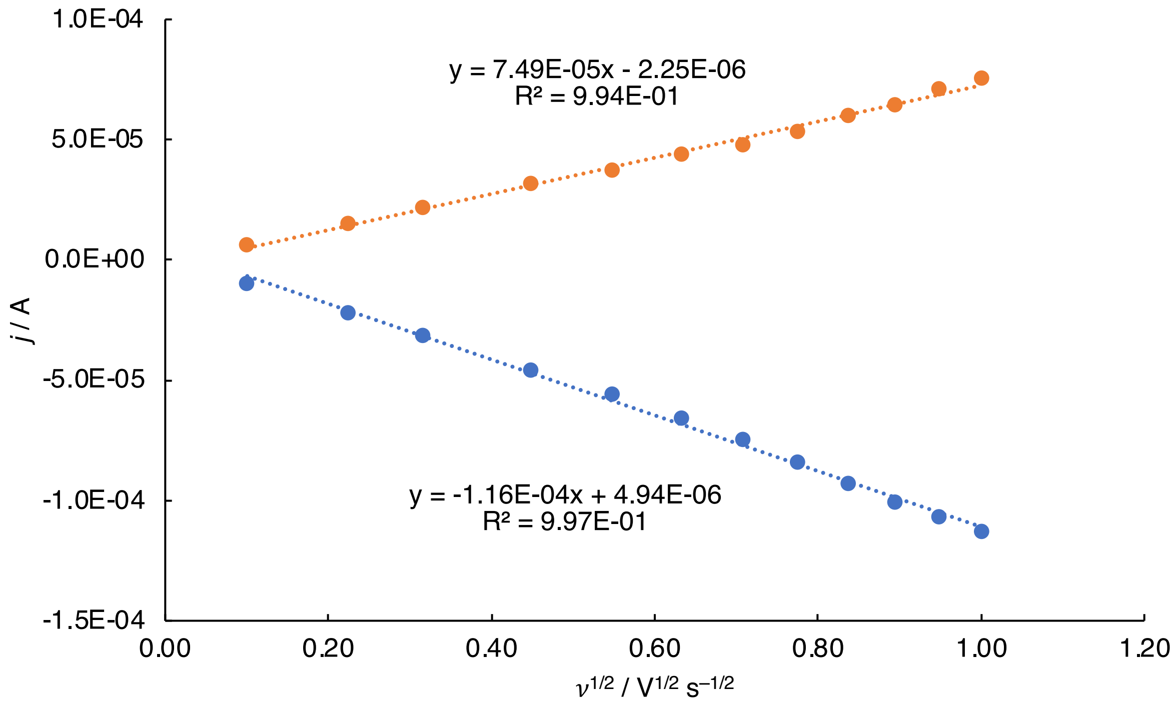

Figure S15: Cathodic and anodic peak current densities versus square root of scan rate for oxidation and reduction of $CcCO_2$

Table S3: Diffusion coefficient of $CcCO_2$

D_{ox}	$2.39(3) \times 10^{-7}$ cm s^{-1}
D_{red}	$5.71(4) \times 10^{-7}$ cm s^{-1}

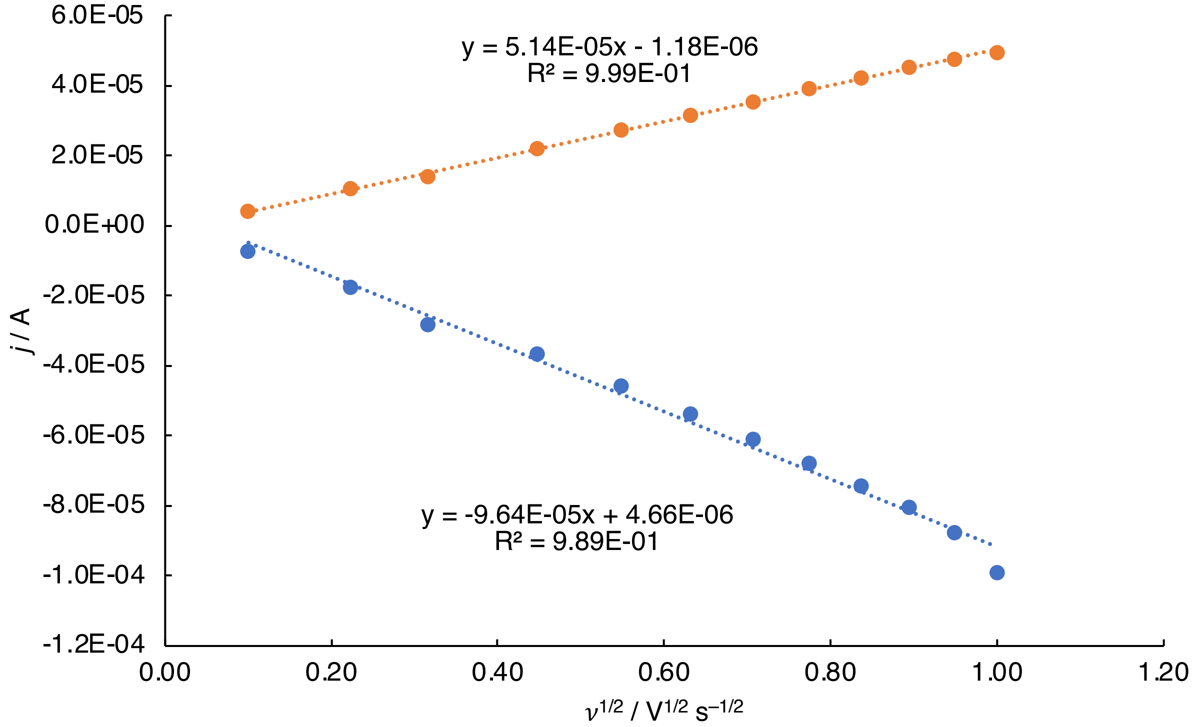

Figure S16: Cathodic and anodic peak current densities versus square root of scan rate for oxidation and reduction of $CcCO_2$ with Ga_4L_6

Table S4: Diffusion coefficient of $CcCO_2$ with Ga_4L_6

D_{ox}	$1.12(2) \times 10^{-7}$ cm s^{-1}
D_{red}	$3.96(3) \times 10^{-7}$ cm s^{-1}

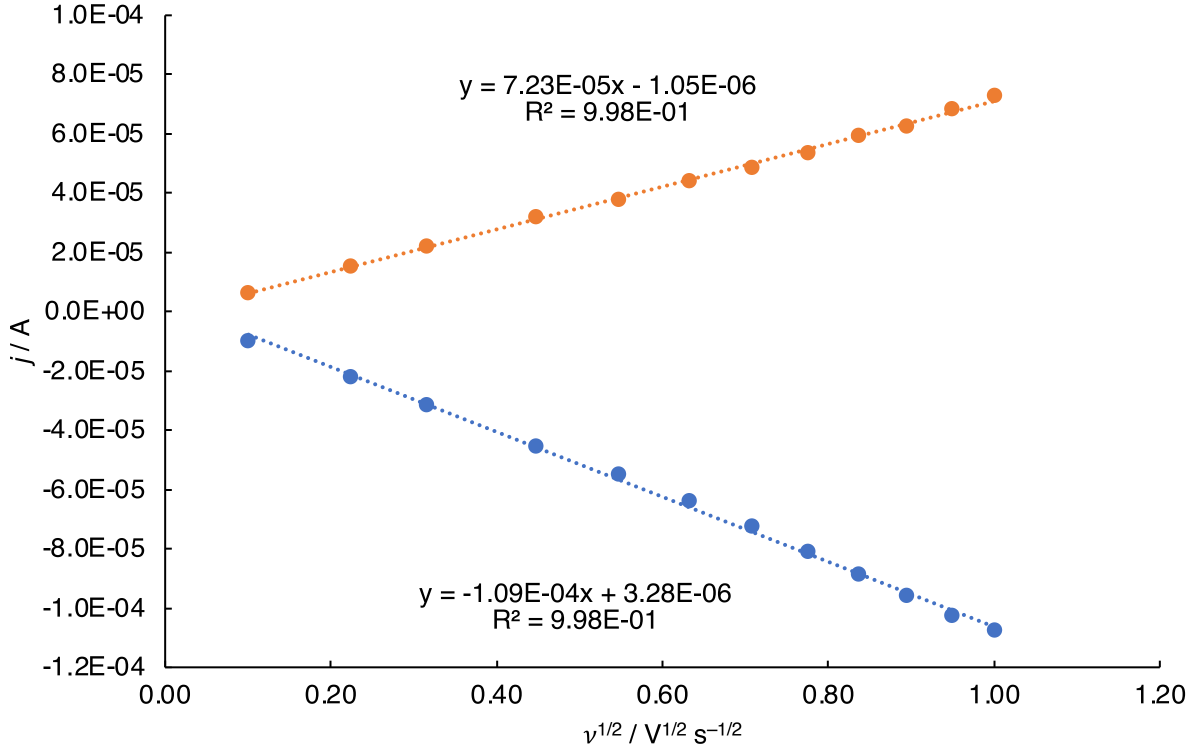

Figure S17: Cathodic and anodic peak current densities versus square root of scan rate for oxidation and reduction of $CcCO_2$ with Ga_4L_6 and PEt_4^+

Table S5: Diffusion coefficient of $CcCO_2$ with Ga_4L_6 and PEt_4^+

D_{ox}	$2.22(4) \times 10^{-7}$ cm s^{-1}
D_{red}	$5.09(5) \times 10^{-7}$ cm s^{-1}

van't Hoff Plots

Encapsulation of Cobaltocenium in Ga_4L_6

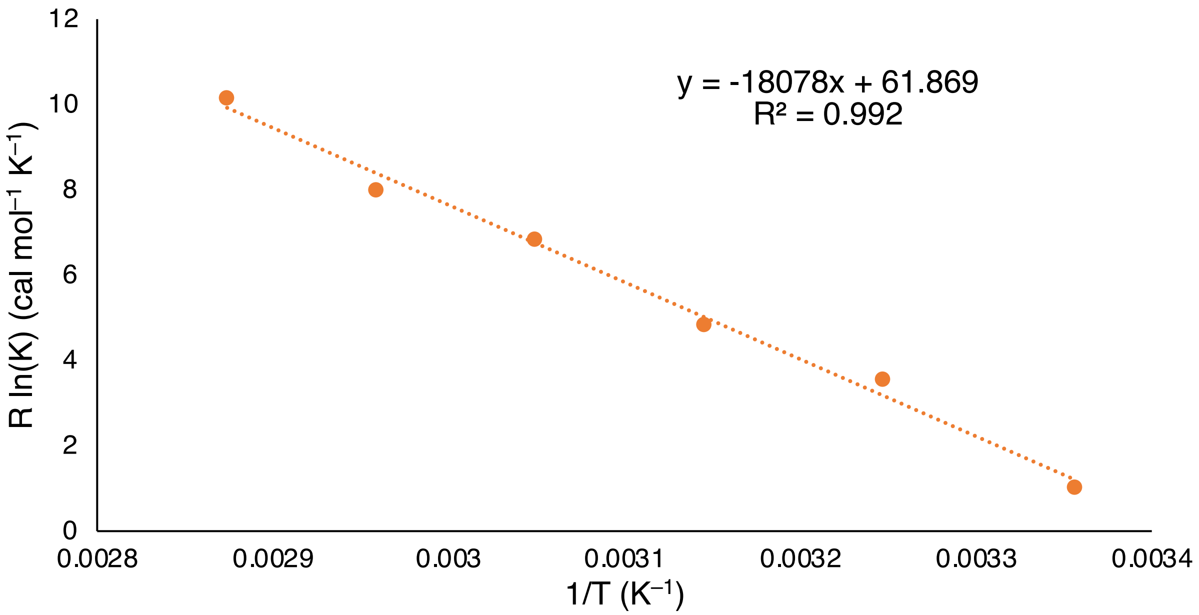

Figure S18: van't Hoff plot of 10 mM cobaltocenium hexafluorophosphate + 10 mM Ga_4L_6 Obtained using the NMR van't Hoff method.

Table S6: Data for van't Hoff plot of 10 mM cobaltocenium hexafluorophosphate + 10 mM Ga_4L_6

T (K)	K_{eq}
298	1.6803
308	6.0249
318	11.422
328	31.418
338	56.083
348	166.27

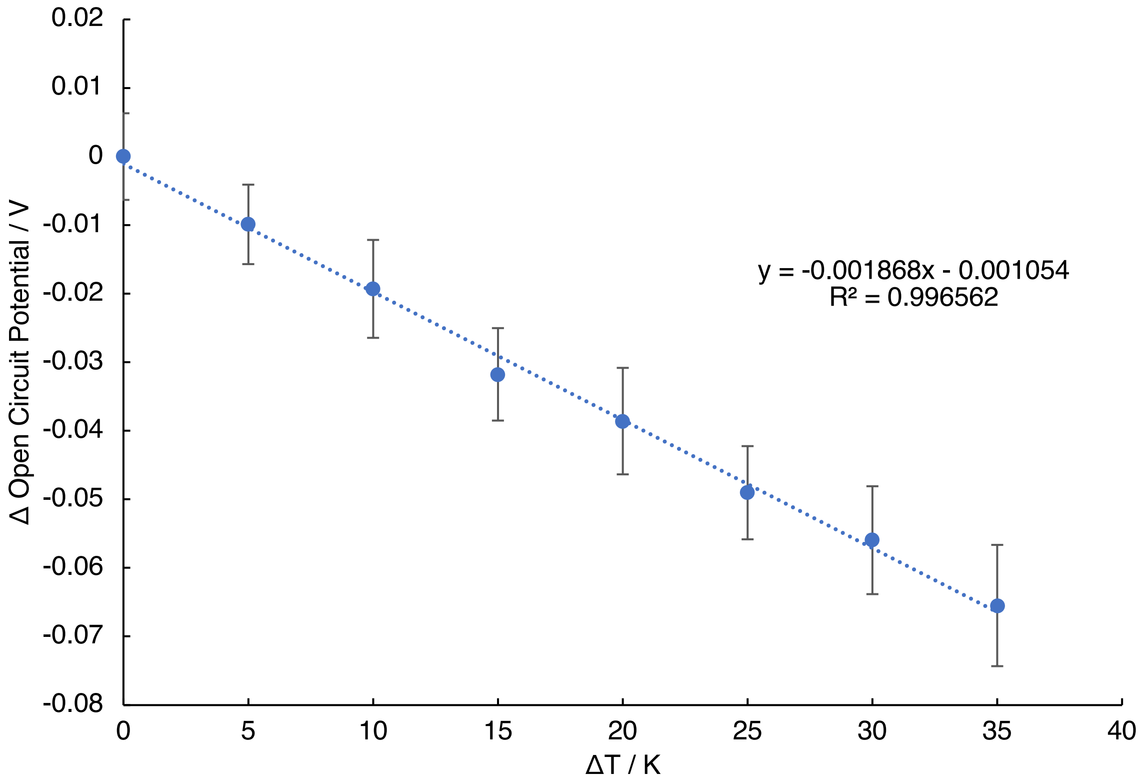

Figure S19: van't Hoff plot of 2 mM $CcCO_2$

Table S7: Data for van't Hoff plot of 2 mM $CcCO_2$

	Δ Open Circuit Potential (V)		
ΔT (K)	Run 1	Run 2	Run 3
0	0	0	0
5	-0.00842	-0.00791	-0.01334
10	-0.02088	-0.01967	-0.01734
15	-0.03261	-0.03149	-0.03118
20	-0.04125	-0.03494	-0.03955
25	-0.04921	-0.045	-0.05286
30	-0.05866	-0.05156	-0.05762
35	-0.0696	-0.05849	-0.06834

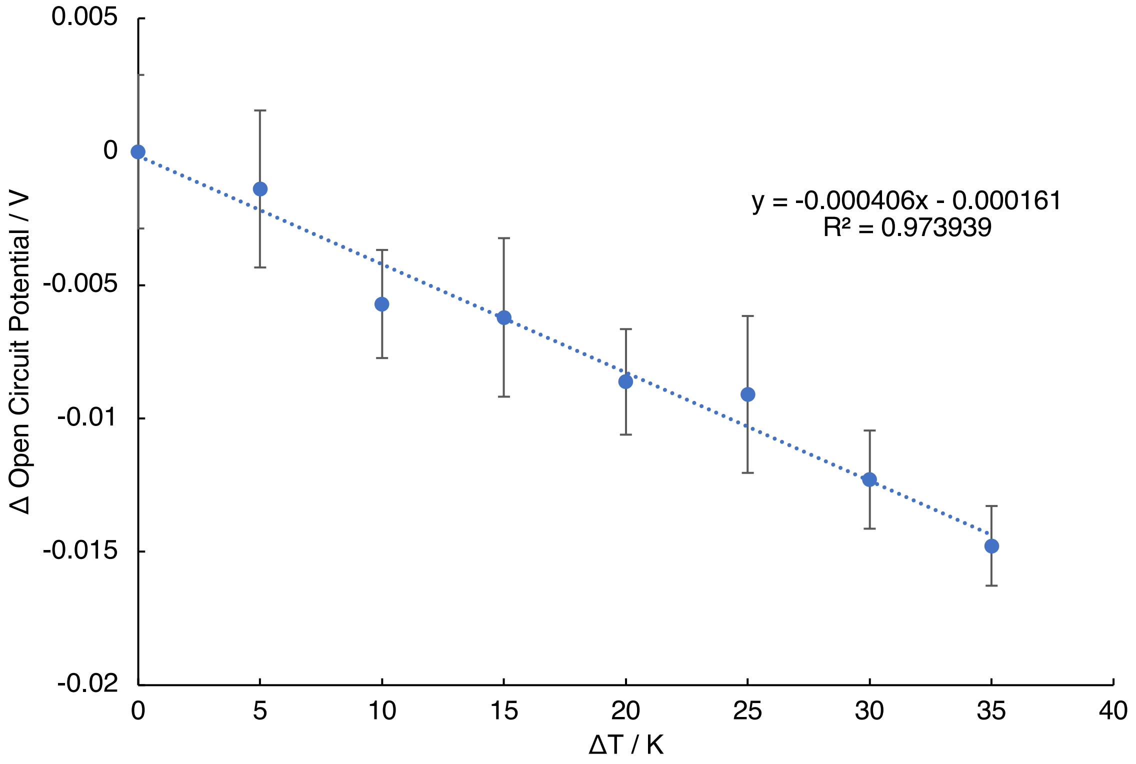

Figure S20: van't Hoff plot of 2 mM Ga_4L_6

Table S8: Data for van't Hoff plot of 2 mM Ga_4L_6

	Open Circuit Potential (V)		
ΔT (K)	Run 1	Run 2	Run 3
0	0	0	0
5	-0.00107	-0.00086	-0.00226
10	-0.00335	-0.00656	-0.00722
15	-0.00511	-0.00521	-0.00832
20	-0.00638	-0.00964	-0.00987
25	-0.00811	-0.00821	-0.01098
30	-0.01014	-0.01374	-0.01300
35	-0.01231	-0.01710	-0.01492

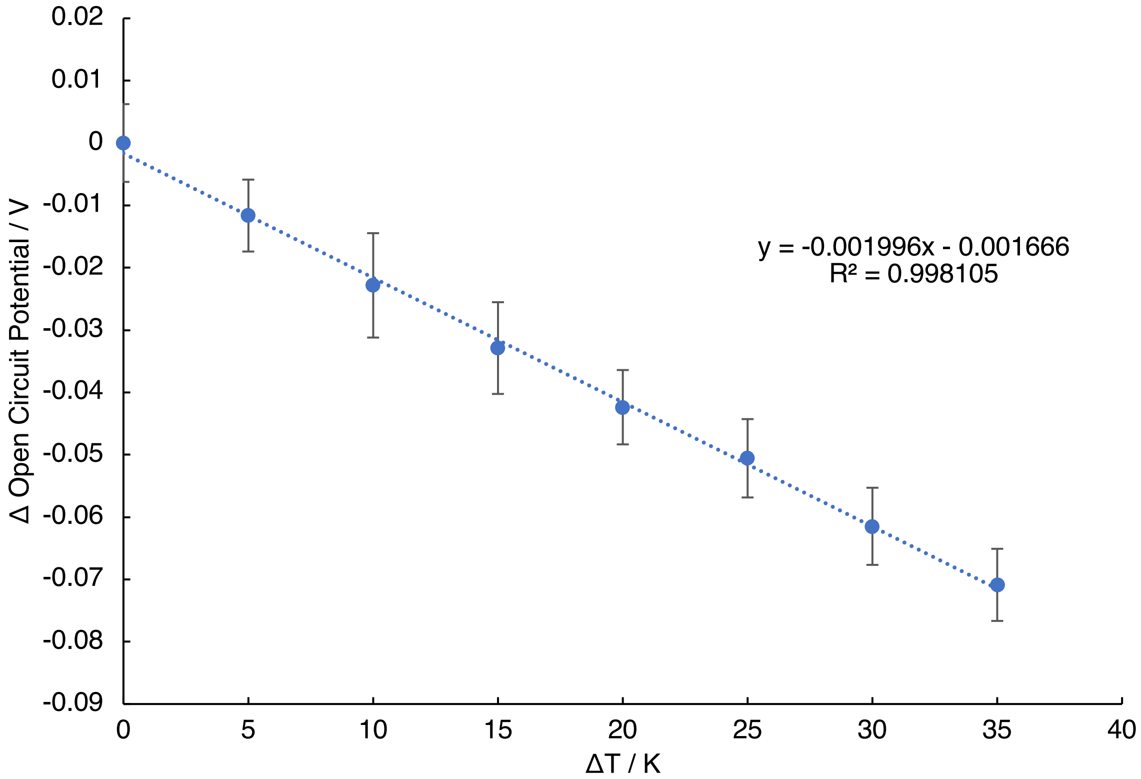

Figure S21: van't Hoff plot of 2 mM $CcCO_2$ + 2 mM Ga_4L_6 + 2 mM PEt_4I

Table S9: Data for van't Hoff plot of 2 mM $CcCO_2$ + 2 mM Ga_4L_6 + 2 mM PEt_4I

	Open Circuit Potential (V)		
ΔT (K)	Run 1	Run 2	Run 3
0	0	0	0
5	-0.01235	-0.01074	-0.01183
10	-0.01794	-0.0252	-0.02537
15	-0.02974	-0.03345	-0.0355
20	-0.04233	-0.04141	-0.04341
25	-0.04881	-0.04818	-0.05474
30	-0.0599	-0.05891	-0.06566
35	-0.07136	-0.06983	-0.07146

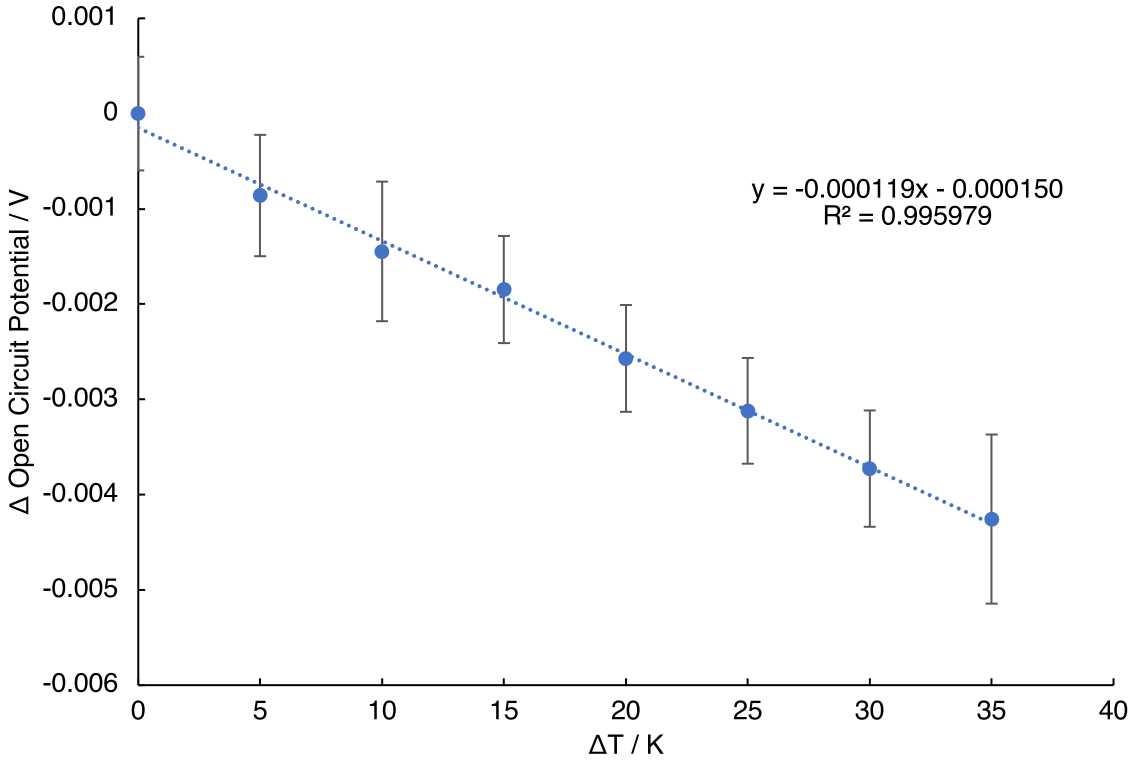

Figure S22: van't Hoff plot of pH 12 1 mM potassium phosphate buffer (control)

Table S10: Data for van't Hoff plot of pH 12 1 mM potassium phosphate buffer (control)

	Open Circuit Potential (V)		
ΔT (K)	Run 1	Run 2	Run 3
0	0	0	0
5	-0.00125	-0.00107	-0.00026
10	-0.00175	-0.00123	-0.00136
15	-0.00205	-0.00209	-0.0014
20	-0.00255	-0.00265	-0.00251
25	-0.00323	-0.00329	-0.00284
30	-0.00403	-0.00391	-0.00324
35	-0.00488	-0.0038	-0.00409

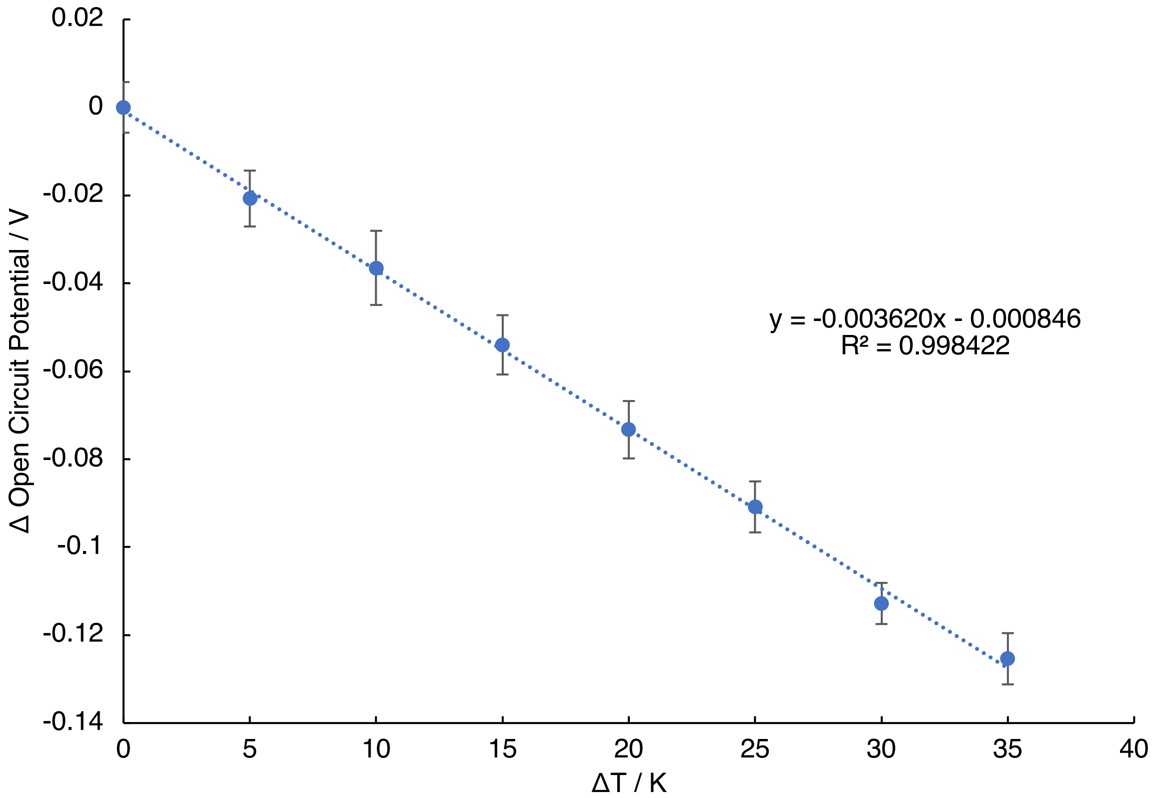

Figure S23: van't Hoff plot of 2 mM Ga_4L_6 + 2 mM $CcCO_2$

Table S11: Data for van't Hoff plot of 2 mM Ga_4L_6 + 2 mM $CcCO_2$

	Open Circuit Potential (V)		
ΔT (K)	Run 1	Run 2	Run 3
0	0	0	0
5	-0.0192	-0.02121	-0.02178
10	-0.03319	-0.04212	-0.03418
15	-0.0537	-0.05669	-0.0516
20	-0.07179	-0.07444	-0.07367
25	-0.09079	-0.09092	-0.09083
30	-0.11418	-0.11037	-0.11386
35	-0.12515	-0.12538	-0.1256

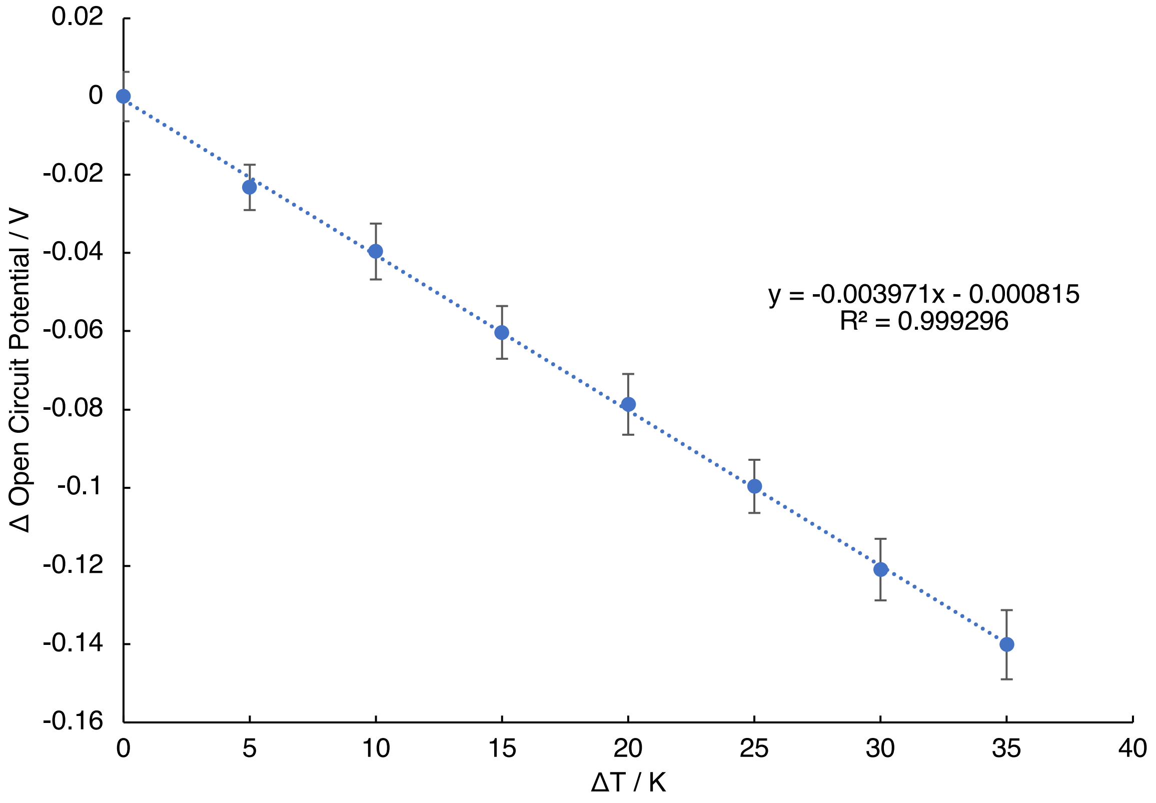

Figure S24: van't Hoff plot of 2 mM Ga_4L_6 + 2 mM $CcCO_2$ in 1 mM pH 12 sodium phosphate buffer

Table S12: Data for van't Hoff plot of 2 mM Ga_4L_6 + 2 mM $CcCO_2$ in 1 mM pH 12 sodium phosphate buffer

	Open Circuit Potential (V)		
ΔT (K)	Run 1	Run 2	Run 3
0	0	0	0
5	-0.02316	-0.02297	-0.02352
10	-0.03638	-0.04572	-0.03674
15	-0.05417	-0.0639	-0.0628
20	-0.07421	-0.0825	-0.07934
25	-0.09527	-0.10239	-0.1013
30	-0.12227	-0.12019	-0.12032
35	-0.13727	-0.14544	-0.13769

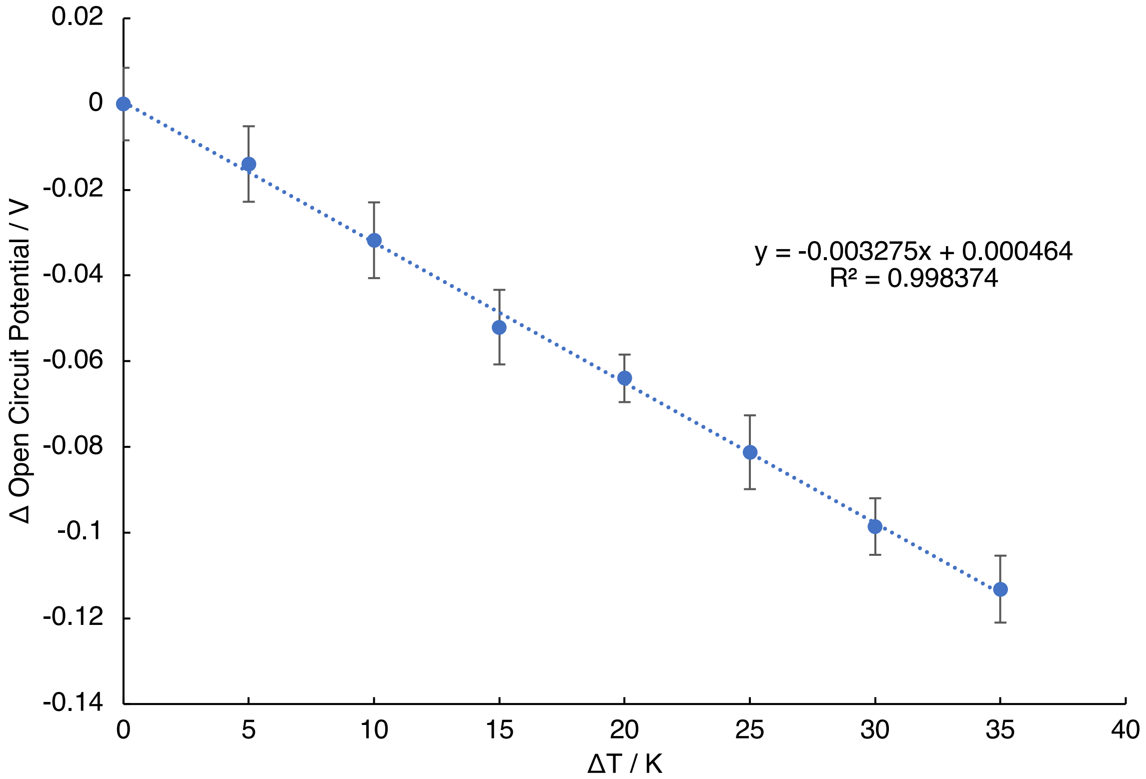

Figure S25: van't Hoff plot of 2 mM Fe_4L_6 + 2 mM $CcCO_2$

Table S13: Data for van't Hoff plot of 2 mM Fe_4L_6 + 2 mM $CcCO_2$

	Open Circuit Potential (V)		
ΔT (K)	Run 1	Run 2	Run 3
0	0	0	0
5	-0.01485	-0.01325	-0.01387
10	-0.03275	-0.03082	-0.03176
15	-0.05276	-0.05118	-0.05222
20	-0.05779	-0.06672	-0.06750
25	-0.08175	-0.08052	-0.08147
30	-0.09592	-0.09484	-0.10499
35	-0.11255	-0.11140	-0.11562

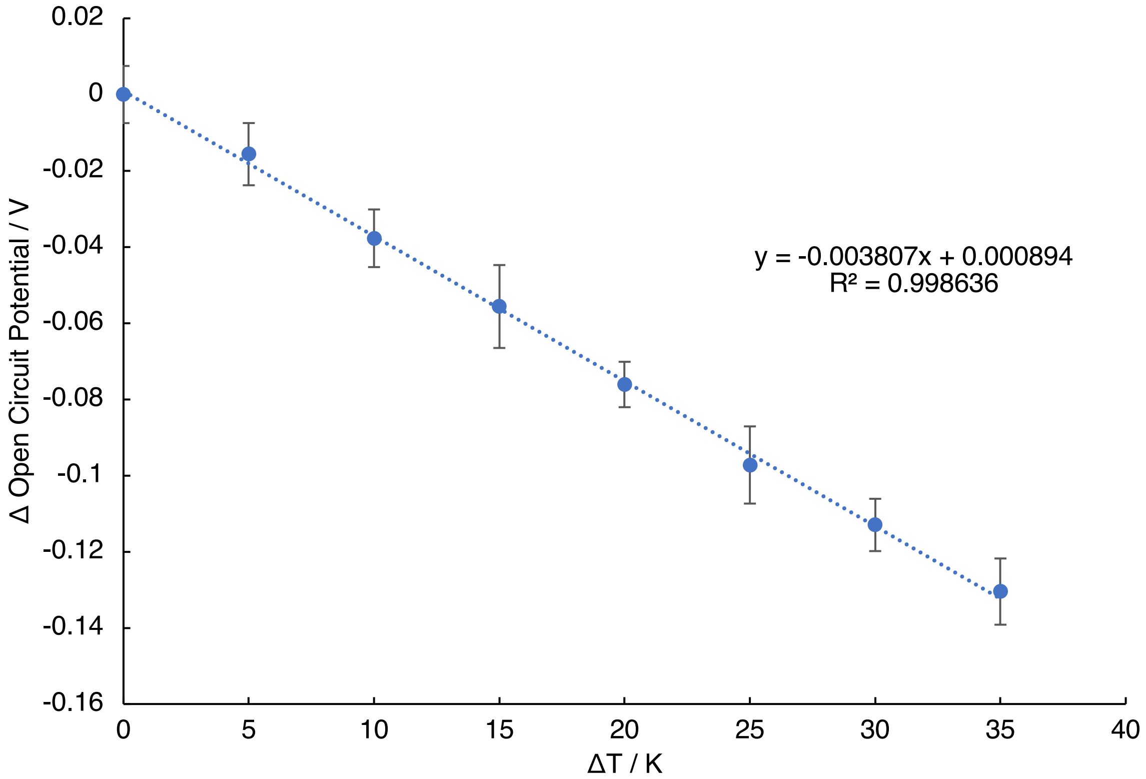

Figure S26: van't Hoff plot of 2 mM In_4L_6 + 2 mM $CcCO_2$

Table S14: Data for van't Hoff plot of 2 mM In_4L_6 + 2 mM $CcCO_2$

	Open Circuit Potential (V)		
ΔT (K)	Run 1	Run 2	Run 3
0	0	0	0
5	-0.01435	-0.01534	-0.01727
10	-0.0343	-0.0401	-0.0388
15	-0.05112	-0.05281	-0.06301
20	-0.07669	-0.07847	-0.07319
25	-0.09951	-0.09146	-0.10078
30	-0.11762	-0.11189	-0.10946
35	-0.12919	-0.12923	-0.13303

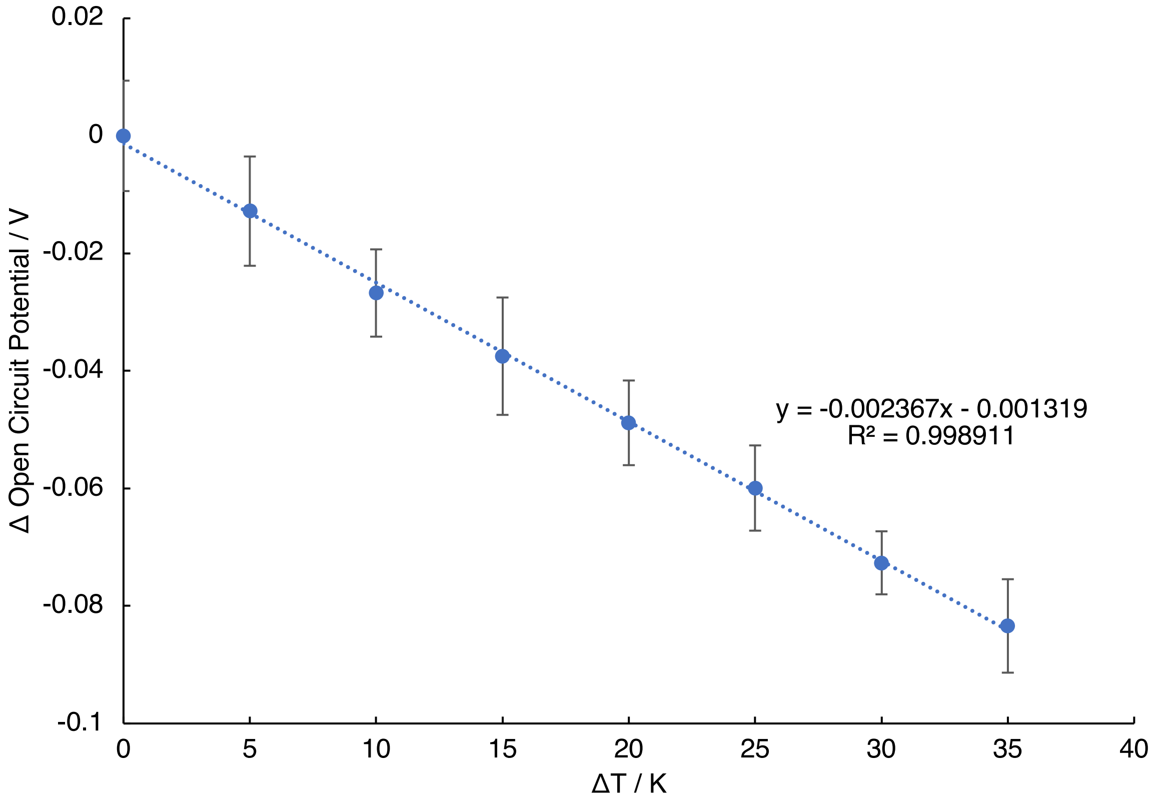

Figure S27: van't Hoff plot of 2 mM Ge_4L_6 + 2 mM $CcCO_2$

Table S15: Data for van't Hoff plot of 2 mM Ge_4L_6 + 2 mM $CcCO_2$

	Open Circuit Potential (V)		
ΔT (K)	Run 1	Run 2	Run 3
0	0	0	0
5	-0.01784	-0.00903	-0.01156
10	-0.03314	-0.02537	-0.02171
15	-0.04072	-0.03374	-0.03802
20	-0.0505	-0.05152	-0.04448
25	-0.06903	-0.05742	-0.05334
30	-0.07792	-0.07571	-0.06434
35	-0.08801	-0.08229	-0.0799

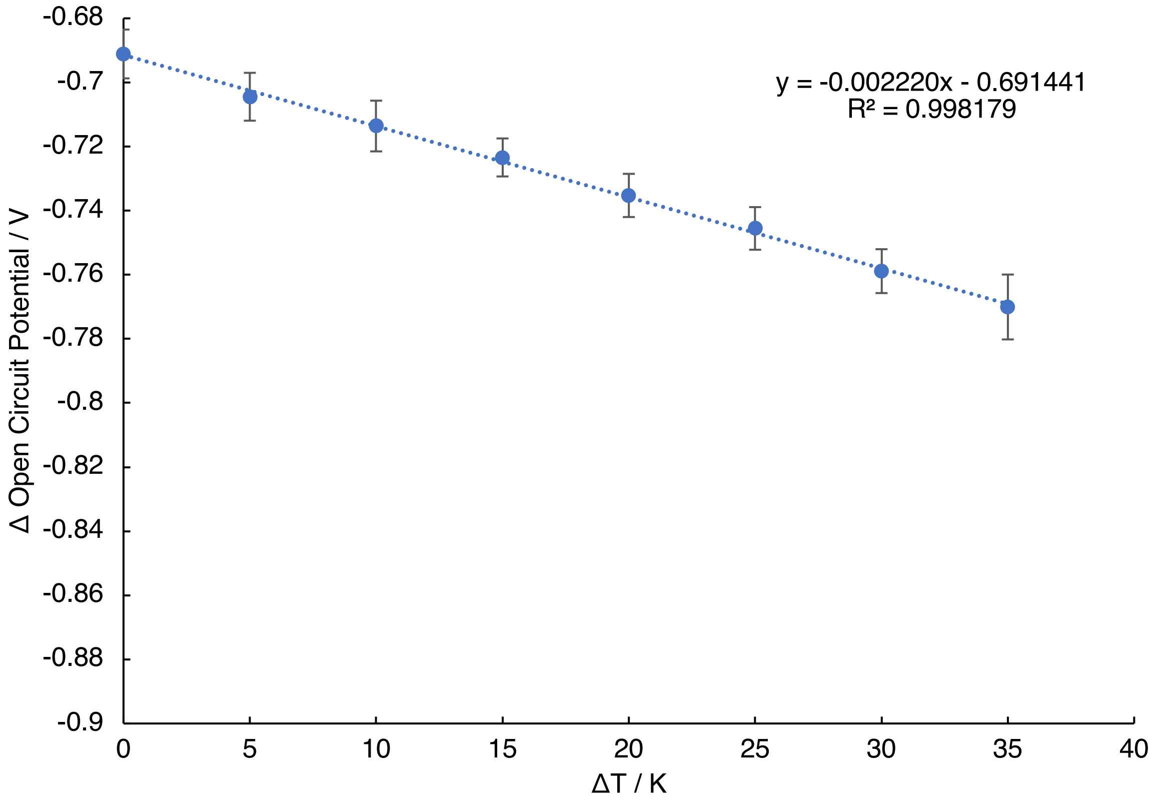

Figure S28: van't Hoff plot of 2 mM Si_4L_6 + 2 mM $CcCO_2$

Table S16: Data for van't Hoff plot of 2 mM Si_4L_6 + 2 mM $CcCO_2$

	Open Circuit Potential (V)		
ΔT (K)	Run 1	Run 2	Run 3
0	0	0	0
5	-0.01803	-0.00757	-0.01446
10	-0.02425	-0.01909	-0.02411
15	-0.03503	-0.03261	-0.02922
20	-0.04634	-0.04305	-0.04302
25	-0.05739	-0.05248	-0.05342
30	-0.06685	-0.07319	-0.06323
35	-0.07429	-0.07948	-0.08305

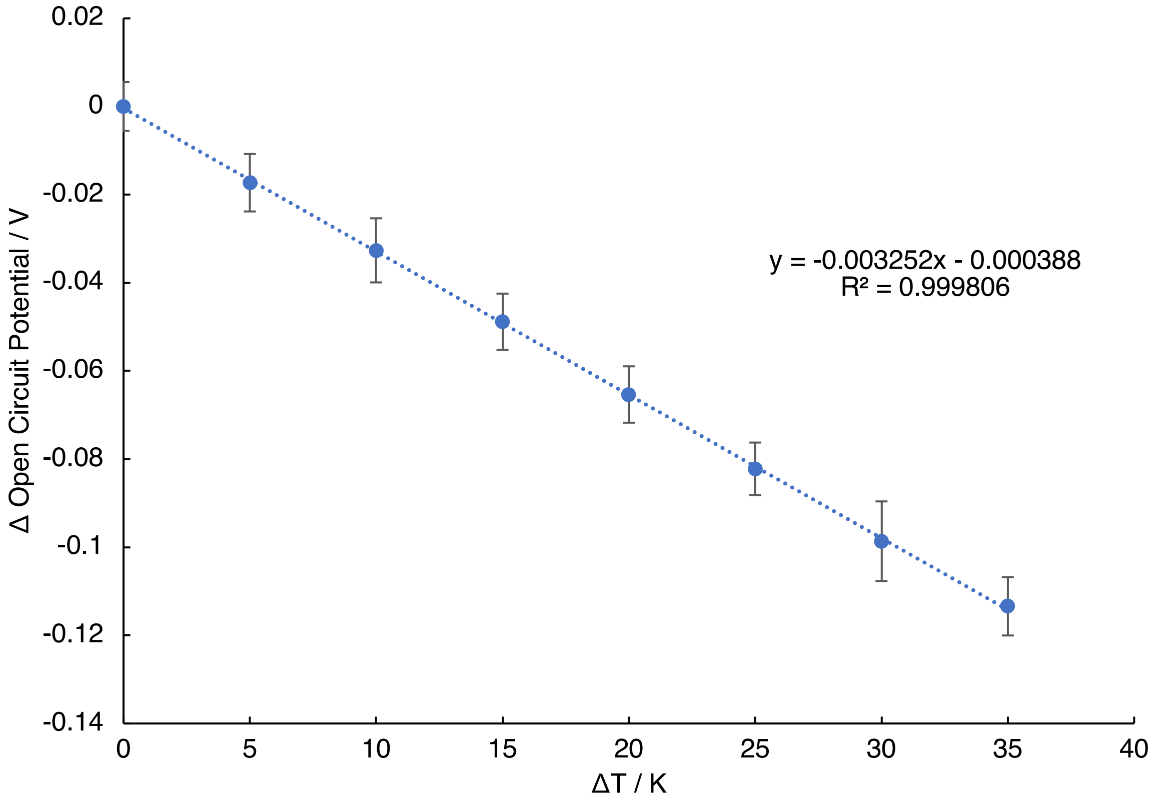

Figure S29: van't Hoff plot of 2 mM Ga-**2** + 2 mM $CcCO_2$

Table S17: Data for van't Hoff plot of 2 mM Ga-**2** + 2 mM $CcCO_2$

	Open Circuit Potential (V)		
ΔT (K)	Run 1	Run 2	Run 3
0	0	0	0
5	-0.02006	-0.01447	-0.01736
10	-0.03748	-0.02793	-0.03252
15	-0.04676	-0.04859	-0.05112
20	-0.06920	-0.06246	-0.06440
25	-0.08075	-0.08232	-0.08356
30	-0.10140	-0.09140	-0.10311
35	-0.11545	-0.11066	-0.11412

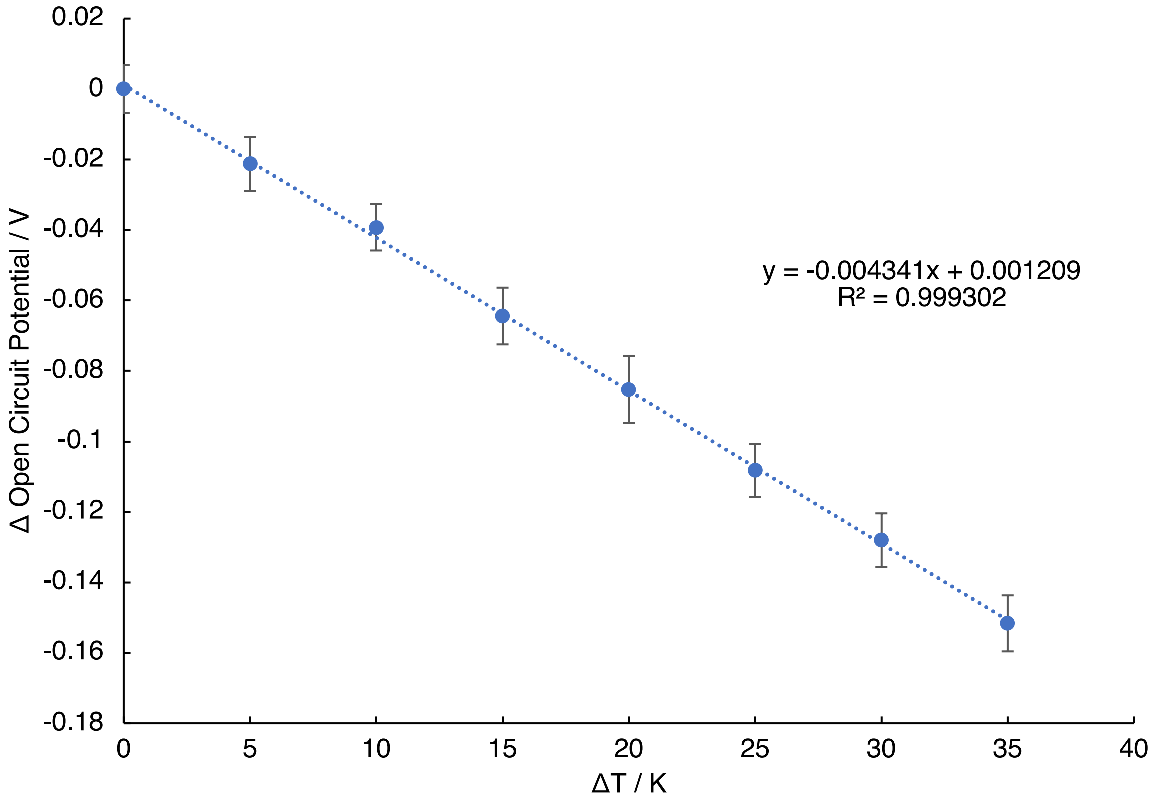

Figure S30: van't Hoff plot of 2 mM Ga-**3** + 2 mM $CcCO_2$

Table S18: Data for van't Hoff plot of 2 mM Ga-**3** + 2 mM $CcCO_2$

	Open Circuit Potential (V)		
ΔT (K)	Run 1	Run 2	Run 3
0	0	0	0
5	-0.01936	-0.02831	-0.01609
10	-0.03938	-0.04109	-0.03728
15	-0.06157	-0.0676	-0.06407
20	-0.07934	-0.09058	-0.08578
25	-0.10646	-0.11243	-0.10585
30	-0.12599	-0.13172	-0.12641
35	-0.14982	-0.15118	-0.15393

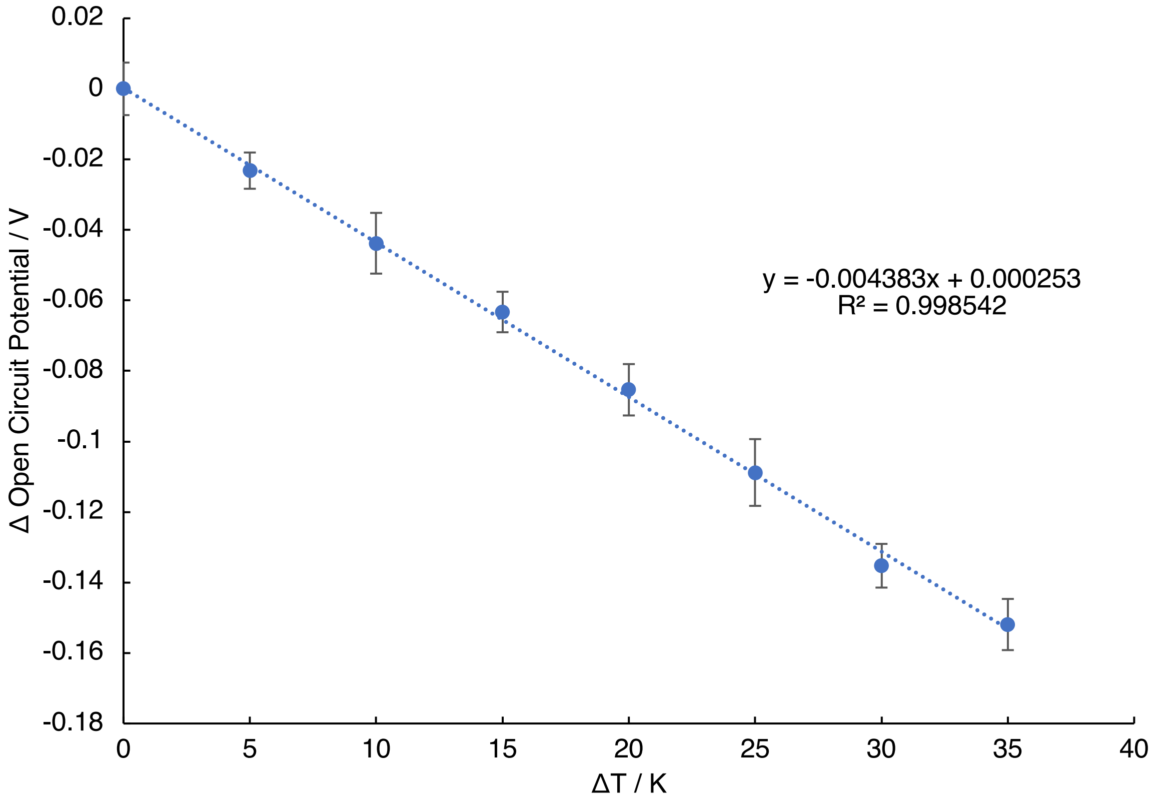

Figure S31: van't Hoff plot of 2 mM In-**3** + 2 mM $CcCO_2$ in 1 M pH 12 sodium phosphate buffer

Table S19: Data for van't Hoff plot of 2 mM In-**3** + 2 mM $CcCO_2$ in 1 M pH 12 sodium phosphate buffer

	Open Circuit Potential (V)		
ΔT (K)	Run 1	Run 2	Run 3
0	0	0	0
5	-0.02687	-0.02382	-0.01889
10	-0.04283	-0.04197	-0.04657
15	-0.06622	-0.06335	-0.0603
20	-0.0842	-0.08859	-0.08326
25	-0.10516	-0.10911	-0.11216
30	-0.13579	-0.13932	-0.13066
35	-0.15088	-0.15501	-0.14989

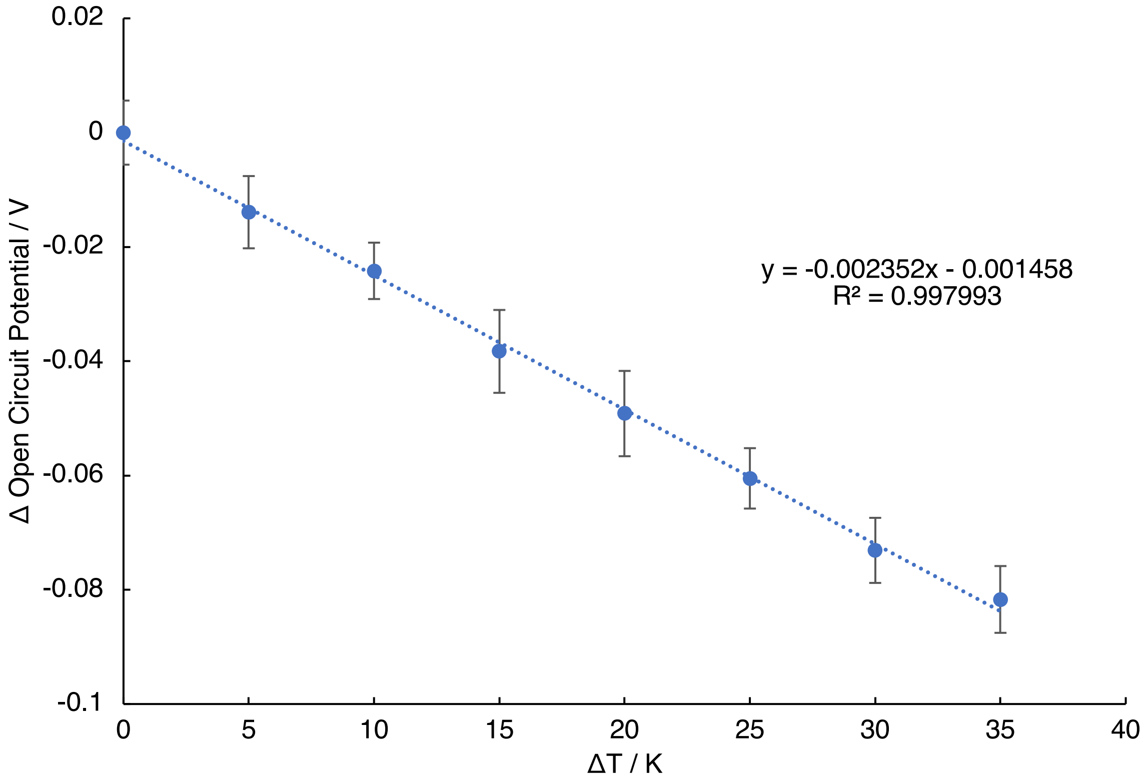

Figure S32: van't Hoff plot of 0.1 M Fe_4L_6 + 0.1 M $CcCO_2$

Table S20: Data for van't Hoff plot of 0.1 M Fe_4L_6 + 0.1 M $CcCO_2$

	Open Circuit Potential (V)		
ΔT (K)	Run 1	Run 2	Run 3
0	0	0	0
5	-0.01494	-0.01451	-0.01232
10	-0.02293	-0.02440	-0.02524
15	-0.04013	-0.04053	-0.03420
20	-0.04906	-0.05506	-0.04338
25	-0.06031	-0.05925	-0.06196
30	-0.06990	-0.07885	-0.07058
35	-0.07880	-0.08739	-0.07890

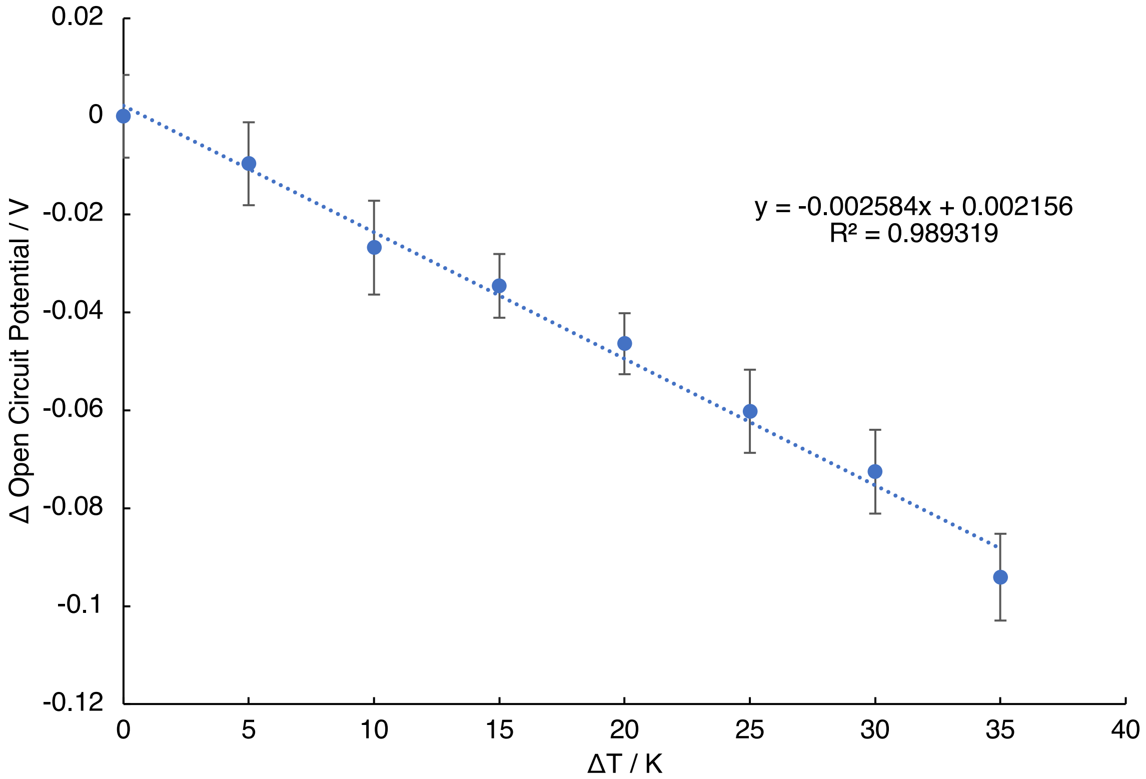

Figure S33: van't Hoff plot of 0.1 M Ga_4L_6 + 0.1 M $CcCO_2$

Table S21: Data for van't Hoff plot of 0.1 M Ga_4L_6 + 0.1 M $CcCO_2$

	Open Circuit Potential (V)		
ΔT (K)	Run 1	Run 2	Run 3
0	0	0	0
5	-0.00698	-0.00684	-0.01524
10	-0.02166	-0.02488	-0.03392
15	-0.03659	-0.03016	-0.03712
20	-0.04726	-0.03747	-0.05457
25	-0.05755	-0.05755	-0.06558
30	-0.06973	-0.06993	-0.07803
35	-0.09061	-0.09176	-0.09992

Table S22: Summary of Electrochemical van't Hoff Data

	mV/K	S
$CcCO_2$ only	–1.87(9)	–43(2)
Ga_4L_6 only	–0.41(3)	–9.4(7)
$CcCO_2$ + Ga_4L_6 + PEt_4^+	–2.00(4)	–46.0(9)
pH 12 K phosphate buffer	–0.119(4)	–2.74(9)
$CcCO_2$ + Ga_4L_6	–3.62(4)	–83.5(9)
$CcCO_2$ + Ga_4L_6 in Na buffer	–3.97(3)	–91.6(7)
$CcCO_2$ + Fe_4L_6	–3.28(6)	–75(1)
$CcCO_2$ + In_4L_6	–3.81(5)	–88(1)
$CcCO_2$ + Ge_4L_6	–2.37(8)	–55(2)
$CcCO_2$ + Si_4L_6	–2.22(8)	–52(2)
$CcCO_2$ + Ga-**2**	–3.28(6)	–76(1)
$CcCO_2$ + Ga-**3**	–4.34(4)	–100.1(9)
$CcCO_2$ + In-**3** in Na buffer	–4.38(6)	–101(1)
0.1 M $CcCO_2$ + 0.1 M Fe_4L_6	–2.58(5)	–60(1)
0.1 M $CcCO_2$ + 0.1 M Ga_4L_6	–2.35(8)	–54(2)

Validation of Electrochemical van't Hoff Method

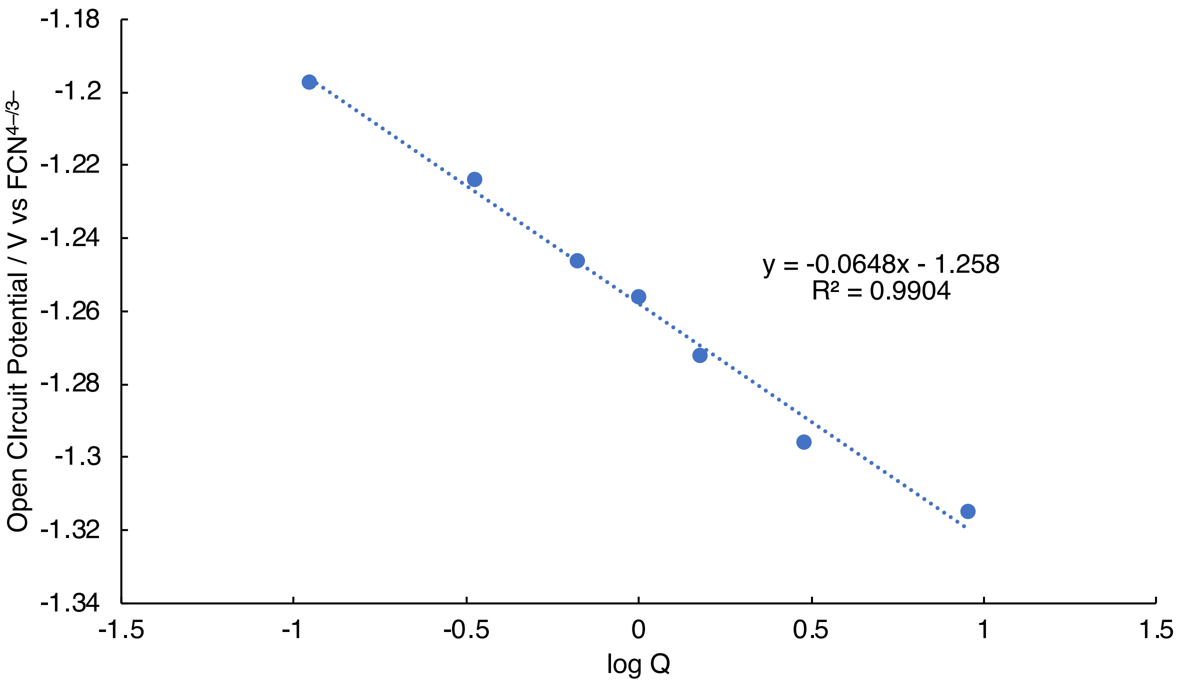

Figure S34: Open circuit potential versus log Q for 2 mM $CcCO_2$

1 M pH 12 potassium phosphate buffer in water

Table S23: Data for Figure S28

log Q	OCP (V vs. $FCN^{4-/3-}$)
0.95424251	-1.315
0.47712125	-1.296
0.17609126	-1.272
0	-1.256
-0.1760913	-1.246
-0.4771213	-1.224
-0.9542425	-1.197

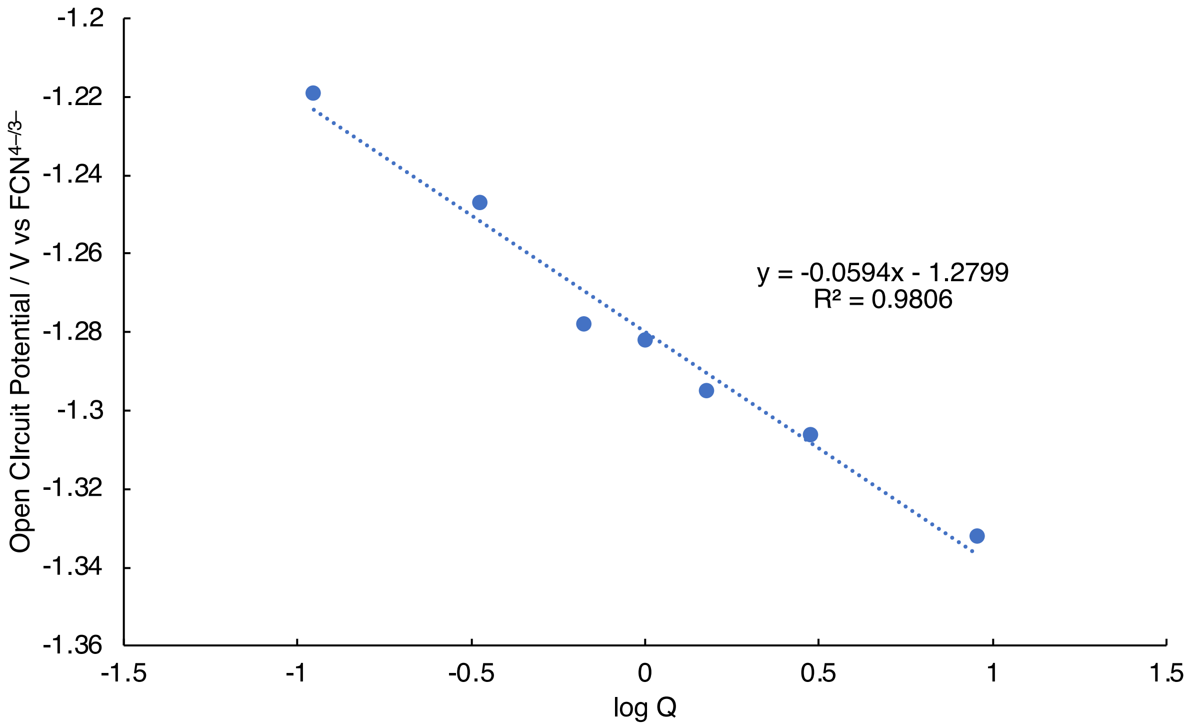

Figure S35: Open circuit potential versus log Q for 2 mM $CcCO_2$ + 2 mM Ga_4L_6

1 M pH 12 potassium phosphate buffer in water

Table S24: Data for Figure S28

log Q	OCP (V vs. $FCN^{4-/3-}$)
0.95424251	-1.332
0.47712125	-1.306
0.17609126	-1.295
0	-1.282
-0.1760913	-1.278
-0.4771213	-1.247
-0.9542425	-1.219

Device Testing Data

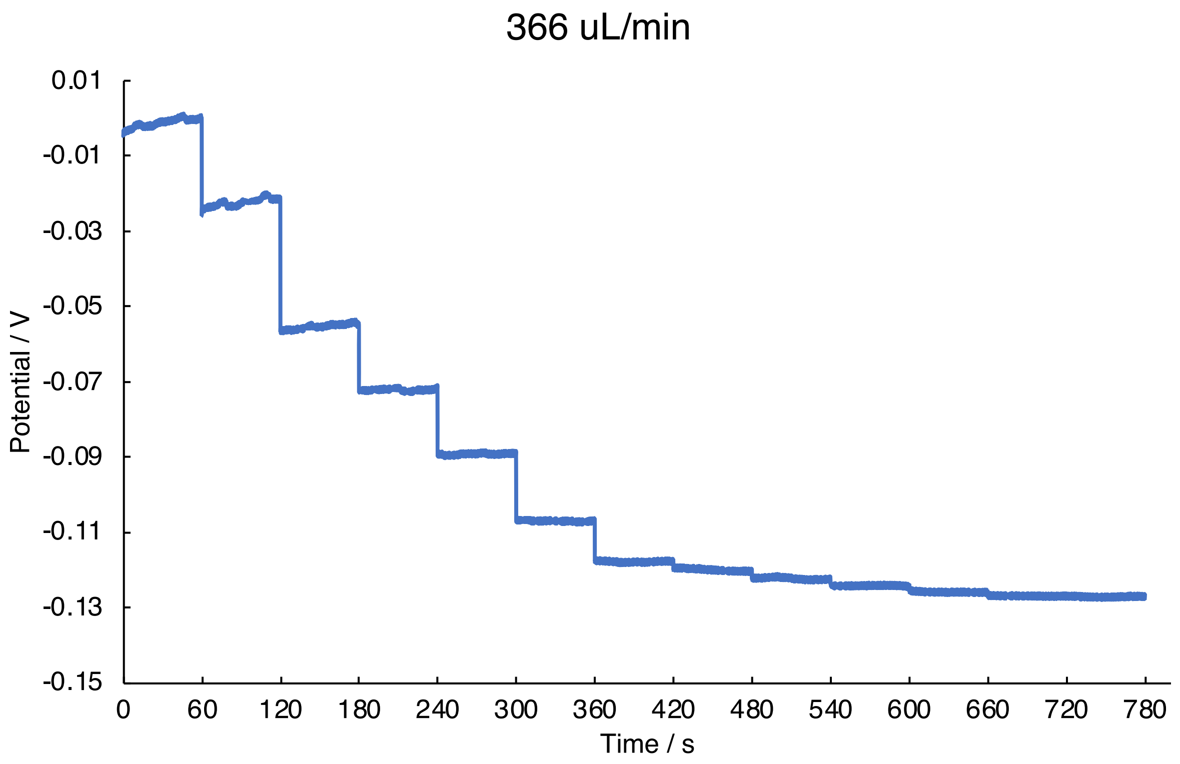

Figure S36: Potential vs Time for Host-Guest System at 366 μL min^{-1} flow rate

Table S25: Current steps for experiment in Figure S31

Step	Current (A)	Duration (s)
1	0.00032	60
2	0.0003	60
3	0.0002	60
4	0.00015	60
5	0.0001	60
6	0.00005	60
7	0.00002	60
8	0.000015	60
9	0.00001	60
10	0.000005	60
11	0.000002	60
12	0	60

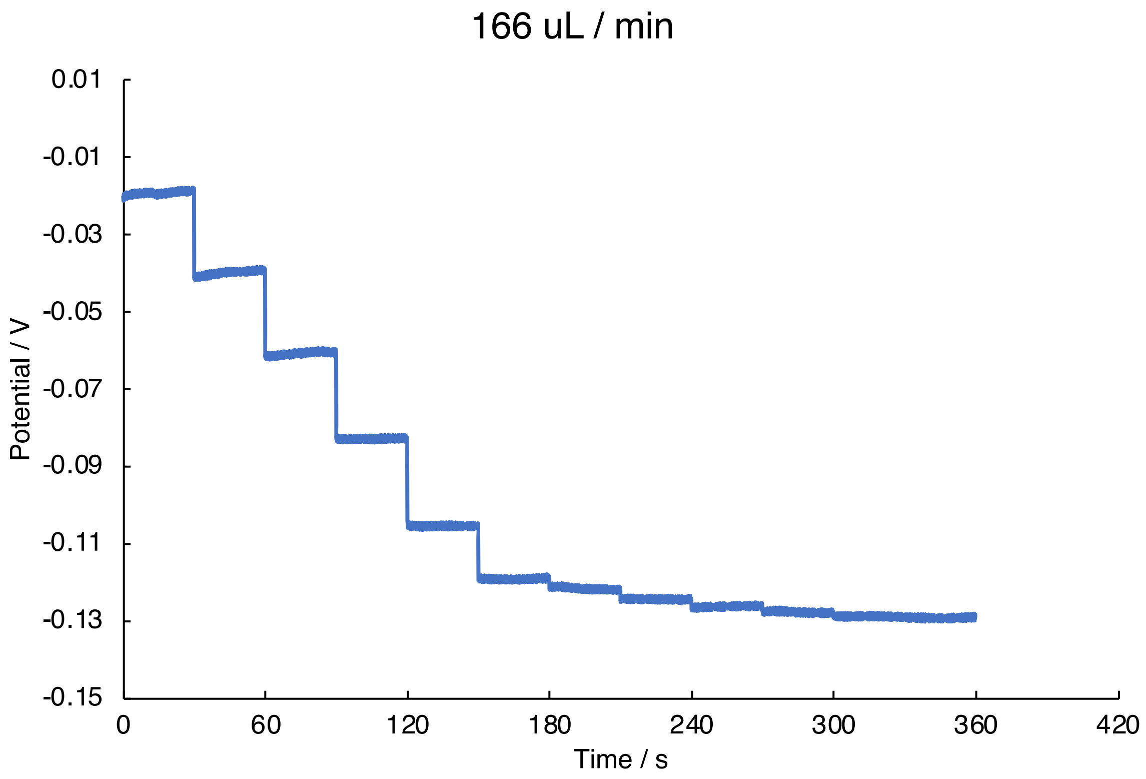

Figure S37: Potential vs Time for Host-Guest System at 166 μL min^{-1} flow rate

Table S26: Current steps for experiment in Figure S32

Step	Current (A)	Duration (s)
1	0.00025	30
2	0.0002	30
3	0.00015	30
4	0.0001	30
5	0.00005	30
6	0.00002	30
7	0.000015	30
8	0.00001	30
9	0.000005	30
10	0.000002	30
11	0	30

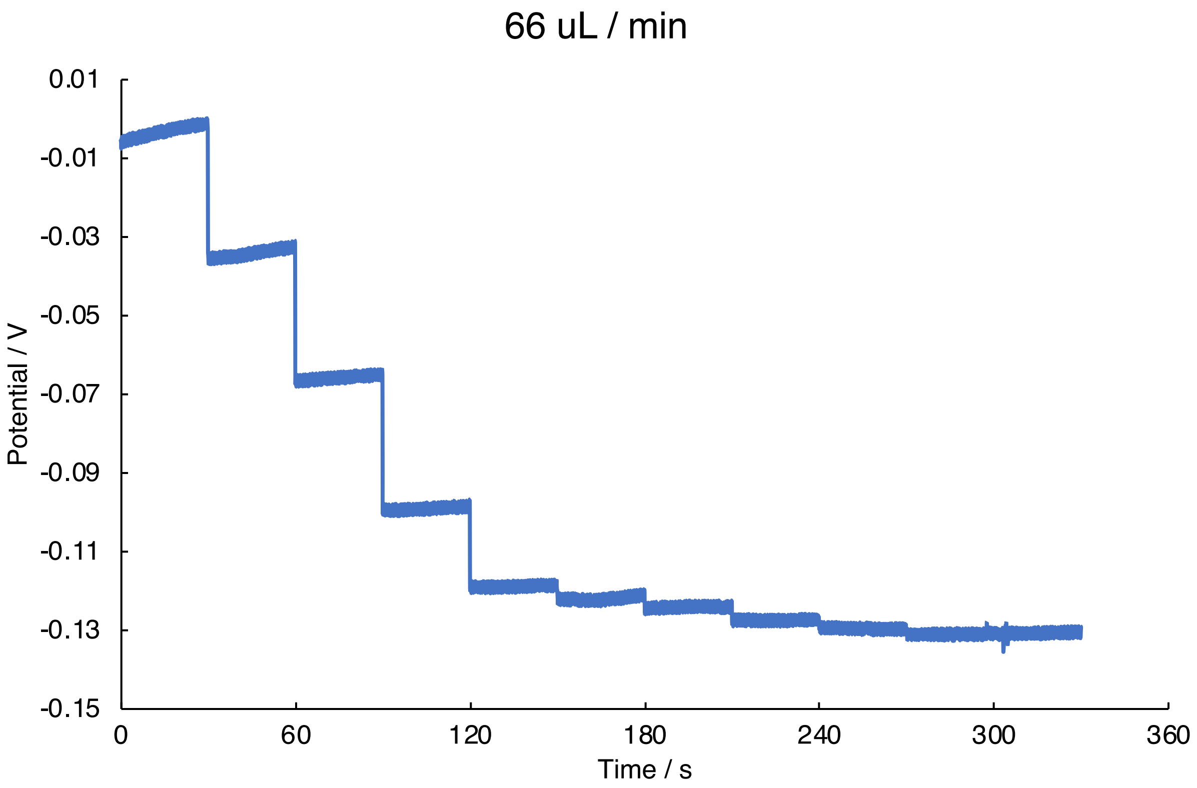

Figure S38: Potential vs Time for Host-Guest System at 66 μL min^{-1} flow rate

Table S27: Current steps for experiment in Figure S33

Step	Current (A)	Duration (s)
1	0.0002	30
2	0.00015	30
3	0.0001	30
4	0.00005	30
5	0.00002	30
6	0.000015	30
7	0.00001	30
8	0.000005	30
9	0.000002	30
10	0	30

Table S28: Summary of Data for Host-Guest System

	Potential (mV)			Power (μW cm^{-2})		
I (mA cm^{-2})	66 μL/min	166 μL/min	366 μL/min	66 μL/min	166 μL/min	366 μL/min
0	129	130	129	0	0	0
0.0007843	129	129	127	0.1012	0.1012	0.0996
0.0019608	128	128	125	0.2510	0.2510	0.2451
0.0039216	125	126	123	0.4902	0.4941	0.4824
0.0058824	122	123	120	0.7176	0.7235	0.7059
0.0078431	118	120	118	0.9255	0.9412	0.9255
0.0196078	98	106	107	1.9216	2.0784	2.0980
0.0392157	65	82	88	2.5490	3.2157	3.4510
0.0588235	32	58	71	1.8824	3.4118	4.1765
0.0784314	1	36	53	0.0784	2.8235	4.1569
0.0980392		14			1.3725	
0.1176471			19			2.2353
0.1254902			3			0.3765

Table S29: Summary of Data for Ferri/ferrocyanide System

	Potential (mV)			Power (μW cm^{-2})		
I (mA cm^{-2})	66 μL/min	166 μL/min	366 μL/min	66 μL/min	166 μL/min	366 μL/min
0	78	78	78	0	0	0
0.0039216	77	77	78	0.3020	0.3020	0.3059
0.0098039	76	77	77	0.7451	0.7549	0.7549
0.0196078	73	74	75	1.4314	1.4510	1.4706
0.0294118	70	72	74	2.0588	2.1176	2.1765
0.0392157	68	70	72	2.6667	2.7451	2.8235
0.0980392	54	59	62	5.2941	5.7843	6.0784
0.1960784	29	40	46	5.6863	7.8431	9.0196
0.2941176	2	21	29	0.5882	6.1765	8.5294
0.3921569		1	14		0.3922	5.4902
0.4313725			8			3.4510

Table S30: Comparison of Power and Efficiency

Flow rate (μL min^{-1})	66	166	366
Efficiency (FCN)<br>(% of Carnot Efficiency)	12%	7%	4%
Power (FCN)<br>(μW cm^{-2})	6.1	7.9	9.0
Efficiency (host/guest)<br>(% of Carnot Efficiency)	6%	3%	2%
Power (host/guest)<br>(μW cm^{-2})	2.5	3.5	4.3

Table S31: Comparison of Figures of Merit

Figure of merit "Y" is defined as:

$$Y = \frac{\alpha n F C_{sol}}{C_p}$$

Parameter	Description	Fe_4L_6 system	FCN system
α	Temperature coefficient	–2.35 mV K^{-1}	–0.93 mV K^{-1}
n	Electrons transferred	1	1
C_{sol}	Solubility	0.1 M	0.6 M
C_p	Heat capacity	3.1 J K^{-1} g^{-1}	3.5 J K^{-1} g^{-1}
**Y**	**Figure of merit**	**–0.015**	**–0.031**

Calculation for Power and Efficiency with Increased Solubility

The maximum power always occurs at half of the maximum current density, which also always corresponds to half of the open circuit potential, 64 mV. Assuming that current density is directly proportional to the solubility, and that all other parameters are independent, we can perform a simple calculation to determine at what solubility the host-guest system can achieve a higher power density and efficiency than potassium ferri/ferrocyanide.

The power density (P) is simple: $P = I \times V$. So if current density is proportional to solubility, then so is power density. The maximum power density at 0.1 M concentration for the host-guest system is 4.3 μW cm^{-2}, and the power density of potassium ferri/ferrocyanide is 9.0 μW cm^{-2}, approximately double. We can expect the host-guest system to surpass ferri/ferrocyanide at higher than approximately double the present solubility, 0.21 M.

For the efficiency, we refer back to the equation described earlier for calculating the efficiency of the device:

$$\eta = \frac{Power\ output}{Power\ input} = \frac{I \times V}{I \times T_{hot}(\alpha_1 - \alpha_2) + \left(Q_1 C_{p1} \rho_1 + Q_2 C_{p2} \rho_2\right) \times (T_{hot} - T_{cold})}$$

If we assume that all the values except for current density stay constant, this gives us the following equation for efficiency (η) as a function of current density (*I*) and flow rate (*Q*), where A and B are constants:

$$\eta = \frac{I \times V}{I \times A + Q \times B}$$

With a hot side at 50 °C and a cold side at 10 °C, Carnot efficiency is η_c = 12.4%. We can divide the efficiency η by 12.4% to obtain the efficiency as a percentage of Carnot efficiency.

At a flow rate of 366 μL min^{-1}, the efficiency of potassium ferri/ferrocyanide is 12% of Carnot efficiency. Solving the equation above, we find that *I* must increase by a factor of 1.7 for the efficiency of the host-guest system to match that of potassium ferri/ferrocyanide. So the host-guest system will have a higher efficiency at concentrations of 0.17 M or higher.

Solving this equation for different flow rates, we find that at 166 μL min^{-1}, the host-guest system will have a higher efficiency than potassium ferri/ferrocyanide at higher than 0.21 M, and at 66 μL min^{-1}, the host-guest system will have a higher efficiency than potassium ferri/ferrocyanide at higher than 0.23 M.

Other Characterization Data

Solubility

Table S32: Solubility of various compounds

	Solubility (M)
Ga_4L_6 in 1 M pH 12 potassium phosphate buffer	0.124(3)
Fe_4L_6 in 1 M pH 12 potassium phosphate buffer	0.108(2)
$CcCO_2$ in 1 M pH 12 potassium phosphate buffer	0.21(1)

Density

Table S33: Density of electrolyte solutions

	Density (mg cm^{-3})
0.1 M Fe_4L_6 + 0.1 M $CcCO_2$ + 1 M pH 12 potassium phosphate buffer	1226(2)
1.2 M KI + 0.01 M I_2 + 1 M pH 12 potassium phosphate buffer	1292(3)
0.3 M $K_3Fe(CN)_6$ + 0.3 M $K_4Fe(CN)_6$	1283(2)
2.1 M KI + 0.15 M I_2	1195(4)

Heat Capacity

Table S34: Heat Capacity of electrolyte solutions

	Heat Capacity (J K^{-1} g^{-1})
0.1 M Fe_4L_6 + 0.1 M $CcCO_2$ + 1 M pH 12 potassium phosphate buffer	3.12
1.2 M KI + 0.01 M I_2 + 1 M pH 12 potassium phosphate buffer	3.12
0.3 M $K_3Fe(CN)_6$ + 0.3 M $K_4Fe(CN)_6$	3.63
2.1 M KI + 0.15 M I_2	3.05

^{1}H NMR Spectra

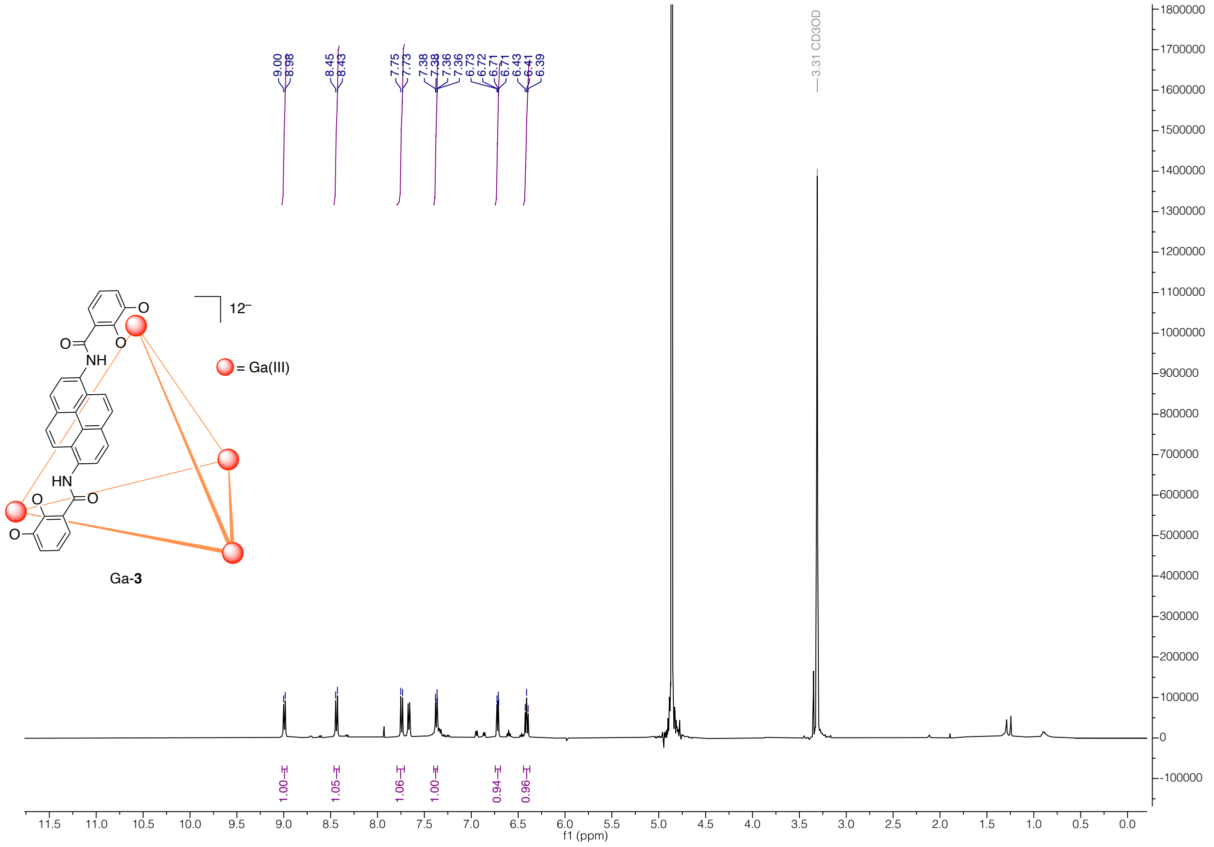

Figure S39: ^{1}H NMR spectrum of Ga-**3**

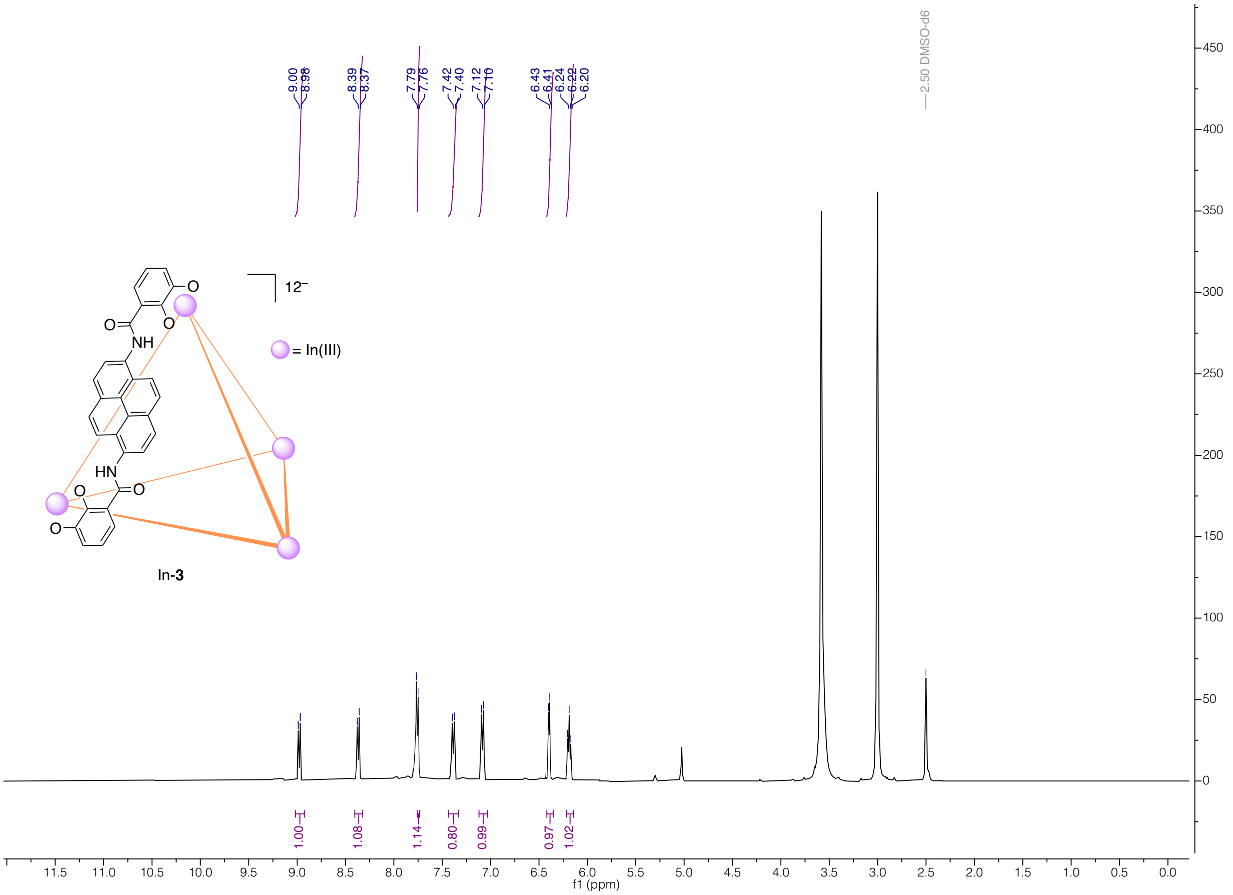

Figure S40: ^{1}H NMR spectrum of In-**3**

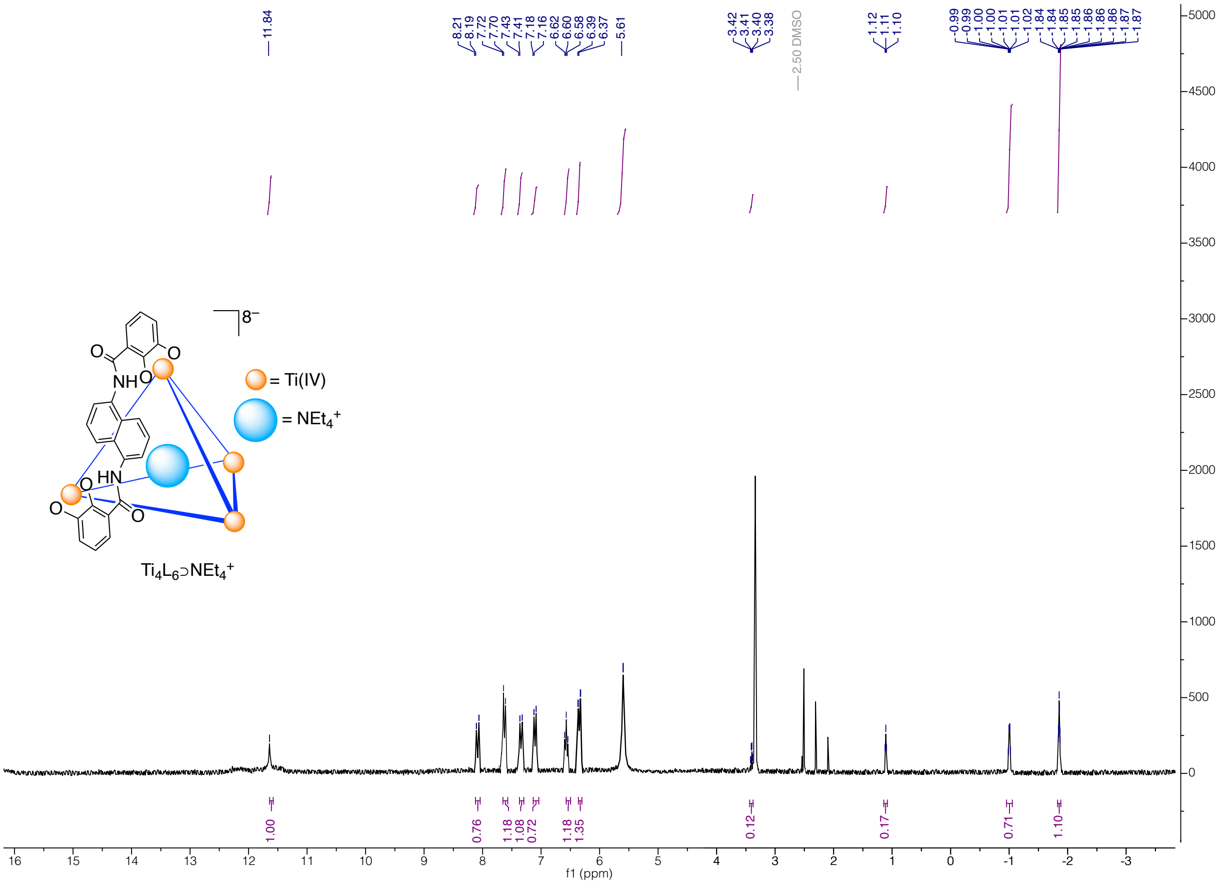

Figure S41: ^{1}H NMR spectrum of $NEt_4^+ \subset Ti_4L_6$

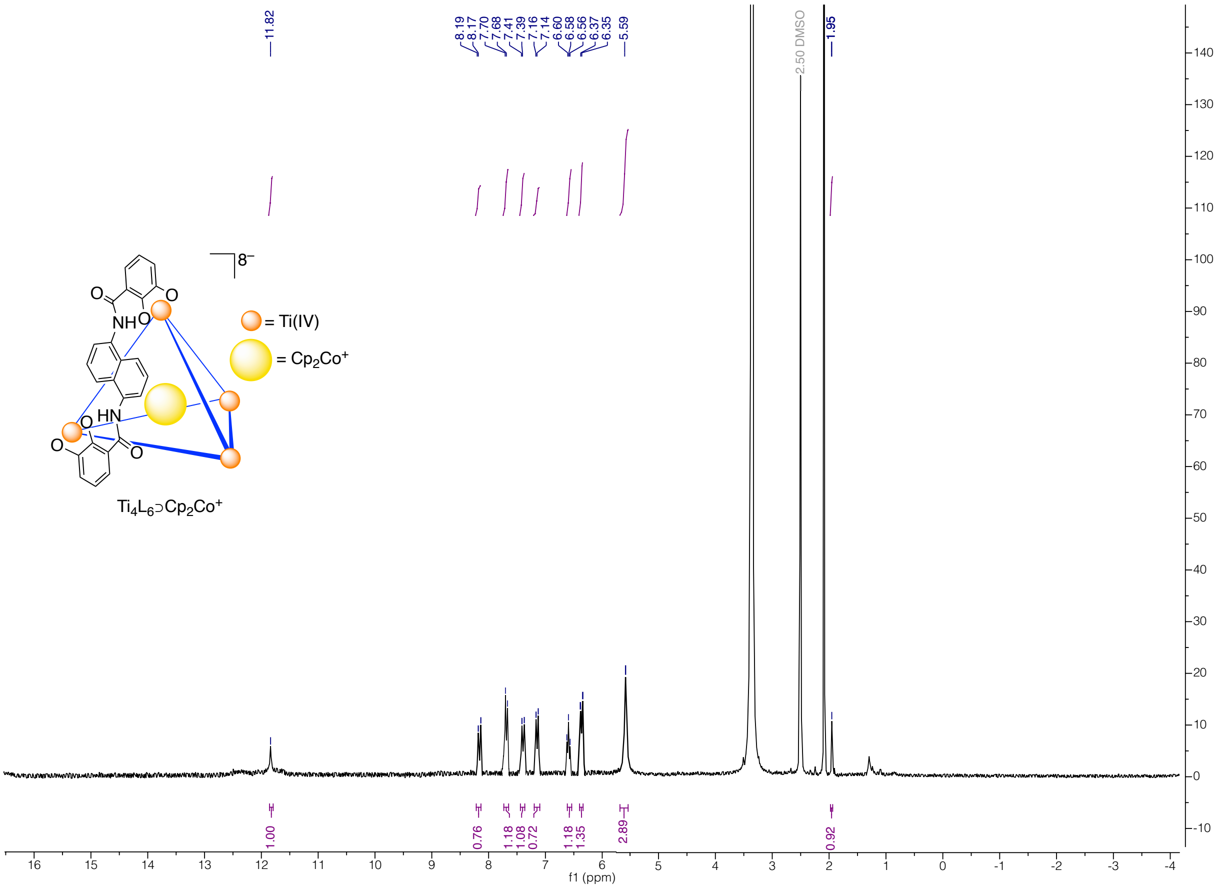

Figure S42: ^{1}H NMR spectrum of $Cc^+ \subset Ti_4L_6$

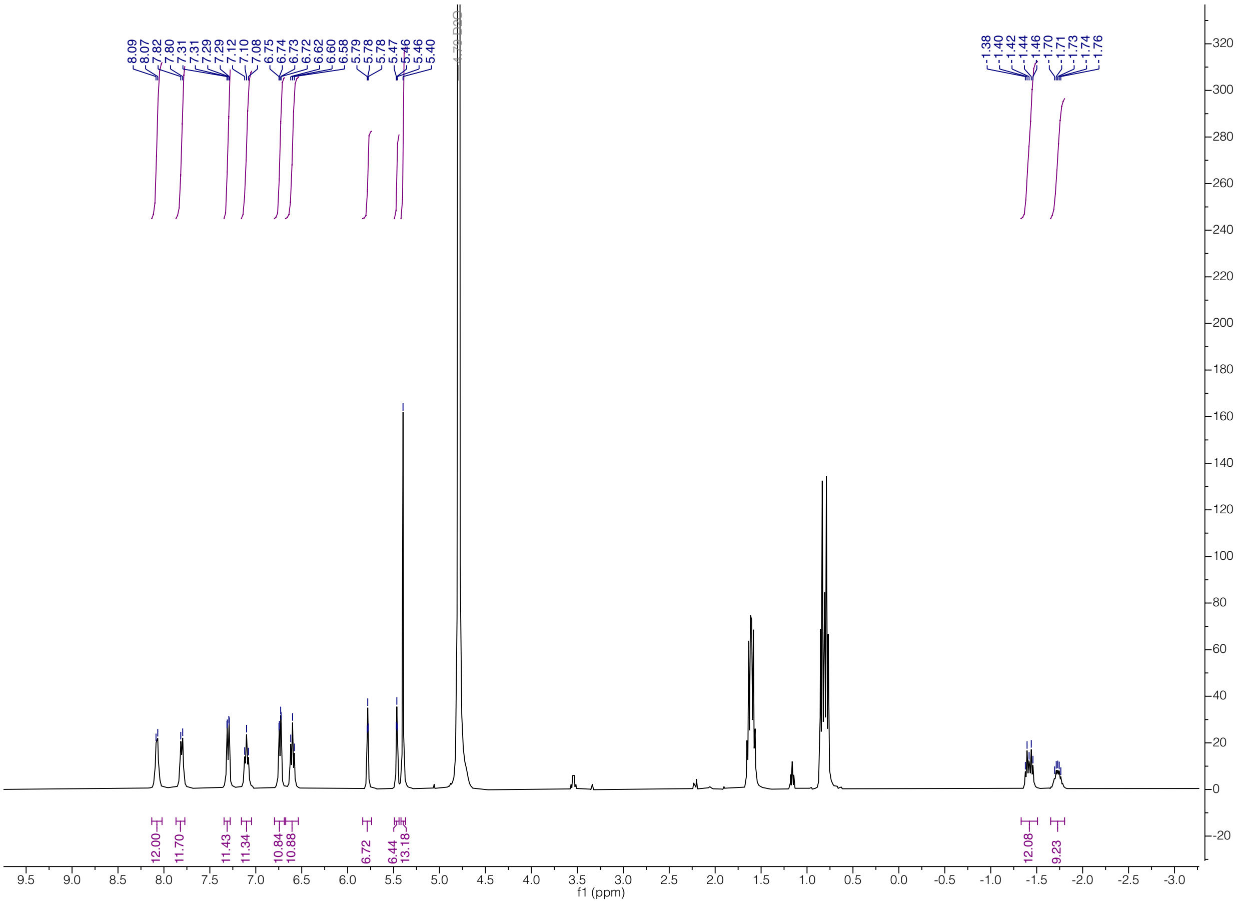

Figure S43: ^{1}H NMR spectrum of Ga_4L_6 and $CcCO_2HPF_6$ and PEt_4I

100 mM potassium phosphate buffer in D_2O at pD = 12

Ga_4L_6 peaks appear between 6.5 to 8.5 ppm, $CcCO_2$ peaks appear between 5 to 6 ppm, and are not upfield shifted, implying they are external to the host cavity. Peaks corresponding to PEt_4^+ external to the host cavity appear between 0.5 to 2 ppm, and peaks corresponding to PEt_4^+ encapsulated within the host cavity appear between –1 to –2 ppm, showing the distinctive upfield shift that implies encapsulation, and integrating to 1 equivalent relative to Ga_4L_6. These data show that PEt_4^+ strongly outcompetes $CcCO_2$ to bind in the host cavity.

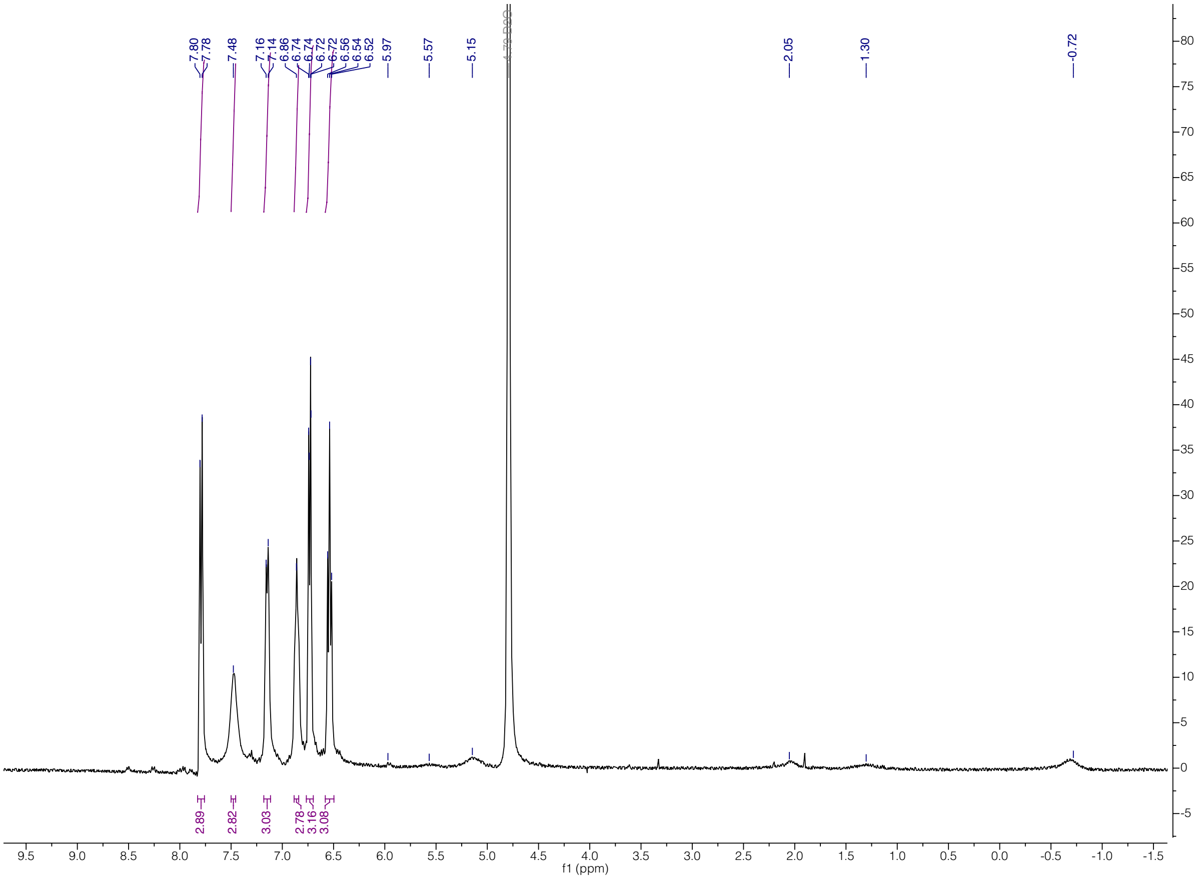

Figure S44: ^{1}H NMR spectrum of Ga_4L_6 and $CcCO_2HPF_6$

Solvent: D_2O with 1M potassium phosphate buffer, pD = 12

Peaks corresponding to $CcCO_2$ external to the host cavity appear at 5.97 ppm, 5.57 ppm, and 5.15 ppm, and are significantly broadened. Peaks corresponding to $CcCO_2$ inside the host cavity are upfield shifted and appear at 2.05 ppm, 1.30 ppm, and –0.72 ppm.

The peaks corresponding to $CcCO_2$ are likely broadened due to restricted rotation within the host cavity and when ion-paired to the exterior of the host. This phenomena has previously been reported for asymmetrical phosphonium guests bound within the Ga_4L_6 host.[13]

UV-vis Spectra

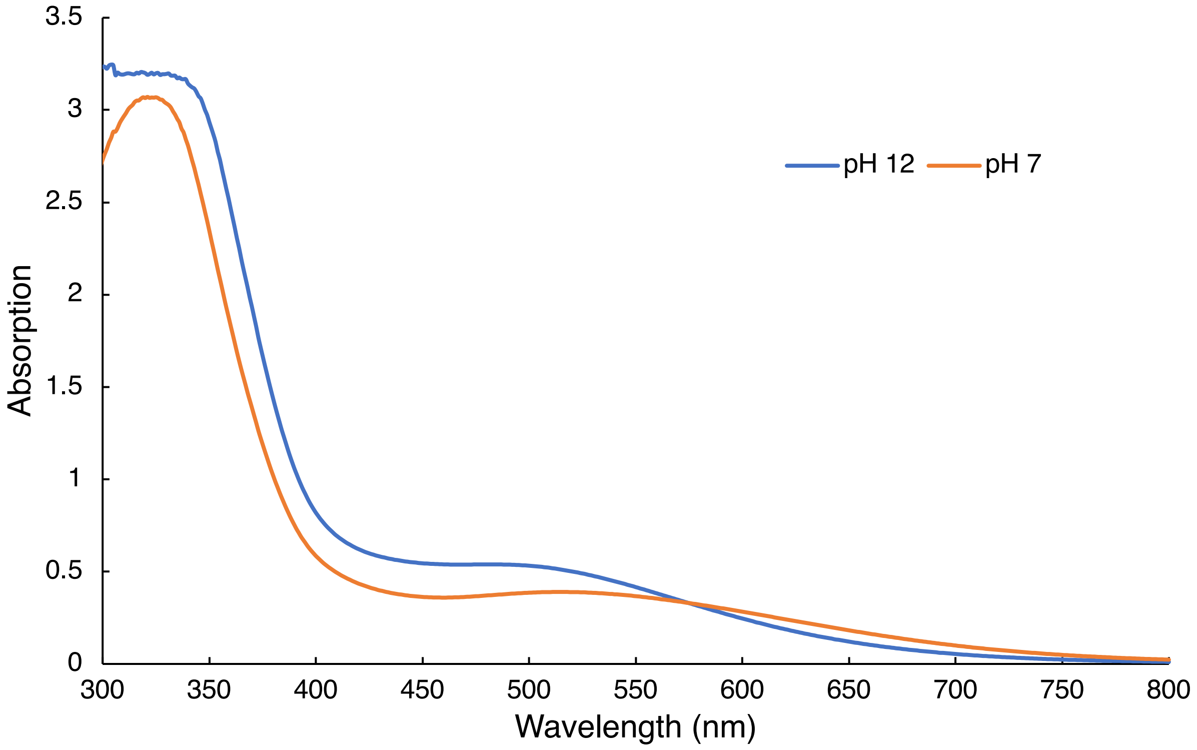

Figure S45: UV-vis spectrum of Fe_4L_6

References

(1) Fulmer, G. R.; Miller, A. J. M.; Sherden, N. H.; Gottlieb, H. E.; Nudelman, A.; Stoltz, B. M.; Bercaw, J. E.; Goldberg, K. I. NMR Chemical Shifts of Trace Impurities: Common Laboratory Solvents, Organics, and Gases in Deuterated Solvents Relevant to the Organometallic Chemist. *Organometallics* **2010**, *29* (9), 2176–2179. https://doi.org/10.1021/om100106e.

(2) Nguyen, Q. N. N.; Xia, K. T.; Zhang, Y.; Chen, N.; Morimoto, M.; Pei, X.; Ha, Y.; Guo, J.; Yang, W.; Wang, L. P.; Bergman, R. G.; Raymond, K. N.; Toste, F. D.; Tantillo, D. J. Source of Rate Acceleration for Carbocation Cyclization in Biomimetic Supramolecular Cages. *J. Am. Chem. Soc.* **2022**, *144* (25), 11413–11424. https://doi.org/10.1021/jacs.2c04179.

(3) Sokolow, G. E.; Crawley, M. R.; Morphet, D. R.; Asik, D.; Spernyak, J. A.; McGray, A. J. R.; Cook, T. R.; Morrow, J. R. Metal-Organic Polyhedron with Four Fe(III) Centers Producing Enhanced T1Magnetic Resonance Imaging Contrast in Tumors. *Inorg. Chem.* **2022**, *61* (5), 2603–2611. https://doi.org/10.1021/acs.inorgchem.1c03660.

(4) Pluth, M. D.; Johnson, D. W.; Szigethy, G.; Davis, A. V.; Teat, S. J.; Oliver, A. G.; Bergman, R. G.; Raymond, K. N. Structural Consequences of Anionic Host-Cationic Guest Interactions in a Supramolecular Assembly. *Inorg. Chem.* **2009**, *48* (1), 111–120. https://doi.org/10.1021/ic8012848.

(5) Bierschenk, S. M.; Pan, J. Y.; Settineri, N. S.; Warzok, U.; Bergman, R. G.; Raymond, K. N.; Toste, F. D. Impact of Host Flexibility on Selectivity in a Supramolecular Host-Catalyzed Enantioselective Aza-Darzens Reaction. *J. Am. Chem. Soc.* **2022**, *144* (25), 11425–11433. https://doi.org/10.1021/jacs.2c04182.

(6) Vanicek, S.; Kopacka, H.; Wurst, K.; Müller, T.; Schottenberger, H.; Bildstein, B. Chemoselective, Practical Synthesis of Cobaltocenium Carboxylic Acid Hexafluorophosphate. *Organometallics* **2014**, *33* (5), 1152–1156. https://doi.org/10.1021/om401120h.

(7) Johnson, D. W.; Raymond, K. N. The Self-Assembly of a [Ga4L6]12- Tetrahedral Cluster Thermodynamically Driven by Host-Guest Interactions. *Inorg. Chem.* **2001**, *40* (20), 5157–5161. https://doi.org/10.1021/ic0102283.

(8) Rodríguez, O.; Pence, M. A.; Rodríguez-López, J. Hard Potato: A Python Library to Control Commercial Potentiostats and to Automate Electrochemical Experiments. *Anal. Chem.* **2023**, *95* (11), 4840–4845. https://doi.org/10.1021/acs.analchem.2c04862.

(9) Poletayev, A. D.; McKay, I. S.; Chueh, W. C.; Majumdar, A. Continuous Electrochemical Heat Engines. *Energy Environ. Sci.* **2018**, *11* (10), 2964–2971. https://doi.org/10.1039/c8ee01137k.

(10) Qian, X.; Shin, J.; Tu, Y.; Zhang, J. H.; Chen, G. Thermally Regenerative Electrochemically Cycled Flow Batteries with PH Neutral Electrolytes for Harvesting Low-Grade Heat. *Phys. Chem. Chem. Phys.* **2021**, *23* (39), 22501–22514. https://doi.org/10.1039/d1cp01988k.

(11) Milshtein, J. D.; Barton, J. L.; Darling, R. M.; Brushett, F. R. 4-Acetamido-2,2,6,6-Tetramethylpiperidine-1-Oxyl As a Model Organic Redox Active Compound for Nonaqueous Flow Batteries. *J. Power Sources* **2016**, *327*, 151–159. https://doi.org/10.1016/j.jpowsour.2016.06.125.

(12) Hendriks, K. H.; Sevov, C. S.; Cook, M. E.; Sanford, M. S. Multielectron Cycling of a

Low-Potential Anolyte in Alkali Metal Electrolytes for Nonaqueous Redox Flow Batteries. *ACS Energy Lett.* **2017**, *2* (10), 2430–2435. https://doi.org/10.1021/acsenergylett.7b00559.
(13) Mugridge, J. S.; Szigethy, G.; Bergman, R. G.; Raymond, K. N. Encapsulated Guest-Host Dynamics: Guest Rotational Barriers and Tumbling as a Probe of Host Interior Cavity Space. *J. Am. Chem. Soc.* **2010**, *132* (45), 16256–16264. https://doi.org/10.1021/ja107656g.
